# The role of disease-associated short tandem repeats in amyotrophic lateral sclerosis

**DOI:** 10.1093/braincomms/fcaf482

**Published:** 2025-12-09

**Authors:** Joke J F A van Vugt, Ramona A J Zwamborn, Egor Dolzhenko, Michael A Eberle, Ben Weisburd, Erwin Bekema, Maarten Kooyman, Bi-nan Wang, Project MinE ALS Sequencing Consortium, Philip van Damme, Philippe Corcia, Philippe Couratier, Patrick Vourc'h, Orla Hardiman, Russell McLaughin, Marc Gotkine, Yossef Lerner, Vivian Drory, Nicola Ticozzi, Vincenzo Silani, Jan H Veldink, Leonard H van den Berg, Mamede de Carvalho, Teresa Salas, Jesus S Mora Pardina, Monica Povedano, Peter Andersen, Markus Weber, Nazli A Başak, Ammar Al-Chalabi, Chris Shaw, Pamela J Shaw, Karen E Morrison, John E Landers, Jonathan D Glass, Clifton L Dalgard, Erik-Jan Kamsteeg, Monique Losekoot, Frank Baas, Camilla Novy, Helle Høyer, Ruben P A van Eijk, Michael A van Es, Wouter van Rheenen, Ammar Al-Chalabi, Leonard H van den Berg, Jan H Veldink

**Affiliations:** Department of Neurology, UMC Utrecht Brain Center, University Medical Center Utrecht, Utrecht University, Utrecht 3584 CG, The Netherlands; Department of Neurology, UMC Utrecht Brain Center, University Medical Center Utrecht, Utrecht University, Utrecht 3584 CG, The Netherlands; Illumina Inc., San Diego, CA 92122, USA; Illumina Inc., San Diego, CA 92122, USA; Program in Medical and Population Genetics, Broad Center for Mendelian Genomics, Broad Institute of MIT and Harvard, Cambridge, MA 02142, USA; Center for Genomic Medicine, Massachusetts General Hospital, Harvard Medical School, Boston, MA 02114, USA; Department of Neurology, UMC Utrecht Brain Center, University Medical Center Utrecht, Utrecht University, Utrecht 3584 CG, The Netherlands; Department of Neurology, UMC Utrecht Brain Center, University Medical Center Utrecht, Utrecht University, Utrecht 3584 CG, The Netherlands; Department of Neurology, UMC Utrecht Brain Center, University Medical Center Utrecht, Utrecht University, Utrecht 3584 CG, The Netherlands; Department of Human Genetics, Radboud University Medical Center, Nijmegen 6525 GA, The Netherlands; Department of Clinical Genetics, Leiden University Medical Center, Leiden 2300 RC, The Netherlands; Department of Clinical Genetics, Leiden University Medical Center, Leiden 2300 RC, The Netherlands; Department of Medical Genetics, Telemark Hospital Trust, Skien 3710, Norway; Department of Medical Genetics, Telemark Hospital Trust, Skien 3710, Norway; Department of Neurology, UMC Utrecht Brain Center, University Medical Center Utrecht, Utrecht University, Utrecht 3584 CG, The Netherlands; Department of Neurology, UMC Utrecht Brain Center, University Medical Center Utrecht, Utrecht University, Utrecht 3584 CG, The Netherlands; Department of Neurology, UMC Utrecht Brain Center, University Medical Center Utrecht, Utrecht University, Utrecht 3584 CG, The Netherlands; Department of Basic and Clinical Neuroscience, Maurice Wohl Clinical Neuroscience Institute, King’s College London, London SE5 9RX, UK; Department of Neurology, UMC Utrecht Brain Center, University Medical Center Utrecht, Utrecht University, Utrecht 3584 CG, The Netherlands; Department of Neurology, UMC Utrecht Brain Center, University Medical Center Utrecht, Utrecht University, Utrecht 3584 CG, The Netherlands

**Keywords:** short tandem repeat genotyping, microsatellite genotyping, tandem repeat genotyping accuracy, disease-related repeats, motor neurodegenerative disease

## Abstract

Short tandem repeats (STRs) are recognized contributors to various neurodegenerative disorders, with evidence supporting genetic pleiotropy among these STRs. Multiple STRs have been associated with amyotrophic lateral sclerosis (ALS), although the strength of evidence supporting each association varies. To establish the role of disease-associated repeat expansions as pleiotropic risk factors in ALS susceptibility and progression, we genotyped a panel of 39 STRs, known to cause neurological diseases, within Project MinE in 6519 patients and 2412 controls, utilizing 100 and 150 bp short-read sequencing technology. Pathogenic allele frequencies were compared to those in a control cohort comprising 4930 Genome Aggregation Database (gnomAD) genomes. Repeat sizes and motif changes were detected using ExpansionHunter and ExpansionHunter Denovo. We developed a model to predict genotyping failures in STRs and established a best-practice protocol for assessing the accuracy of STR genotyping in short-read sequencing data. Following our genotyping assessment, 11 out of the 39 STRs exhibited insufficient genotyping accuracy, warranting caution in studying these STRs using these tools in combination with short-read sequencing. Furthermore, the observed differences in STR genotyping accuracy across studies applying different sequencing technologies and genotyping tools in control cohorts highlight the importance of a carefully designed experimental setup when interpreting potential disease-associated STR findings. Pathogenic *C9orf72* and premutated *ATXN2* expansions were confirmed to be significantly associated with ALS susceptibility. Additionally, pathogenic *C9orf72* expansions were significantly associated with reduced mean ALS survival by 11.5 months and an earlier mean age at onset by 2.4 years. Premutation expansions in *ATXN1* showed a nominally significant association with ALS susceptibility, while pathogenic expansions in *NIPA1* displayed a nominally significant association with ALS survival. Previously reported ALS-associated pleiotropy in *HTT* and *STMN2* could not be confirmed. Motif changes were identified in *BEAN1*, *RFC1*, *ATXN8*, *C9orf72*, *DAB1*, *FXN* and *SAMD12*; however, none of the motif changes were linked to ALS. Re-evaluation of clinical data from patients with ALS and a repeat expansion typically associated with another disease revealed that 7% of these patients’ diagnoses had to be reclassified to the disease associated with the repeat expansion (e.g. Kennedy’s disease or spinocerebellar ataxia). This underscores the value of broad STR screening in neurodegenerative cases. Pathogenic and premutation STRs were also found in controls in unexpected high frequencies, suggesting reduced penetrance or underdiagnosis, and highlighting the need for caution when interpreting genetic associations with disease without a proper control cohort.

## Teaser

Van Vugt *et al*. report that genotyping 39 disease-associated short tandem repeats (STRs) in 8531 Project MinE samples revealed pathogenic *C9orf72* and *ATXN2* expansions significantly associated with amyotrophic lateral sclerosis, with *C9orf72* linked to earlier onset and reduced survival. They highlight challenges in STR genotyping, pleiotropy, and careful study design.

## Graphical Abstract

**Graphical Abstract:**
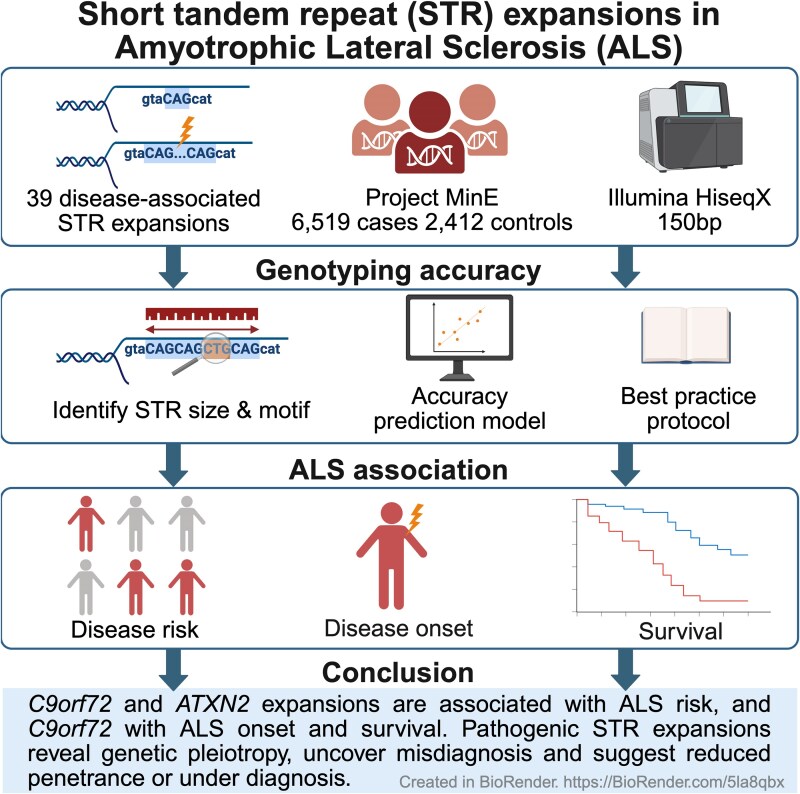

## Introduction

Repetitive DNA sequences comprise over half of the human genome, in contrast to genes and functional elements, which account for just 5%–10%.^1-3^ Tandem repeats, encompassing short tandem repeats (STRs) with 1–6 bp motifs and variable number tandem repeats with > 7 bp motifs, mutate frequently, altering copy number or sequence, and are a major source of human genetic variation.^4^ Over 60 human disorders have been linked to expanded STRs, most of which are neurodegenerative or neuromuscular in nature.^5,6^

Despite their clinical relevance, STR detection is challenging. Traditional large-scale methods like PCR and Southern blotting are labour-intensive, while short-read sequencing, like Illumina, typically has a read length shorter than pathogenic STR expansions. Tools have been developed to genotype STRs longer than the read length in PCR-free short-read sequencing data.^7-12^ ExpansionHunter proved to be the best to accurately estimate the size of both alleles spanning from just a few repeat units to repeat expansions significantly longer than the read length, while being able to consider complex loci with multiple (nearby) STR motifs or sequence interruptions.^13,14^ This tool has been used to genotype disease-associated STRs in large cohorts and genotype unknown STRs in reference genomes.^13,15-19^ Still, STRs are often excluded from routine analyses due to genotyping difficulties, possibly contributing to the ‘missing heritability’ of complex diseases and traits.^20^

To address this, we evaluated STR genotyping sensitivity and specificity using ExpansionHunter and developed a best practice protocol for post-genotyping assessment. We also investigated associations between known neurodegenerative disease-associated STRs and amyotrophic lateral sclerosis (ALS) within Project MinE, the largest ALS whole-genome sequencing effort (6519 patients, 2412 controls).

ALS is a fatal neurodegenerative disorder characterized by progressive degeneration of motor neurons in the brain and spinal cord, with ∼50% heritability.^21,22^ Most of this heritability is considered ‘missing’. Repeat expansions substantially contribute to the genetic cause of ALS. The most common genetic cause of ALS is a hexanucleotide STR in *C9orf72*, found in ∼40% of familial and ∼8% of sporadic ALS cases, depending on the region of origin.^23,24^ Expanded *C9orf72* repeats are also associated with increased risk of frontotemporal dementia (FTD), Parkinson’s, and other movement disorders.^25,26^ Intermediate *ATXN2* expansions (29–33 repeat units) also increase ALS risk, while expansions with more than 33 repeat units cause spinocerebellar ataxia type 2.^27-30^ A recent study further demonstrated the pleiotropy and variable penetrance of *ATXN2* expansions, identifying ALS, SCA2, Parkinsonism, and dementia within the same families and proposing a broader concept of *ATXN2*-related neurodegeneration.^31^ Other repeat expansions implicated as risk factors for ALS include those in *ATXN1*, *NIPA1*, *HTT*, and *STMN2*, though their roles are not yet fully established.^32-35^ Most of these STRs have pervasive genetic pleiotropy given the reported associations with multiple diseases.^36,37^

**Figure 1:**
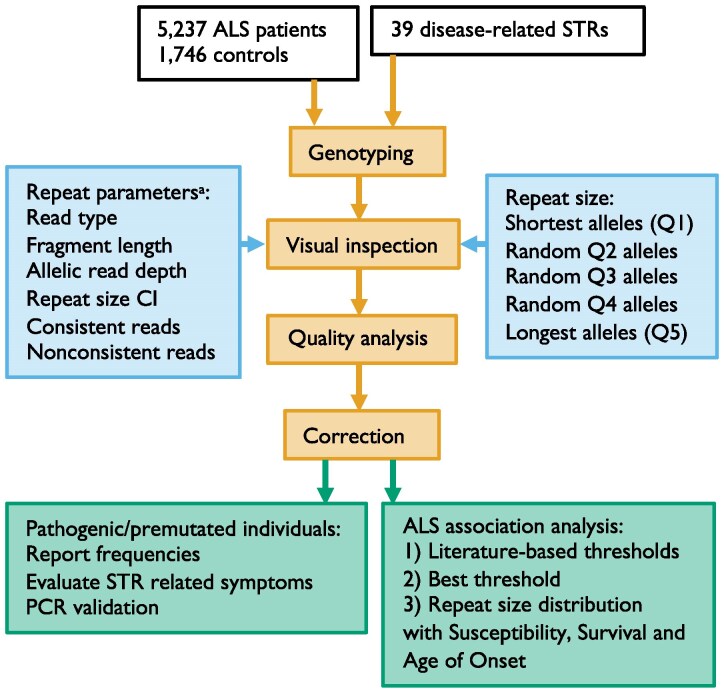
**Genotyping assessment workflow.** Genotyping of 39 disease-associated STRs was assessed using ExpansionHunter v5.0.0 on all Project MinE 150 bp paired-end HiSeqX genomes that passed sample quality control. All alleles were classified according to multiple repeat parameters (a). At most 10 random alleles per repeat parameter of each STR were visually inspected and corrected, if possible. Also, the 25 longest (Q5), 10 shortest (Q1), and 10 random alleles in the 2nd, 3rd and 4th repeat size quintile (Q) were visually inspected and corrected, if possible, using REViewer. The genotyping quality was assessed by building a genotyping accuracy prediction model based on the visual inspection and repeat parameters (a). The genotyping was corrected by setting alleles to missing that were predicted to have a failed genotyping and excluding STRs from further analysis if they either had more than 5% predicted genotyping failures or significantly higher observed genotyping failures than predicted. Pathogenic and premutation frequencies were determined based on disease-associated thresholds from the literature. The clinical symptoms of pathogenic individuals were evaluated, and a portion of expanded and intermediate alleles was validated with PCR. The repeat size was compared between patients and controls, as well as with survival and age at onset in patients, using disease-associated threshold analysis, best threshold analysis and repeat size distribution analysis. ‘Repeat size CI’ indicates the confidence interval for the repeat size.

To explore STR expansions as pleiotropic risk factors in ALS susceptibility and progression, we genotyped 39 STRs and compared allele frequencies with a larger control cohort. We also present a workflow to assess genotyping accuracy (Fig. 1).

## Material and methods

### Whole-genome sequencing and sample quality control

Project MinE’s sequencing and quality-control pipeline is detailed in previous studies.^38,39^ In summary, 1241 cases and 655 matched control samples were sequenced on the Illumina HiSeq 2000 (100 bp paired reads, ∼35× coverage), and 5278 cases and 1757 matched control samples on the Illumina HiSeq X (150 bp paired reads, ∼25× coverage), both using PCR-free library preparation. Data were aligned to hg38 with BWA.^40^ HiSeq 2000 samples were included only for genotyping accuracy comparisons with PCR and Sanger sequencing. Samples failing quality control or up to second-degree relatedness (*n* = 52) were excluded.^39^ For ALS-progression analyses, individuals with invalid, extreme, inconsistent or incomplete survival or age at onset data were removed (*n* = 300, Table S2). Survival, measured in months, was defined as the time from age at onset to death, more than 23 h of ventilation, tracheostomy, or last known follow-up.^41,42^ Patients with an age at onset lower than 18 years or from countries with less than 30% deceased cases were excluded to account for systematic misdiagnosis, i.e. Hereditary Spastic Paraparesis or Primary Lateral Sclerosis (*n* = 569). For demographic and clinical details, see Table S3. Excluded samples showed no significant differences (Table S4). Participant’s consent was obtained according to the Declaration of Helsinki and has been approved by the ethical committee of each institution in which the work was performed.

### STR genotyping

Thirty-nine STRs associated with neurodegenerative disorders were selected (Table S1) and genotyped from whole-genome sequencing data with ExpansionHunter v3.1.2 and v5.0.0.^11,43^ The JSON output was parsed for fragment length and read type, categorized as spanning, flanking and in-repeat reads.^43^ Spanning reads were classified as consistent if their repeat size matched one of the alleles and non-consistent if their repeat size did not match either allele. Flanking and in-repeat reads were classified as consistent if their repeat size matched or was lower than one of the alleles and non-consistent if they exceeded both alleles (Fig. S1). For each allele, the number of consistent and non-consistent reads was determined.

Genotyping data for *ATXN1* and *NIPA1* from Sanger sequencing were obtained from previous studies.^32,33^ Similarly, repeat sizes for *ATXN1*, *ATXN2*, *DMPK*, *HTT* and *NIPA1* were derived from PCR and fragment sizing via gel or capillary electrophoresis, as reported in prior research.^32,33,44-48^  *CSTB* genotyping used PCR with primers FAM-5′-CCCGGAAAGACGATACCAG-3′ and 5′- GAGGAGGCACTTTGGCTTC-3′. For *CSTB* and *DMPK*, repeat-primed PCR and fragment length analysis were performed if only one wild-type allele was detected or an expansion was suspected. *DMPK* repeat-primed PCR followed previous protocols,^48^ while *CSTB* used primers 5′-AGTAGGCGCTGGGGTCAC-3′, 5′-TACGCATCCCAGTTTGAGACGCCCCGCCCCGCG-3′ and FAM-5′- TACGCATCCCAGTTTGAGACG-3′, with longAmp hotstart enzyme mix (New England Biolabs, MA, USA) and addition of ‘GC melt’ (Takara Bio, CA, USA).

The STR genotypes of 4930 PCR-free Genome Aggregation Database (gnomAD) genomes from non-Finnish European origin, excluding neurological and psychiatric cases, served as external controls.^49,50^

### Genotyping accuracy

Read-aligned plots were created with REViewer and independently inspected using Flipbook by JJFAvV and RAJZ.^51^ Genotypes were evaluated based on read alignment quality and quantity in the repeat locus and flanking regions. Poor read alignment or large differences in the number of aligned reads between the repeat and flanks or between both alleles indicated incorrect genotyping.^51^ Genotypes were classified as ‘fail’ if the visual repeat size fell outside the ExpansionHunter confidence interval. Inter-rater agreement was assessed for eight STRs in 992 individuals using the intraclass correlation coefficient and Cohen's kappa.^52^ Agreement was analysed for the sum of both alleles, with sensitivity analysis performed using all intraclass correlation models (‘psych’ R package).

### Genotyping accuracy prediction

A generalized linear model was developed to predict genotyping accuracy, utilizing the visual inspection result ‘pass/fail’ as a binary outcome and repeat parameters as inputs (Fig. 1). To enable the application of a linear model, the repeat parameters were treated as continuous variables, scaled between zero and one. The parameters considered were: (i) ratio of allelic and average read depth (Qdepth, if allelic depth larger than average depth: average depth/allelic depth, else: allelic depth/average depth), (ii) ratio of repeat size and its confidence interval (Qci: 1/exp(4 × confidence interval/repeat size)),^13,53^ (iii) ratio of consistent and total read count (Qcon: consistent reads/total reads), and (iv) ratio of non-consistent and total read count (Qnon: 1/exp(4 × non-consistent reads/total reads)). Calculations were performed with the Python script available at https://github.com/JokevanVugt/EH-STR-parameter-calculator.git. Genomes were split into training (81%, *n* = 3741) and testing (19%, *n* = 850) sets based on sequencing date, with samples before 2018 used for training and those from 2018 onward for testing. This temporal split simulates training on past data and testing on future data. Each read type was analysed separately for model training and genotyping accuracy prediction. Failed genotyping predictions were set to missing. STRs with more than 5% failed genotyping or with significantly more observed failed genotyping than predicted (Chi-square testing, Bonferroni correction) were excluded from further analysis.

### Motif changes

ExpansionHunter Denovo v0.9.0 identified in-repeat reads, reporting read counts per motif and their genomic alignment.^54^ Alignment was based on the anchored read of each pair, limiting motif detection to ∼300–350 bp into the STR in Project MinE genomes. All motif regions overlapping disease-associated STRs were considered for ALS-association analysis. Detected motif changes were validated by visual inspection with REViewer in up to ten random individuals per motif. Motifs consisting of only one repeated nucleotide, for example, 100% C, were excluded. Read counts were compared between patients and controls using Firth's Bias-Reduced Logistic Regression (‘logistf’ R package), adjusting for sex, country and 10 ancestry-informative principal components. Firth’s bias-reduced logistic regression corrects small-sample bias and separation issues, making it suitable for comparing groups with highly unequal counts.

### Association analyses

The association between repeat length and ALS was analysed for susceptibility, survival, and age at onset in three ways: (i) Literature-based threshold analysis, using the disease-associated pathogenic and intermediate thresholds from prior studies and considering the inheritance mode, (ii) Best threshold analysis, testing all observed repeat sizes as threshold, computing allelic dosages, and selecting the lowest *P*-value after correction for the number of repeat sizes tested per STR (‘p.adjust’ R package, method ‘fdr’), and (iii) Repeat size distribution analysis, evaluating maximum allele and sum of alleles in a linear model. *P*-values were Bonferroni corrected for the number of STRs per test and the number of tests per analysis.

Thresholded ALS-susceptibility analyses used Firth’s Bias-Reduced Logistic Regression, adjusting for sex, country, and 10 principal components. Allele frequencies were compared between controls and gnomAD using the same model, adjusting for sex. A generalized linear model (‘glm’ R package) was applied for repeat size distribution analysis of ALS-susceptibility, adjusting for sex, country, and 10 principal components.

For ALS survival, multivariable Cox proportional hazards models (Cox, R package ‘survival’) were applied, adjusting for sex, country and 10 principal components, with survival status used as the censor indicator. STRs with less than five observations per category were removed, and model assumptions were checked using Schoenfeld and martingale residuals. Due to the Cox model’s sensitivity to outliers, the significance threshold for best threshold analyses was set at *P* < 0.01.^55^ The same significance threshold was applied to ALS age at onset, which was analysed using linear regression (‘lm’ R package), adjusting for sex, country and 10 principal components. STRs with less than five observations per category were removed.^56^

Sensitivity analysis included: (i) removing the inheritance mode in literature-based thresholds to account for potential variations in mode of inheritance, (ii) log-transforming repeat sizes to address skewed repeat size distributions, and (iii) applying the Royston-Parmar spline model (‘flexsurv’ R package) to assess survival model robustness, comparing up to five knots. Unlike the Cox model, which assumes proportional hazards, the Royston–Parmar spline model handles time-dependent effects and non-proportional hazards.

### Data availability

STR genotype data from Project MinE underlying this article will be shared on reasonable request to the corresponding author. The code to calculate the STR parameters is available at https://github.com/JokevanVugt/EH-STR-parameter-calculator.git.

## Results

### Genotyping accuracy

To assess genotyping accuracy, PCR and Sanger sequencing results for 6184 samples were compared for *ATXN1*, *ATXN2* and *NIPA1* (Table S5). Genotyping concordance was higher between PCR replicates than between PCR and Sanger sequencing (Table 1). ExpansionHunter showed higher concordance with PCR for genomes sequenced on the HiSeqX platform compared to HiSeq2000, due to both longer read length and improved sequencing quality, as evidenced by short STRs like *NIPA1*. ExpansionHunter v5 also outperformed v3 (Table 1), showing significantly reduced differences and variance in repeat sizes relative to PCR (Fig. S2, Tables S6 and S7). Therefore, only HiSeqX genomes genotyped with ExpansionHunter v5 were used in subsequent analyses.

**Table 1:** Genotyping concordance between wetlab techniques and short-read sequencing

RepeatID	PCR (replicate) % (counts)	Sanger % (counts)	EH v3 HiSeq2000% (counts)	EH v5 HiSeq2000% (counts)	EH v3 HiSeqX % (counts)	EH v5 HiSeqX % (counts)
*ATXN1*	NA	95.0 (1376)	88.1 (2202)	89.4 (2202)	96.8 (950)	99.1 (950)
*ATXN2*	NA	NA	92.5 (1554)	94.2 (1554)	95.2 (542)	96.1 (542)
*NIPA1*	99.7 (952)	97.8 (1336)	87.8 (1664)	91.0 (1664)	97.3 (520)	99.0 (520)
All genes	99.7 (952)	96.3 (2712)	89.3 (5420)	91.3 (5420)	96.5 (2012)	98.3 (2012)

The genotyping concordance of PCR with PCR and Sanger sequencing replicates and short-read sequencing platforms genotyped with ExpansionHunter versions 3.1.2 and 5.0.0 was expressed as a percentage and counts.

### Genotyping best practice workflow

Genotypes of 39 disease-associated STRs (Table S1) were evaluated using the workflow in Fig. 1. Genotyping accuracy and failure were evaluated by visually inspecting aligned reads of alleles flagged by predefined binary repeat parameters, along with a random subset. Alleles that failed genotyping upon visual inspection were compared to the parameters to assess whether these parameters were linked to genotyping failure.

Seven binary repeat parameters were systematically evaluated, and alleles were flagged if they met any of the following criteria: (i) called from flanking reads, indicating the repeat size approximated the read length, or suggesting major indels, (ii) both alleles from in-repeat reads, indicating both were longer than the read length, (iii) potentially exceeding the fragment length, suggesting the repeat size is longer than reported, (iv) allelic read depth five times higher or lower than average, (v) repeat size confidence interval larger than the repeat size, (vi) called from a single consistent read (J1C), and (vii) less consistent than non-consistent reads (LCTNC). Eight STRs had more than 5% of all alleles flagged with a repeat parameter (Fig. S3A).

Visual inspection included 10 random alleles per repeat parameter per STR, the 25 longest (Q5), 10 shortest (Q1), and 10 random alleles from Q2 to Q4. In total, over 6000 alleles were assessed by two researchers (JJFAvV, RAJZ). Incorrect genotypes were corrected when the correct repeat size was clear from the read alignment. Inter-rater agreement, assessed from 922 genotypes, was excellent: Cohen’s kappa was 0.946 (*z* = 144), *P* < 0.0001, and the two-way random effects intraclass correlation coefficient was 0.989 (95% confidence interval (CI) = 0.988–0.99), *P* < 0.0001. Sensitivity analysis showed intraclass correlation coefficients above 0.98 (Table S8).

Conflicting verdicts were mainly limited to genotypes based on one or two reads. *ATXN8* and *FMR1* had the largest number of conflicting verdicts, 83% and 88% agreement compared to 94% overall. This aligned with previous findings of complex genotyping in STRs with consecutive motifs and the known difficulty of sizing the *FMR1* repeat.^51,57^ Failed genotyping frequency was defined as the proportion of alleles receiving a ‘fail’ verdict after visual inspection. Alleles with one or more binary repeat parameters exhibited significantly higher genotyping failure rates than those without (Fig. S3B). However, many flagged alleles were accurately genotyped, and genotyping failures occurred in unflagged alleles, indicating that these binary repeat parameters do not fully explain all instances of genotyping failure.

**Figure 2:**
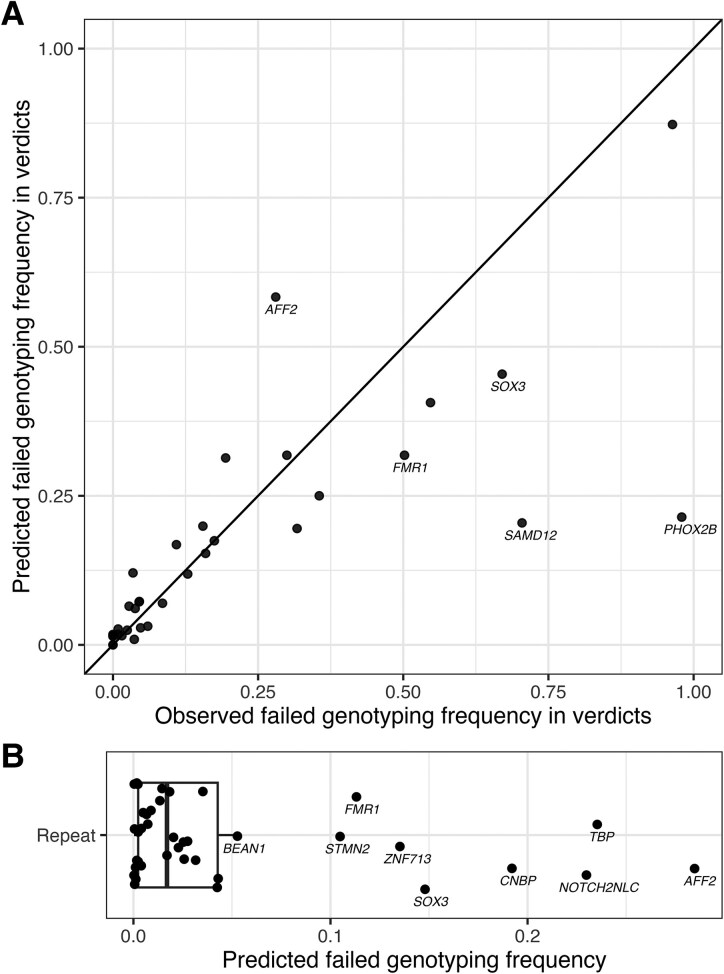
**Genotyping accuracy prediction.** (**A**) Observed versus predicted failed genotyping frequency for each of the 39 disease-associated STRs considering the alleles with a REViewer verdict, not all alleles. *SOX3*, *FMR1*, *SAMD12* and *PHOX2B* had significantly more observed failed genotyping than predicted, based on Chi-square testing and Bonferroni correction (*P*_bon_ < 1 × 10^−7^), and were not considered for further analysis. (**B**) Predicted failed genotyping frequency for each of the 39 disease-associated STRs considering all alleles.

Since individual repeat parameters were insufficient to detect genotyping failures, a predictive model was developed using visual inspection outcomes and repeat parameters (Fig. 1), with 81% of data used for training and 19% for testing. Originally binary, the included parameters were converted to continuous variables: (i) ratio of allelic and average read depth (Qdepth), (ii) ratio of repeat size confidence interval and repeat size (Qci), (iii) ratio of consistent and total read count (Qcon), (iv) ratio of non-consistent and total read count (Qnon), and (v) read type (spanning, flanking, in-repeat), analysed separately. For spanning reads, the model performed best with Qdepth, Qci, and Qnon (AUC: 0.774 training, 0.764 test, Fig. S4). For flanking and in-repeat reads, the model performed best with Qci, Qcon, and Qnon (AUC flanking: 0.701 training, 0.674, test, AUC in-repeat: 0.857 training, 0.862 test). To avoid misclassifying accurate genotypes as failures and to ensure that only STRs with clear evidence of genotyping failure were excluded from downstream analyses, we set the prediction threshold at 90% sensitivity to minimize false negatives. The resulting sensitivity and specificity across STRs revealed significant variation (Fig. S5). While observed and predicted failure frequencies were similar for most STRs (Fig. 2A), five showed significant discrepancies, e.g. *PHOX2B* (false negatives) and *AFF2* (false positives). Applying the model to all alleles, not just the ones visually inspected, the failed genotyping frequency varied by STR (median ∼2%, Fig. 2B). *AFF2* had the highest failure frequency at 28%, largely due to poor read alignment resulting in repeat size overestimation (Fig. S6). This type of inaccurate genotyping occurred in more STRs excluded from disease-association analysis (*FMR1*, *NOTCH2NLC*, *PHOX2B*, *SAMD12*, *SOX*, *STMN2* and *ZNF713*; Table S1, column M). Poor alignment could not be predicted by any single or combined parameter. Other types of inaccurate genotyping identified were indels, motif interruptions, motif changes and mosaicism (Supplementary Note and Figs S20–S25).

Alleles predicted to have failed genotyping or classified under binary repeat parameters were compared to ALS status to detect potential STR genotyping differences beyond repeat size (Table S9). A significant ALS association was detected only for *C9orf72*, involving (i) alleles limited by fragment length (*P* < 2.2 × 10^–16^), (ii) alleles with read counts five times above or below average (*P* < 2.2 × 10^–16^), and (iii) alleles supported by a single consistent read (*P* = 5.8 × 10^–6^). Fragment length limitation and read depth differences stemmed from excess in-repeat reads from the expanded allele, while few consistent reads reflected reduced support for the wild-type allele in the presence of an expansion. The expanded allele likely outcompeted the wild-type during short-read sequencing, inflating intermediate *C9orf72* allele calls when paired with an expansion (Fig. S7). All intermediate *C9orf72* alleles were visually inspected and corrected (Table S10).

Alleles predicted to have failed genotyping were classified as missing. STRs with more than 5% predicted failed genotyping or significantly more observed than predicted failed genotyping were excluded (Fig. 2): *AFF2, BEAN1, CNBP, FMR1, NOTCH2NLC, PHOX2B, SAMD12, SOX3, STMN2, TBP* and *ZNF713*. Repeat size distributions for the 39 disease-associated STRs before and after correction are shown in Fig. S8.

### ALS susceptibility

#### Literature-based threshold analysis

**Figure 3:**
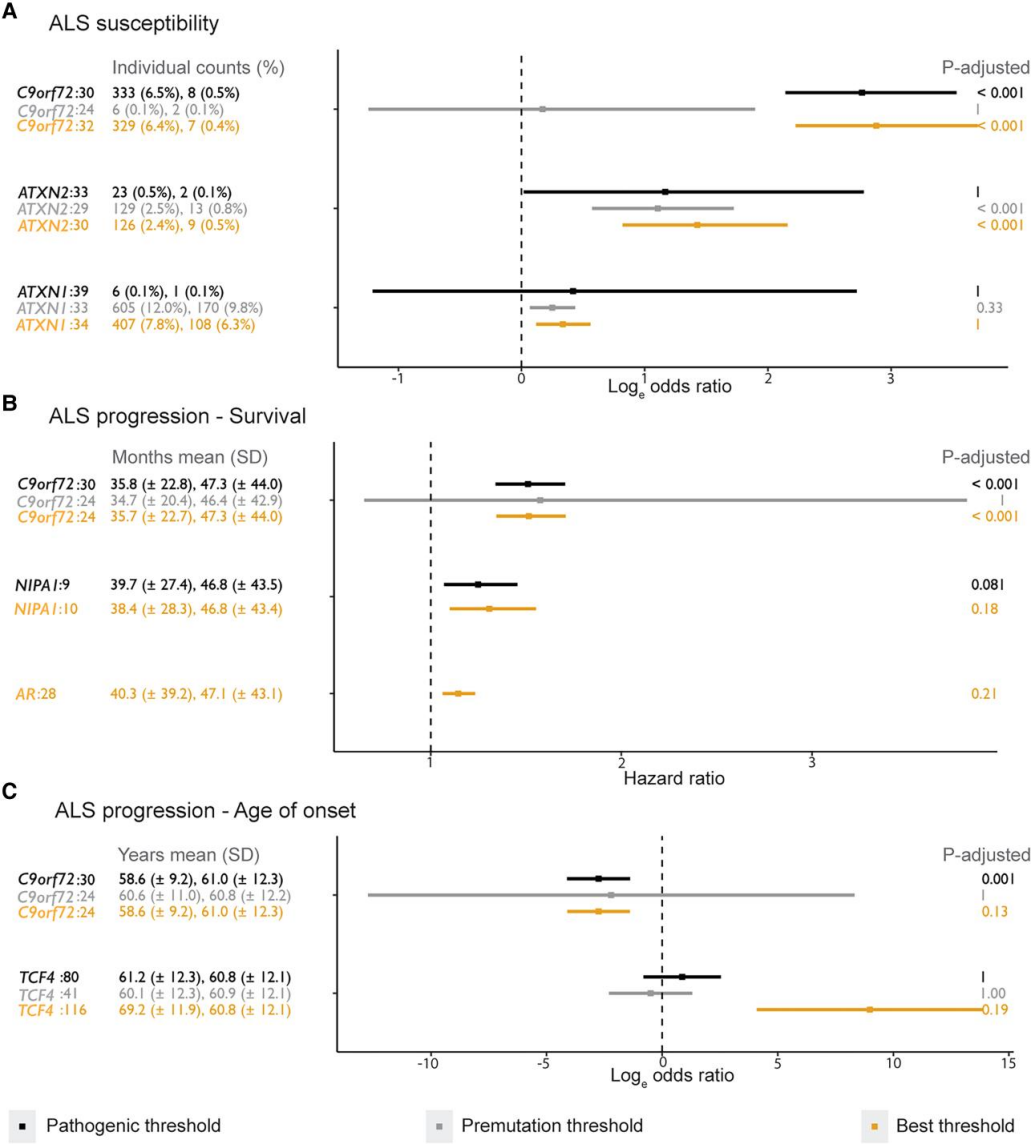
**Association statistics of (nominal) significant STR analyses.** Effect estimates are displayed with error bars representing 95% confidence intervals from (**A**) Firth's bias-reduced logistic regression analysis on ALS susceptibility with expanded case and control numbers and percentages (5237 cases and 1746 controls, Tables S11 and S15), (**B**) Multivariate Cox survival analysis on ALS survival with mean survival and standard deviation (SD) in months of expanded and premutation carriers (*n* = 4,368, Tables S20 and S21, and S24), and (**C**) Linear regression analysis on ALS age at onset with mean age at onset and SD in years of expanded and premutation carriers (*n* = 4,368, Tables S21 and S23, and S25). Colours represent the different types of association analyses performed i.e. pathogenic literature threshold (black), premutation literature threshold (grey) and our data-driven best threshold (orange). The STR name is separated from the tested threshold with a colon. ‘*P*-adjusted’ represents the *P*-value corrected for the number of thresholds and STRs tested (P_bon_ of literature-based threshold analysis and P_fdrbon_ of best threshold analysis).

Repeat size distributions for the 28 disease-associated STRs post-genotyping correction are shown in Fig. S9. Pathogenic and premutation carriers were identified and compared between patients and controls (Table 2, Table S11). Significant associations with ALS were found only for the *C9orf72* pathogenic threshold (≥30, OR = 16, 95% CI = 8.5–34, *P* < 2.2 × 10^−16^) and *ATXN2* premutation threshold (≥29 and <33, OR = 3.0, 95% CI = 1.8–5.6, *P* = 1.4 × 10^−5^) (Fig. 3A, Table S11). Pathogenic *ATXN2* (≥33, *P* = 0.046) and premutated *ATXN1* (≥33, *P* = 0.0069) expansions showed nominally significant associations, and pathogenic *CSTB* expansions were nominally significantly associated when the recessive mode of inheritance associated with Unverricht–Lundborg disease was not considered (≥30, *P* = 0.021, Table S12). Additional *HTT* thresholds showed no nominal association with ALS (Table 3).^58-61^ PCR validation in *CSTB*, *DMPK* and *HTT* confirmed correct genotyping of expanded *DMPK* and *HTT* alleles by ExpansionHunter (Fig. S10). Validated CSTB expansions were misclassified as wild-type by ExpansionHunter, and since not all were limited by the fragment length, fragment length limitation alone is unreliable for detecting repeat size underestimation.

**Table 2:** Percentage of pathogenic and premutation carriers

RepeatID	Disease	Inheritance	Type	Threshold	Cases (%)	Controls (%)	gnomAD (%)	P_PM_	P_gnomad_
*C9ORF72*	ALS/FTD	AD	pathogenic	30	333 (6.5)	8 (0.47)	6 (0.12)	<2.2 × 10^−16^	0.21
		AD	premutation	24	6 (0.12)	2 (0.12)	11 (0.22)	1.00	1.00
*ATXN2*	SCA2	AD	pathogenic	33	23 (0.45)	2 (0.12)	2 (0.041)	1.00	1.00
		AD	premutation	29	129 (2.5)	13 (0.76)	47 (0.95)	6.6 × 10^−4^	1.00
*AR*	SBMA	XR	pathogenic	38	1 (0.019)	1 (0.058)	1 (0.02)	1.00	1.00
		XR	premutation	35	3 (0.058)	1 (0.058)	3 (0.061)	1.00	1.00
*ARX_EIEE*	EIEE	XR	pathogenic	17	0 (0)	0 (0)	11 (0.22)	1.00	1.00
*ARX_PRTS*	PRTS	XR	pathogenic	20	0 (0)	0 (0)	14 (0.29)	1.00	0.83
		XR	premutation	20	0 (0)	0 (0)	0 (0)	1.00	1.00
*ATN1*	DRPLA	AD	pathogenic	48	0 (0)	0 (0)	0 (0)	1.00	1.00
		AD	premutation	36	0 (0)	0 (0)	1 (0.02)	1.00	1.00
*ATXN1*	SCA1	AD	pathogenic	39	6 (0.12)	1 (0.058)	5 (0.1)	1.00	1.00
		AD	premutation	33	605 (12)	170 (9.8)	643 (13)	0.33	0.015
*ATXN3*	SCA3	AD	pathogenic	60	0 (0)	1 (0.057)	0 (0)	1.00	1.00
		AD	premutation	45	0 (0)	1 (0.057)	0 (0)	1.00	1.00
*ATXN8*	SCA8	AD	pathogenic	80	55 (1.1)	18 (1.0)	52 (1.1)	1.00	1.00
		AD	premutation	51	28 (0.54)	7 (0.40)	26 (0.53)	1.00	1.00
*CACNA1A*	SCA6	AD	pathogenic	20	1 (0.019)	0 (0)	1 (0.02)	1.00	1.00
		AD	premutation	19	0 (0)	0 (0)	1 (0.02)	1.00	1.00
*DMPK*	DM1	AD	pathogenic	50	5 (0.096)	2 (0.11)	1 (0.02)	1.00	1.00
		AD	premutation	35	29 (0.55)	12 (0.69)	19 (0.39)	1.00	1.00
*GIPC1*	OPDM2	AD	pathogenic	73	2 (0.039)	0 (0)	1 (0.02)	1.00	1.00
		AD	premutation	32	7 (0.14)	4 (0.23)	12 (0.24)	1.00	1.00
*HTT*	HD	AD	pathogenic	40	5 (0.096)	1 (0.058)	0 (0)	1.00	1.00
		AD	premutation	27	316 (6.1)	105 (6.1)	308 (6.2)	1.00	1.00
*NIPA1*	HSP6	AD	pathogenic	9	232 (4.6)	78 (4.6)	242 (4.9)	1.00	1.00
*NOP56*	SCA36	AD	pathogenic	650	1 (0.019)	0 (0)	0 (0)	1.00	1.00
*PABPN1*	OPMD	AD	pathogenic	8	2 (0.038)	2 (0.11)	18 (0.37)	1.00	1.00
*PPP2R2B*	SCA12	AD	pathogenic	43	0 (0)	0 (0)	1 (0.02)	1.00	1.00
		AD	premutation	33	0 (0)	0 (0)	1 (0.02)	1.00	1.00
*RFC1*	CANVAS	AR	pathogenic	400	309 (6.0)	96 (5.6)	NA	1.00	NA
*TCF4*	FECD3	AD	pathogenic	80	228 (4.4)	77 (4.5)	137 (2.8)	1.00	0.021
		AD	premutation	41	200 (3.9)	79 (4.6)	252 (5.1)	1.00	1.00

These numbers were based on literature thresholds and disease-associated mode of inheritance. STRs without pathogenic and premutation carriers were not considered, i.e. *ATXN7*, *ATXN10*, *CBL*, *CSTB*, *FXN*, *GLS*, *JPH3* and *LRP12*. STRs excluded from disease-association analysis after genotyping assessment were: *AFF2*, *BEAN1*, *CNBP*, *FMR1*, *NOTCH2NLC*, *PHOX2B*, *SAMD12*, *SOX3*, *STMN2*, *TBP* and *ZNF713*. In Project MinE, fragment length-limited alleles were considered pathogenic if they were shorter than the pathogenic threshold. This assessment was not possible in gnomAD due to unavailable individual fragment lengths, so pathogenic *RFC1* expansions were marked as ‘NA’. XR is X-linked recessive inheritance. AD is an autosomal dominant inheritance. AR means autosomal recessive inheritance. P_PM_ is the *P*-value of the association between Project MinE cases and Project MinE controls, Bonferroni corrected for the number of STRs and thresholds tested per STR. P_gnomad_ is the *P*-value of the association between Project MinE controls and gnomAD controls, Bonferroni corrected for the number of STRs and thresholds tested per STR.

**Table 3:** Number of pathogenic and premutation carriers in additional HTT thresholds

RepeatID	Type	Threshold	Cases (%)	Controls (%)	*P*	Pbon	OR	95% CI	gnomAD (%)
*HTT*	premutation	≥ 27 & < 35	295 (5.7)	100 (5.8)	0.99	1.00	1.00	0.79–1.27	296 (6.0)
*HTT*	premutation	≥ 27 & < 36	305 (5.9)	101 (5.8)	0.89	1.00	1.02	0.80–1.29	301 (6.1)
*HTT*	premutation	≥ 36 & < 40	11 (0.21)	4 (0.23)	0.61	1.00	0.74	0.25–2.55	7 (0.14)
*HTT*	pathogenic	≥ 37	9 (0.17)	4 (0.23)	0.54	1.00	0.69	0.23–2.42	4 (0.081)

Additional pathogenic and premutation thresholds reported for *HTT* expansions associated with Huntington’s disease were analysed in Project MinE for association with ALS. *P* represents the uncorrected *P*-value of the association between cases and controls. P_bon_ is the *P*-value of the association between cases and controls, Bonferroni corrected for the number of thresholds tested.

Several patients with pathogenic STR expansions also carried a pathogenic *C9orf72* expansion, including one of two with a *GIPC1* expansion and one with an *AR* expansion (compare Tables S11 and S13). Clinical re-evaluation of ALS patients with STRs linked to other disorders (Table S14) identified four misdiagnosed cases: one with Spinocerebellar ataxia type 36, one with Friedereich's ataxia, one male patient with Spinal and bulbar muscular atrophy, and one person with Oculopharyngodistal myopathy type 2. The remaining 52 patients had typical ALS. The only ALS patient with a pathogenic *NOP56* expansion had second-degree relatives with ataxia.

Significant differences in pathogenic and premutated STR frequencies were observed between Project MinE and gnomAD controls (Table 2). The higher number of pathogenic *TCF4* alleles in Project MinE was due to longer average fragment lengths (450 versus 361 bp; Fig. S11A), as ExpansionHunter underestimates repeat sizes exceeding the fragment length (Fig. S11B), causing a significant number of *TCF4* alleles in gnomAD to fall below the pathogenic threshold. The higher *ATXN1* premutation frequency in gnomAD likely stemmed from false expansions not corrected as they were in Project MinE (Fig. S12). Pathogenic *ARX* expansions in gnomAD were due to genotyping failures from degenerate STR motifs, an issue avoided in Project MinE. These discrepancies between Project MinE and gnomAD highlight the need for careful experimental design and genotyping validation.

#### Best threshold analysis

Firth's bias-reduced logistic regression identified significant associations of *C9orf72* and *ATXN2* repeat expansions with ALS susceptibility (Fig. 3A, Table S15). The optimal threshold for *C9orf72* was 32 repeat units (OR = 17.8, 95% CI = 9.2–40.5, *P*_FDR_ < 2.2 × 10^–16^), though a significant association with ALS was already observed at repeat lengths above 22 (Fig. S13). This earlier association likely reflects the limited number of samples with intermediate-sized alleles and suggests uncertainty in determining an exact best threshold for *C9orf72* (Table S10). The best thresholds of *ATXN2* and *ATXN1* were the same as the intermediate thresholds established in spinocerebellar ataxia type 2 and type 1, i.e. larger than 29 and 33 repeat units, respectively, though this was only significant for *ATXN2* (OR = 4.2, 95% CI = 2.3–8.7, *P*_FDR_ = 5.7 × 10^–6^).^27,33^  *ATXN2* was also significantly associated with ALS between 28–32 repeat units (OR = 3.0–7.5, 95% CI = 1.9–36, *P*_FDR_ = 1.4 × 10^−4^–8.5 × 10^−6^; Fig. S14). *ATXN1* showed a weaker association in the 32–34 repeat unit range (OR = 1.2–1.4, 95% CI = 1.05–1.75, *P*_FDR_ = 0.051; Fig. S15).

A *CSTB* threshold with more than 19 repeat units identified 20 patients, with no controls, exceeding this size. However, expansion frequencies were similar in gnomAD (18/4390; 1/274) and Project MinE patients (20/5224; 1/261), with no age differences (Fig. S16). Relatedness analysis using TRIBES showed that the 20 patients were unrelated up to the 6th degree.^62^ REViewer confirmed the gnomAD expansions as genuine.^63^ Project MinE and non-Finnish European gnomAD samples show similar ancestry, though geographical variation may exist (Table S4).^64^ Despite excluding known neurological cohorts from gnomAD, undiagnosed cases may remain. *RFC1* and *DAB1* also showed suggestive associations with ALS (ORs and 95% CIs > 1), with best thresholds near fragment length, possibly reflecting ALS susceptibility to repeat expansions longer than the fragment length (Table S15).^65,66^

ExpansionHunter Denovo detected numerous motif changes, especially in *BEAN1* and *RFC1*, and also in *ATXN8*, *C9orf72*, *DAB1*, *FXN* and *SAMD12* (Table S16). Out of 36 identified motif changes, only three were previously associated with disease, all in *RFC1*.^67^ Motif changes did not occur significantly more frequently in patients than in controls, whether analysed individually, per STR, or in potentially pathogenic *RFC1* genotypes (Tables S16 and S17).

#### Repeat size distribution analysis

Generalized linear modelling showed significant differences in maximum repeat size distributions between patients and controls for *ATXN1*, *ATXN2* and *C9orf72* (*P* = 2.8 × 10^–4^, 4.2 × 10^–4^ and 1.8 × 10^–11^, respectively; Table S18). For *C9orf72*, the sum of both alleles also differed significantly (*P* = 1.5 × 10^–11^). Sensitivity analysis using log-transformed repeat sizes confirmed these findings (Table S19).

### ALS progression

#### Literature-based threshold analysis

Multivariate Cox survival analysis identified a significant association between pathogenic *C9orf72* expansions (≥30 repeat units) and reduced ALS survival (HR = 1.51, 95% CI = 1.34–1.71, *P* = 2.37 × 10^−11^), with carriers living on average 11.5 months less corresponding to a median survival difference of 3.8 months (Fig. 3B, Fig. S17, Tables S20 and S21). No other STRs or *HTT* thresholds were significantly associated with survival (Table S22), though *NIPA1* expansions (≥9 repeat units) showed a nominal association (*P* = 0.005), linked to an average of 7.1-month shorter survival (median = 3.2 months).

Linear regression analysis of age at onset with disease-associated pathogenic and premutation thresholds identified *C9orf72* as the only significant modifier (≥30 repeat units; OR = 0.064, 95% CI = 0.016–0.25, *P* = 7.96 × 10^−5^; Fig. 3C, Tables S21 and S23), with carriers showing earlier onset (mean 58.6 ± 9.14 years) compared to non-carriers (mean = 61 ± 12.3 years) corresponding to a median difference of 3.1 years.

#### Best threshold analysis

Best threshold Cox analysis revealed a significant survival association with *C9orf72,* which was strongest at 24 repeat units (HR = 1.52, 95% CI = 1.34–1.71, *P*_FDR_ = 6.65 × 10^−11^), though a significant association with ALS survival was already observed at repeat lengths above 13 (Fig. 3B, Fig. S18). *C9orf72* expansions exceeding 24 repeat units had an average survival reduction of 11.6 months, corresponding to a median survival difference of 3.7 months (Table S21). Additional significant associations were found for *NIPA1* and *AR* when correcting only for thresholds tested per STR (Fig. 3B, Table S24).

Best threshold age at onset analysis found no associations when correcting for all thresholds across all STRs. However, a significant effect at *C9orf72* was observed when correcting only for the thresholds tested in this locus (≥24 repeat units, OR = 0.064, 95% CI = 0.017–0.25, *P*_FDR_ = 5.26 × 10^−3^; Fig. 3C, Tables S21 and S25, Fig. S19), with more than 23 repeat units linked to a 2.4-year earlier onset corresponding to a median difference of 3.1 years. A similar significant association to age at onset was found for *TCF4* (≥116 repeat units, *P*_FDR_ = 7.79 × 10^−3^), though the accuracy of this result is limited, as most alleles exceeding 116 repeat units were constrained by the fragment length.

#### Repeat size distribution analysis

Analysis of maximum repeat size revealed significant associations with ALS survival in *C9orf72* and *AR* (*P* = 1,47 × 10^−8^ and *P* = 5.89 × 10^−4^, respectively; Table S26A). The sum of both alleles showed a significant survival association only in *C9orf72* (*P* = 1.12 × 10^−8^; Table S26B).

To validate the *AR* finding, we tested an independent Norwegian ALS cohort (*n* = 568), defining survival as time from onset to death or last contact due to lack of ventilation data.^68^ No significant associations were found with *AR* repeat size (max: *P* = 0.38; best threshold ≥28: HR = 0.98, 95% CI = 0.94–1.01, *P* = 0.22; Table S27).

Linear regression for age at onset found no significant associations, though *C9orf72* showed a nominal inverse relationship between maximum repeat length and age at onset (*P* = 1.13 × 10^−3^; Table S28).

Sensitivity analyses confirmed the robustness of ALS progression results across different strategies, including use of a Royston–Parmar flexible parametric survival model with two knots (Table S29A and B), exclusion of literature-based inheritance assumptions (Table S29C–F), and analysis of log-transformed repeat sizes (Table S29G and H).

## Discussion

This study presents a comprehensive profile of 39 STRs associated with neurological disorders in the largest sporadic ALS cohort to date. We confirmed the association between *C9orf72* and *ATXN2* and ALS susceptibility, with the best thresholds aligning with those previously reported.^27,44,57,69,70^ Progression analysis validated *C9orf72* as a modifier of ALS survival and age at onset, again with thresholds consistent with earlier findings.^71-73^ We found no compelling evidence that other STR loci are associated with ALS, even when considering repeat lengths shorter or longer than established pathogenic thresholds. Re-evaluation of clinical data of patients carrying pathogenic STRs other than *C9orf72* and *ATXN2* revealed that 7% did not have ALS, underscoring the value of genetic screening in patients with neurodegenerative symptoms. Still, pathogenic and premutated STRs were observed in both cases and controls not diagnosed with the associated diseases, in line with previous observations, that frequencies of pathogenic repeat expansions were higher than expected.^19^ This suggests reduced penetrance or potential underdiagnosis and advises caution when interpreting disease association based solely on genetic data. Lastly, our study underscores the importance of STR genotyping quality assessment when using short-read sequencing.

Our genotyping workflow combined visual inspection of aligned reads with a predictive model assessing genotyping accuracy based on multiple repeat parameters. Genotyping STRs from short-read whole-genome sequencing has improved substantially in the last decade. We demonstrated the superior performance of ExpansionHunter v5 over v3, validated by PCR and Sanger sequencing in over 5600 samples. Additionally, genomes sequenced with HiSeqX yielded more accurate genotypes than those sequenced with HiSeq2000, due to longer reads and higher sequencing quality. Despite these improvements, STR genotyping assessment remains essential. We identified various types of genotyping failures, i.e. structural variants, more than two repeat sizes per sample, and poor read alignment, that each can distort estimates of pathogenic or premutation frequencies and disease association. While ExpansionHunter is designed to reduce false negatives by tolerating lower alignment quality and uneven allele coverage, it can introduce false positives. No current tool can perfectly genotype all alleles across any STR, making manual review necessary.

Because repeat parameters were generally unrelated to specific genotyping failures, each STR requires individual genotyping accuracy assessment. Visual inspection of aligned reads across a representative sample of the repeat size and binary repeat parameters helps estimate genotyping failure types and their magnitude. Interestingly, significant case-control differences in repeat parameters can reveal disease-associated variation not captured by repeat size alone. In our study, only *C9orf72* showed such differences, which were primarily due to genotyping errors in intermediate-sized repeats, especially when co-occurring with an expanded allele. Previous reports of significant ALS associations in heterozygous and homozygous premutated *C9orf72* carriers were not supported by our findings, even when using uncorrected genotypes (Table S10).^74-76^ The significant association between ALS and the sum of both *C9orf72* alleles can therefore be attributed primarily to the longest allele. Given the difficulty in accurately genotyping intermediate *C9orf72* expansions, alternative methods beyond short-read sequencing are recommended for assessing their role in ALS.

Despite limitations in detecting motif changes beyond 300–350 bp into the STR locus, we observed many novel motifs. Though none were associated with ALS, some may be relevant to other diseases. Though pathogenic motif changes were observed in *RFC1*, short read sequencing limitations prevented establishing whether they were accompanied by pathogenic repeat sizes. The pathogenic *DAB1* motif change lies deep within the repeat locus, making it inaccessible to ExpansionHunter Denovo. Notably, the best thresholds associated with ALS susceptibility for *DAB1*, *RFC1* and *CSTB,* and with age at onset for *TCF4* were in the fragment length range. Disease associations close to or beyond this range could be misinterpreted or missed and require alternative STR genotyping techniques, such as long-read sequencing.

The STR in *STMN2*, previously linked to ALS or ALS survival, failed our genotyping accuracy assessment and was not associated with ALS in other studies.^35,77,78^ The higher number of ALS patients with 24 CA repeat units reported in Theunissen *et al*. was not replicated, potentially due to tissue-specific differences (blood versus spinal cord motor neurons).^35^ Genotyping difficulties in *STMN2* indicate that accurately determining its repeat size remains challenging across multiple methods. Although reduced *STMN2* expression likely contributes to ALS pathology, the role of repeat length warrants further investigation.^79^

Although *HTT* expansions have been reported as an ALS risk factor, we found no significant association with ALS susceptibility or progression.^34^ This does not support pleiotropy of *HTT* expansions as observed by others.^34,58,60,61,68,80-82^ Nonetheless, pathogenic *HTT* carrier frequency was approximately three times higher in patients (0.10%) than in multiple population-based cohorts (0% in gnomAD, 0.03% in 100KG and TOPMed, and 0.04% in five European population-based cohorts).^19,59^ This aligns with prior studies reporting *HTT* expansions in FTD/ALS patients with classical TDP-43 pathology and huntingtin-positive aggregates.^34^ Notably, *C9orf72* expansions are also a frequent cause of Huntington’s disease phenocopies, reflecting the complexity of genotype-phenotype relationships.^83^ Further research is needed to determine whether pleiotropy or functional overlap explains the observed *HTT*-ALS associations, especially considering ancestry-based allele frequency differences.^19^

Genetic pleiotropy has been reported across neurodegenerative diseases in both early linkage studies and recent GWAS.^69,84,85^ ALS shares pleiotropic associations with FTD, spinocerebellar ataxias, hereditary spastic paraplegia, Huntington’s, and Alzheimer’s disease. We validated the known pleiotropic association of *C9orf72* and *ATXN2* with ALS.^27,44,69,70^ While *ATXN1* premutations were strongly associated with ALS, the association did not reach significance as reported previously, despite including overlapping data in both studies.^33^ This may be attributed to sample size differences. A novel association was observed between *AR* repeat length and ALS survival, which may relate to androgen biology, as androgen ablation can extend survival and disease duration in *SOD1* ALS mouse models.^86^ Since changes in CAG repeat lengths in *AR* have been associated with changes in androgen levels, this could imply a possible role in ALS survival.^87^ However, this was not replicated in a smaller Norwegian cohort, indicating further research is needed. Contrary to earlier studies, we did not observe an association of *NIPA1* with ALS susceptibility or age at onset.^32,45,88,89^ The survival association we identified adds to the conflicting literature, highlighting the need for additional replication studies to clarify the role of *NIPA1* in (*C9orf72*-associated) ALS.^32,45^

Variability in reported thresholds and uncertainty in sizing STRs beyond the read length can significantly affect the number of individuals inferred to be at risk. Differences in thresholds may stem from population-specific disease prevalence and penetrance, as well as study design.^19,23^ This highlights the importance of careful experimental setups when evaluating STR-disease associations. Long-read sequencing will be critical for more accurate repeat sizing, while also accounting for motif changes, interruptions, and the complexity of the flanking sequence.^6,14,90,91^ Given the increasing likelihood of an (ultra-)rare genetic cause of ALS, large harmonized whole-genome sequencing datasets with diverse ancestries are essential to improve power and generalizability. At present, large-scale long-read sequencing appears infeasible due to both cost and challenges in data collection. A more promising approach is to integrate large short-read sequencing cohorts across diverse populations, such as Project MinE with UK Biobank, FinnGen, and AllOfUs, and complement this with targeted validation of potential repeat expansions using long-read sequencing.^40^

## Supplementary Material

fcaf482_Supplementary_Data

## Appendix I

Project MinE ALS Sequencing Consortium: Philip van Damme, Philippe Corcia, Philippe Couratier, Patrick Vourc'h, Orla Hardiman, Russell McLaughin, Marc Gotkine, Yossef Lerner, Vivian Drory, Nicola Ticozzi, Vincenzo Silani, Jan H. Veldink, Leonard H. van den Berg, Mamede de Carvalho, Teresa Salas, Jesus S. Mora Pardina, Monica Povedano, Peter Andersen, Markus Weber, Nazli A. Başak, Ammar Al-Chalabi, Chris Shaw, Pamela J. Shaw, Karen E. Morrison, John E. Landers, Jonathan D. Glass, Clifton L. Dalgard.
