# Supplementary material for: The role of disease-associated short tandem repeats in amyotrophic lateral sclerosis
Source: Brain Commun. 2025 Dec 9;7(6):fcaf482. doi: 10.1093/braincomms/fcaf482 (PMC12715773; doi:10.1093/braincomms/fcaf482)
Supplement: fcaf482_Supplementary_Data [file fcaf482_supplementary_data.zip › Supplementary_material.docx]

**­­Supplementary data for:**

**The role of disease-associated short tandem repeats in Amyotrophic Lateral Sclerosis**

Joke J.F.A. van Vugt^1,†^, Ramona A.J. Zwamborn^1,†^, Egor Dolzhenko^2^, Michael A. Eberle^2^, Ben Weisburd^3,4^, Erwin Bekema^1^, Maarten Kooyman^1^, Bi-nan Wang^1^, Project MinE ALS Sequencing Consortium, Erik-Jan Kamsteeg^5^, Monique Losekoot^6^, Frank Baas^6^, Camilla Novy^7^, Helle Høyer^7^, Ruben P.A. van Eijk^1^, Michael A. van Es^1^, Wouter van Rheenen^1^, Ammar Al-Chalabi^8^, Leonard H. van den Berg^1^, Jan H. Veldink^1^

^†^Joke van Vugt and Ramona Zwamborn contributed equally to this work.

**Author affiliations**

1 Department of Neurology, UMC Utrecht Brain Center, University Medical Center Utrecht, Utrecht University, 3584 CG, Utrecht, The Netherlands

2 Illumina Inc., San Diego, CA 92122, USA

3 Program in Medical and Population Genetics, Broad Center for Mendelian Genomics, Broad Institute of MIT and Harvard, Cambridge 02142, MA, USA

4 Center for Genomic Medicine, Massachusetts General Hospital, Harvard Medical School, Boston, MA 02114, USA

5 Department of Human Genetics, Radboud University Medical Center, 6525 GA, Nijmegen, The Netherlands.

6 Department of Clinical Genetics, Leiden University Medical Center, 2300 RC, Leiden, The Netherlands

7 Department of Medical Genetics, Telemark Hospital Trust, 3710 Skien, Norway

8 Maurice Wohl Clinical Neuroscience Institute, King's College London, Department of Basic and Clinical Neuroscience, London SE5 9RX, UK

**Correspondence to:** Joke J.F.A. van Vugt

Department of Neurology, UMC Utrecht Brain Center, University Medical Center Utrecht, Utrecht University, Universiteitsweg 100, 3584 CG, Utrecht, The Netherlands

j.f.a.vanvugt-2@umcutrecht.nl

**Content: Page Nr.**

Supplementary Tables and Figures 2 - 46

Supplementary Note 47

Supplementary Figures to Supplementary Note 48 - 53

References to Supplementary Note 54

**Supplementary Tables and Figures**

**Supplementary Table 2. Survival and age at onset selection criteria.**

| **Comparison** | **Cutoff** |
| --- | --- |
| Diagnosis == 'Control' checked for Date of Diagnosis | is available Date of Diagnosis |
| Diagnosis == 'Control' checked for Date of Onset | is available Date of Onset |
| ALSnr rerun checked for Diagnosis | Different Diagnosis |
| ALSnr rerun checked for Gender | Different Gender |
| Age at Blood draw | Value outside 18 to 110 |
| Age at Onset | Value outside 18 to 110 |
| Age at Death | Value outside 18 to 110 |
| Age at Ventilation > 23h | Value outside 18 to 110 |
| Age at Diagnosis | Value outside 18 to 110 |
| Date of Birth | Value outside 01-01-1896 to current data |
| Date of Diagnosis | Value outside 01-01-1951 to current data |
| Date of Onset | Value outside 01-01-1950 to current data |
| Date of Check | Value outside 01-01-1980 to current data |
| Date of Death | Value outside 01-01-1980 to current data |
| Date of Blood draw | Value outside 01-01-1980 to current data |
| Date of FVC | Value outside 01-01-1980 to current data |
| Date of Ventilation > 23h | Value outside 01-01-1980 to current data |
| FVC percentage | Value outside 20 to 200 |
| FVC liter | Value outside 0 to 7.5 |
| ALSFRS.R total score | Value outside 1 to 48 |
| ALS first degree family | Value outside 0 to 10 |
| ALS second degree family | Value outside 0 to 10 |
| FTD first degree family | Value outside 0 to 10 |
| FTD second degree family | Value outside 0 to 10 |
| ALSFRS.R Diagnosis | Value outside 1 to 48 |
| Diagnosis Delay | Value outside 0 to 20 |
| Order: Date of Birth Date of Onset | Date of Birth after Date of Onset |
| Order: Date of Birth Date of Diagnosis | Date of Birth after Date of Diagnosis |
| Order: Date of Birth Date of Death | Date of Birth after Date of Death |
| Order: Date of Birth Date of Check | Date of Birth after Date of Check |
| Order: Date of Birth Date of Blood draw | Date of Birth after Date of Blood draw |
| Order: Date of Birth Date of FVC | Date of Birth after Date of FVC |
| Order: Date of Onset Date of Diagnosis | Date of Onset after Date of Diagnosis |
| Order: Date of Onset Date of Death | Date of Onset after Date of Death |
| Order: Date of Onset Date of Check | Date of Onset after Date of Check |
| Order: Date of Onset Date of Blood draw | Date of Onset after Date of Blood draw |
| Order: Date of Onset Date of FVC | Date of Onset after Date of FVC |
| Order: Date of Diagnosis Date of Death | Date of Diagnosis after Date of Death |
| Order: Date of Diagnosis Date of Check | Date of Diagnosis after Date of Check |
| Age Onset Age at Diagnosis | Age at Onset after Age at Diagnosis |
| Age Onset Age at Blood draw | Age at Onset after Age at Blood draw |
| Age Onset Age at Death | Age at Onset after Age at Death |
| Age Diagnosis Age at Death | Age at Diagnosis after Age at Death |
| Age Blood draw Age at Death | Age at Blood draw after Age at Death |
| Age at onset | <= 18 |
| Survival in months | < 0 |

The abbreviation FVC stands for Forced Vital Capacity, and ALSFRS.R for Amyotrophic Lateral Sclerosis Functional Rating Scale Revised.

**Supplementary Table 3. Demographic and clinical characteristics of the study population separated by diagnosis.**

|  | **Case**  N=5237 | **Control**  N=1746 |
| --- | --- | --- |
| **Sex** |  |  |
| Female | 2092 (39.9%) | 852 (48.8%) |
| Male | 3141 (60.0%) | 894 (51.2%) |
| Missing | 4 (0.1%) | 0 (0%) |
| **Age at blood draw in years** |  |  |
| Mean (SD) | 61.6 (12.3) | 60.5 (12.1) |
| Median [Min, Max] | 62.7 [14.9, 100] | 61.9 [18.0, 98.0] |
| Missing | 1218 (23.3%) | 212 (12.1%) |
| **Age at onset in years** |  |  |
| Mean (SD) | 60.8 (12.1) |  |
| Median [Min, Max] | 62.1 [18.0, 98.8] |  |
| Missing | 833 (15.9%) |  |
| **Site of Onset** |  |  |
| Bulbar | 1166 (22.3%) |  |
| Spinal | 3175 (60.6%) |  |
| Missing | 896 (17.1%) |  |
| **Survival status** |  |  |
| alive | 884 (16.9%) |  |
| dead | 3520 (67.2%) |  |
| Missing | 833 (15.9%) |  |
| **Survival in months** |  |  |
| Mean (SD) | 46.5 (42.8) |  |
| Median [Min, Max] | 34.0 [1.35, 554] |  |
| Missing | 833 (15.9%) |  |
| **El Escorial** |  |  |
| definite | 1222 (23.3%) |  |
| possible | 376 (7.2%) |  |
| probable | 1631 (31.1%) |  |
| suspected | 47 (0.9%) |  |
| Missing | 1961 (37.4%) |  |

Shown are numbers (and percentages) of samples that passed quality control. The variability of the data around the mean is expressed as the standard deviation (SD).

**Supplementary Table 4. Demographic and clinical characteristics of the removed samples separated by diagnosis.**

|  | **Case**  **N=869** | **Control**  **N=1746** |
| --- | --- | --- |
| **Sex** |  |  |
| female | 363 (41.8%) | 852 (48.8%) |
| male | 502 (57.8%) | 894 (51.2%) |
| Missing | 4 (0.5%) | 0 (0%) |
| **Age at onset years** |  |  |
| Mean (SD) | 59.7 (9.79) |  |
| Median [Min, Max] | 59.8 [27.5, 74.5] |  |
| Missing | 833 (95.9%) |  |
| **Age at blood draw in years** |  |  |
| Mean (SD) | 55.6 (13.7) | 60.5 (12.1) |
| Median [Min, Max] | 57.0 [14.9, 100] | 61.9 [18.0, 98.0] |
| Missing | 176 (20.3%) | 212 (12.1%) |
| **Site of Onset** |  |  |
| bulbar | 7 (0.8%) |  |
| spinal | 29 (3.3%) |  |
| Missing | 833 (95.9%) |  |
| **Survival status** |  |  |
| alive | 4 (0.5%) |  |
| dead | 32 (3.7%) |  |
| Missing | 833 (95.9%) |  |
| **Survival months** |  |  |
| Mean (SD) | 49.8 (31.5) |  |
| Median [Min, Max] | 40.3 [16.3, 136] |  |
| Missing | 833 (95.9%) |  |
| **Origin** |  |  |
| Belgium | 9 (1.0%) | 178 (10.2%) |
| Spain | 2 (0.2%) | 153 (8.8%) |
| France | 30 (3.5%) | 38 (2.2%) |
| Great Britain | 77 (8.9%) | 439 (25.1%) |
| Ireland | 6 (0.7%) | 232 (13.3%) |
| Italy | 18 (2.1%) | 0 (0%) |
| The Netherlands | 9 (1.0%) | 384 (22.0%) |
| Sweden | 3 (0.3%) | 111 (6.4%) |
| Turkey | 606 (69.7%) | 132 (7.6%) |
| United States | 109 (12.5%) | 66 (3.8%) |
| Portugal | 0 (0%) | 13 (0.7%) |

Shown are numbers and percentages of samples that did not pass quality control. The variability of the data around the mean is expressed as the standard deviation (SD).

**Supplementary Figure 1.** **Explanation (non-)consistent reads.** Read alignment created with REViewer of *ATXN8*, which has 2 consecutive STRs, namely CTA (orange part of the alignment) and CTG (green part of the alignment). The blue parts of the alignment are the flanking sequences. Blue arrows show consistent reads and orange arrows show non-consistent reads. According to ExpansionHunter this individual has one allele with 11 CTA repeat units and 13 CTG repeat units (A, allele 1). Evidence for this comes from 12 reads which have the same number of repeat units as ExpansionHunter and are therefore called ‘consistent’ (blue arrows). There are, however, 10 reads that support 9 CTA repeat units and 6 CTG repeat units, and 5 reads that support 10 CTA repeat units and 9 CTG repeat units, and which are therefore called ‘non-consistent’ (orange arrows). The reads without arrows support multiple CTA and/or CTG allele sizes. The non-consistent reads show that more than 2 alleles exist in this sample. There are less consistent than non-consistent reads (LCTNC). The other chromosome of this individual (B, allele 2) has more than 11 CTA repeat units (evidence in black rectangle), though according to ExpansionHunter it has 9 CTA repeat units, and approximately 62 CTG repeat units. The latter is based on 6 in-repeat reads (green-only reads). Evidence from in-repeat reads is considered first by ExpansionHunter. If there is evidence for more than two alleles, ExpansionHunter chooses the alleles with the highest number of consistent reads, in this case 11 CTA and 13 CTG repeat units.


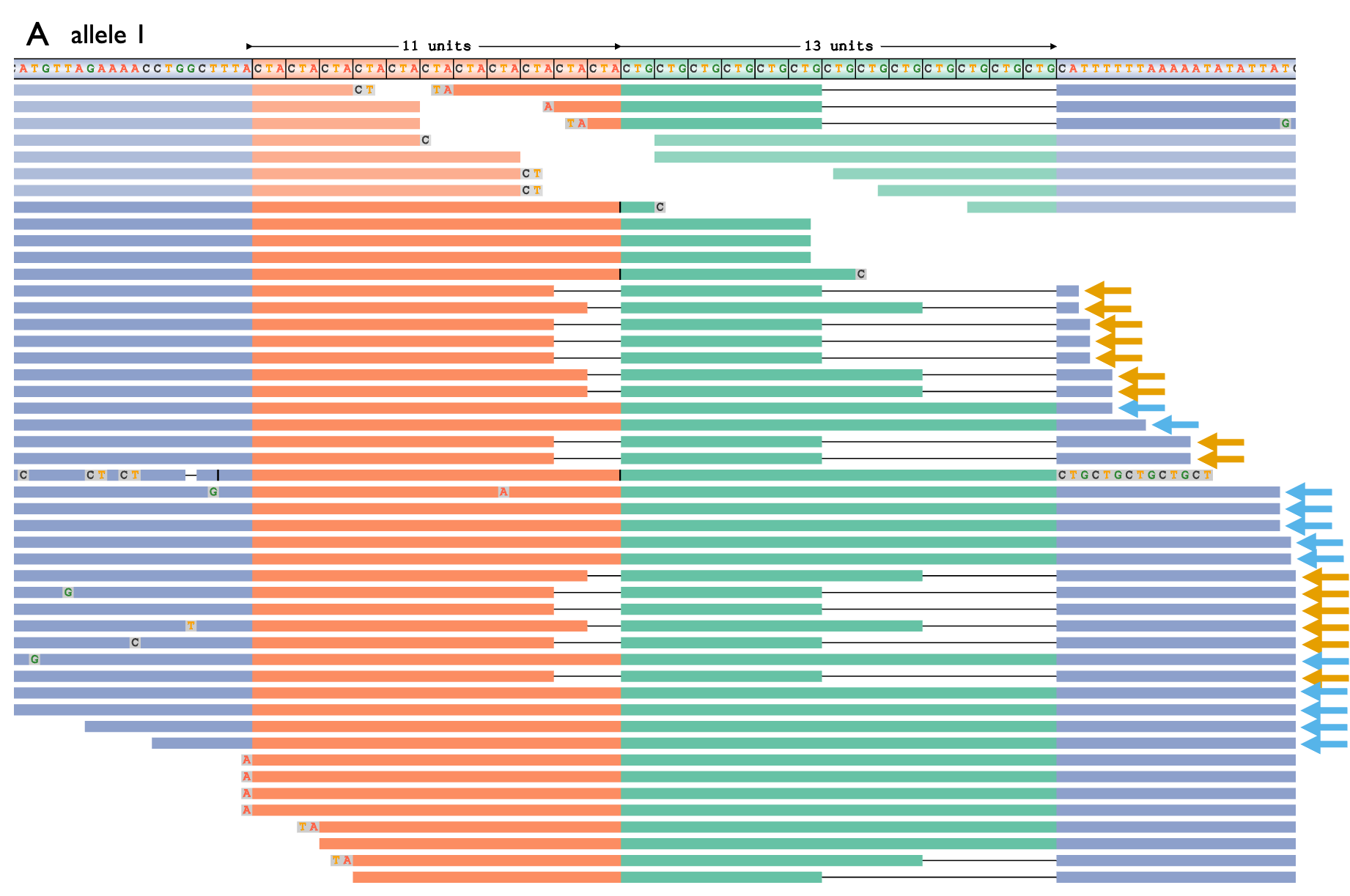


**
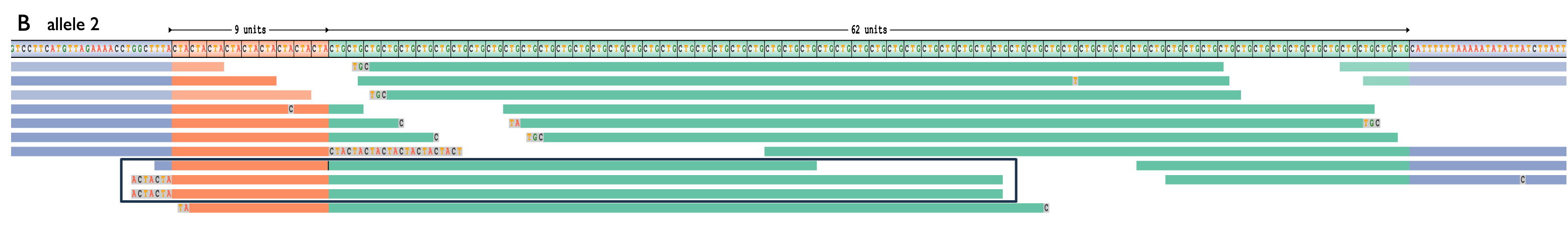
**

**Supplementary Table 5.** **Sample counts analyzed per STR, sequencing platform and repeat size determination technique.**

|  | **HiSeq2000** | | | | **HiSeqX** | | | |
| --- | --- | --- | --- | --- | --- | --- | --- | --- |
| **RepeatID** | **PCR1** | **PCR2** | **PCR3** | **Sanger** | **PCR1** | **PCR2** | **PCR3** | **Sanger** |
| *ATXN1* | 1101 |  |  | 718 | 475 |  |  |  |
| *ATXN2* | 777 |  |  |  | 271 |  |  |  |
| *NIPA1* | 325 | 322 | 507 | 585 | 240 | 173 | 3 | 687 |

**Supplementary Figure 2.** **Difference in genotyped repeat size between ExpansionHunter and PCR.** Analyzed from *ATXN1*, *ATXN2* and *NIPA1* STR results in Table 1, grouped by sequencing platform (HiSeq2000 or HiSeqX) and ExpansionHunter version (EHv3 or EHv5). The values below each boxplot represent the allele count per group.


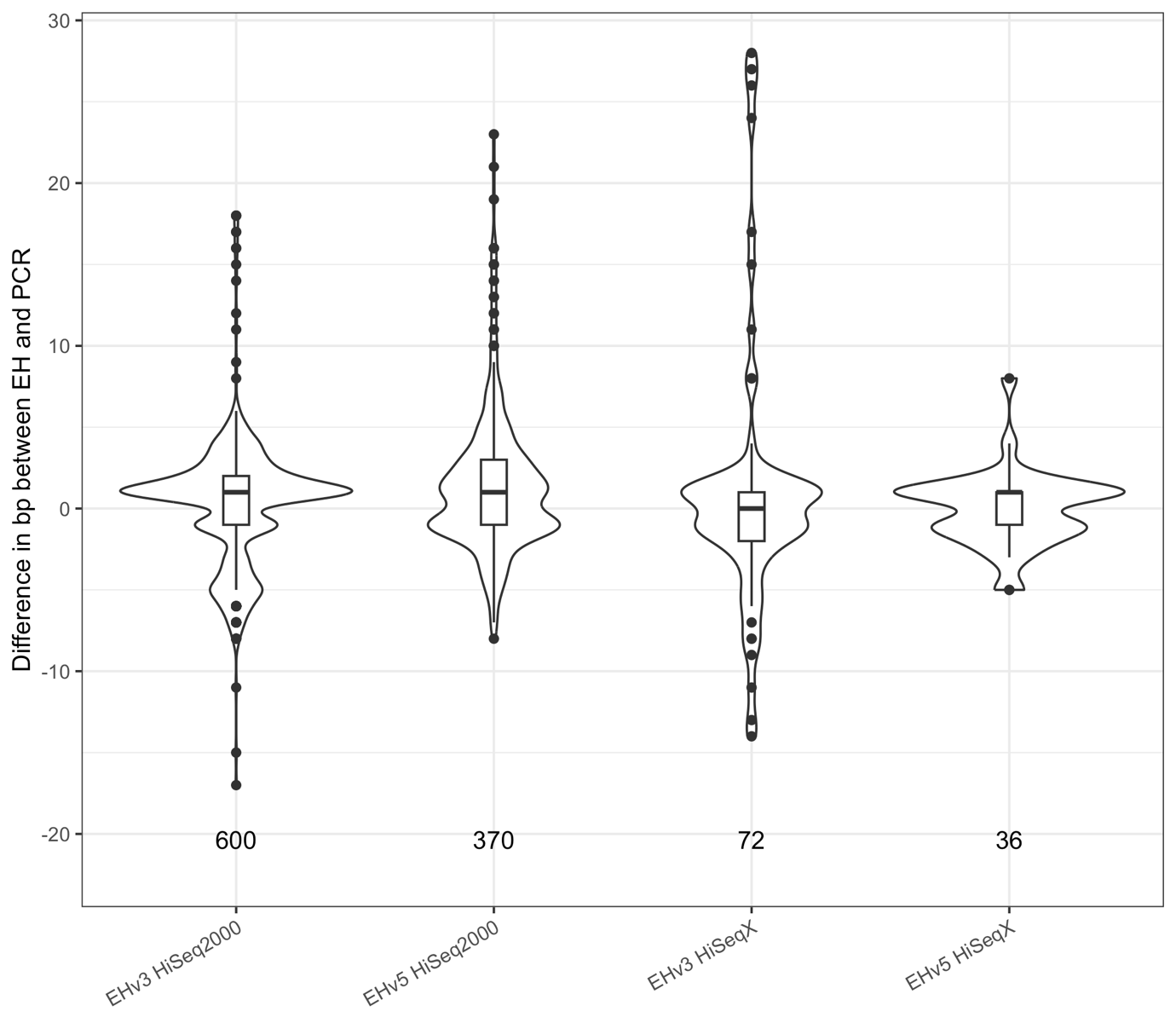


**Supplementary Table 6.** **Genotyping discordance between ExpansionHunter and PCR.**

| **Group** | **Count** | **Mean** | **SD** | **Var** |
| --- | --- | --- | --- | --- |
| ExpansionHunter version 3 HiSeq2000 | 600 | 0.30 | 3.8 | 15 |
| ExpansionHunter version 3 HiSeqX | 72 | 0.93 | 7.9 | 63 |
| ExpansionHunter version 5 HiSeq2000 | 370 | 1.3 | 4.3 | 18 |
| ExpansionHunter version 5 HiSeqX | 36 | -0.056 | 2.4 | 5.6 |

Count, mean, standard deviation (SD) and variance (Var) in base pair (bp) difference between ExpansionHunter and PCR, analyzed from *ATXN1*, *ATXN2* and *NIPA1* STR results in Table 1.

**Supplementary Table 7. Significance of genotyping discordance between ExpansionHunter and PCR.**

| **Groups** | **Pcount** | **Pvar** |
| --- | --- | --- |
| ExpansionHunter version 3 HiSeq2000 vs ExpansionHunter version 3 HiSeqX | 5.9E-19 | 2.6E-06 |
| ExpansionHunter version 5 HiSeq2000 vs ExpansionHunter version5 HiSeqX | 1.4E-10 | 0.031 |
| ExpansionHunter version 3 HiSeq2000 vs ExpansionHunter version 5 HiSeq2000 | 3.2E-14 | 0.038 |
| ExpansionHunter version 3 HiSeqX vs ExpansionHunter version 5 HiSeqX | 0.030 | 0.012 |

Analyzed from *ATXN1*, *ATXN2* and *NIPA1* STR results in Table 1. Non-parametric test of count was performed with Chi-square (P_count_). Non-parametric test of variance was performed with Levene (P_var_).

**Supplementary Table 8. Agreement in genotype assessment of REViewer plots.**

| **Test** | **Score** |
| --- | --- |
| Intraclass Correlation Coefficient | 0.989 |
| Cohen’s kappa | 0.943 |
| Kendall's W (uncorrected for ties) | 0.993 |
| Kendall's W (corrected for ties) | 0.996 |
| Spearman's ranked correlation | 0.993 |

**Supplementary Figure 3. Genotyping accuracy from binary repeat parameters.** A) Frequency of alleles with a binary repeat parameter per STR. B) Failed genotyping frequency per STR grouped by the number of binary repeat parameters. Each dot is an STR, not all repeat parameters have alleles of each STR. The failed genotyping frequency was compared between the groups with 0 and 1 repeat parameter (n = 29 and 35, respectively) and between the groups with 1 and more than 1 repeat parameter (n = 35 and 17, respectively) by means of a t-test.


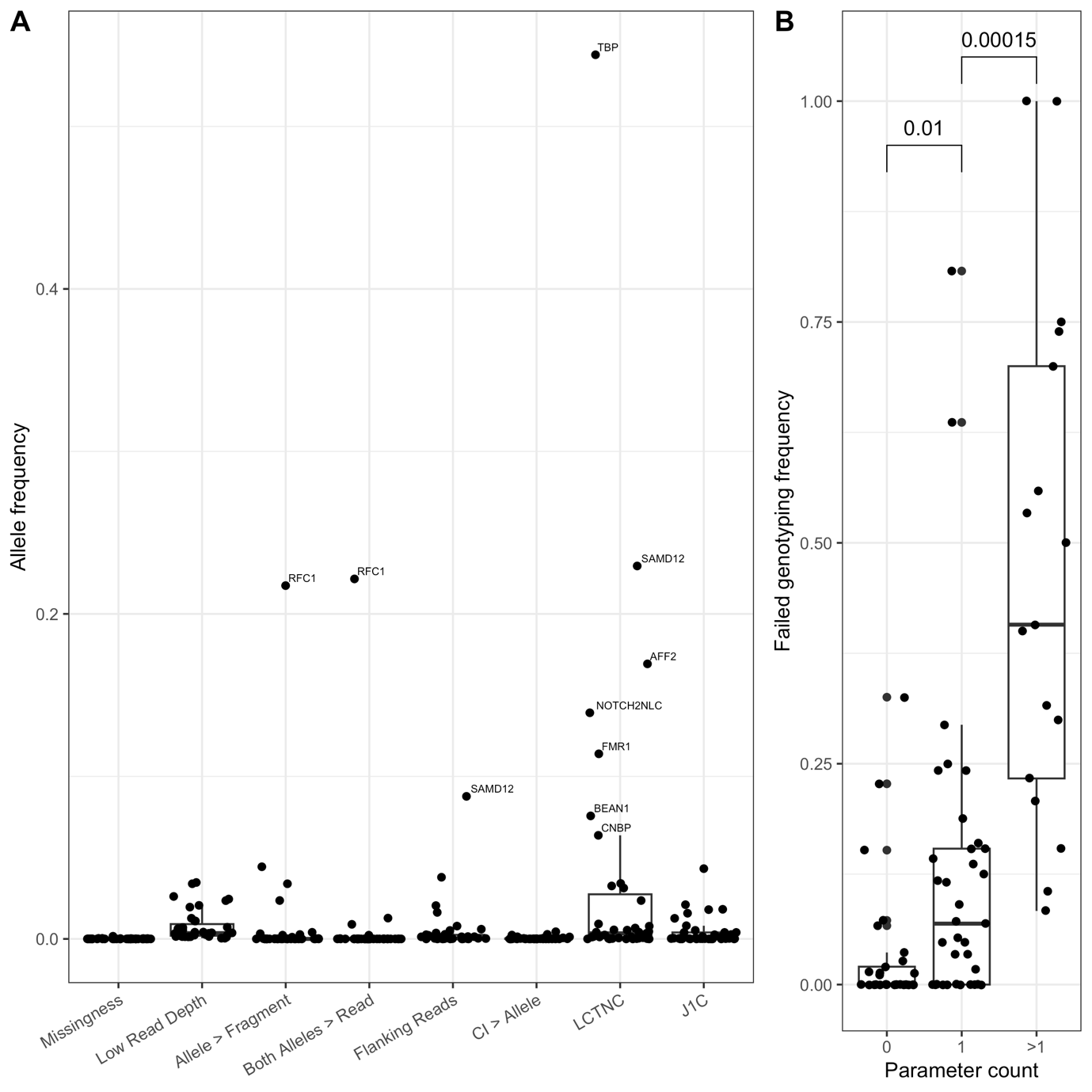


**Supplementary Figure 4. Predicted genotyping accuracy in test set.** Result of genotyping accuracy prediction model on the test set (n = 850), which was built using a generalized linear model in spanning reads (A), flanking reads (B) and in-repeat reads (C). In the left panel the average value per STR is shown of alleles that failed and passed genotyping assessment as predicted by the model. In the right panel the overall sensitivity and specificity is compared to the value predicted by the model. AUC means Area Under the Curve.


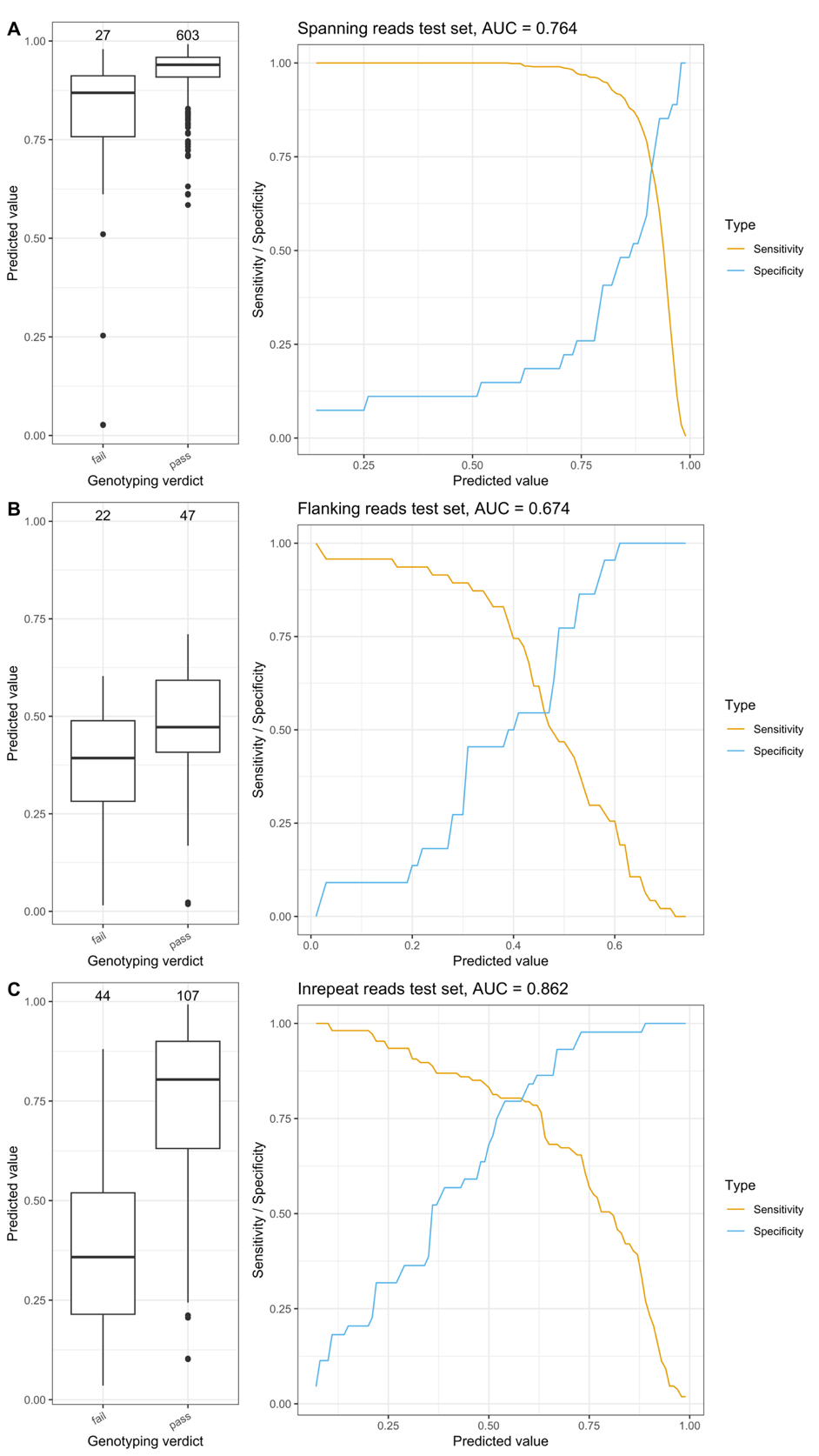


**Supplementary Figure 5. Sensitivity and specificity per STR.** The sensitivity and specificity of genotyping accuracy prediction across the disease-associated STRs based on the generalized linear model. See Supplementary Fig. 4 for statistical details of this prediction model.

**
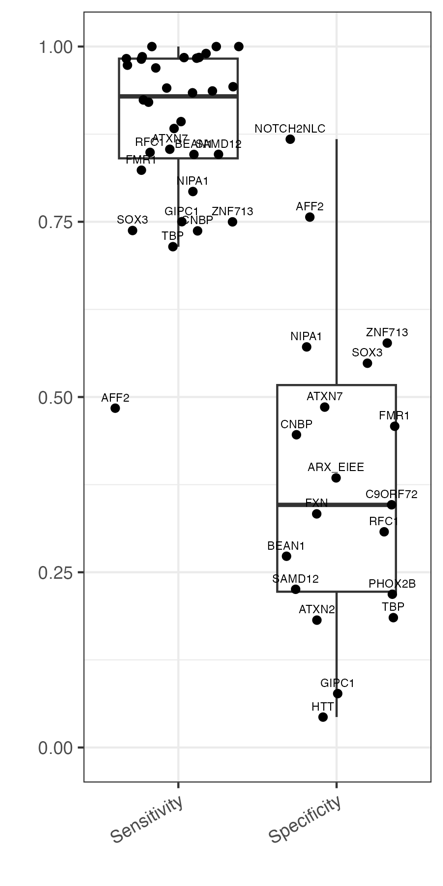
**


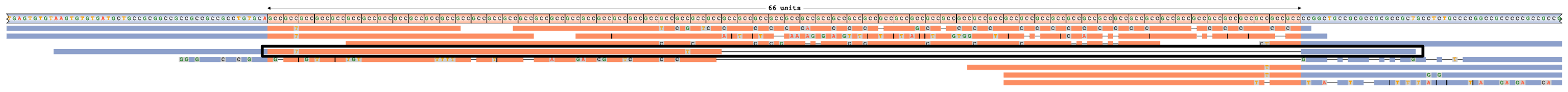
**Supplementary Figure 6.** **REViewer example *AFF2*.** Read-aligned plot of the *AFF2* STR from a man with 66 repeat units according to ExpansionHunter. The reads considered as evidence for 66 repeat units have a poor alignment to the reference. The spanning read in the black rectangle has 29 repeat units, which is most likely the true genotype of this individual.

**Supplementary Table 9.** **ALS susceptibility association of binary repeat parameters.**

| **RepeatID** | **RepeatParameter** | **Cases (%)** | **Controls (%)** | **OR** | **95% CI** | **P** | **Pbon** |
| --- | --- | --- | --- | --- | --- | --- | --- |
| *C9ORF72* | FragmentLengthLimited | 325 (3.1) | 7 (0.20) | 17 | 8.80 - 38 | < 2.2E-16 | < 2.2E-16 |
| *C9ORF72* | OffAllelicDepth | 433 (4.1) | 44 (1.3) | 3.60 | 2.70 - 5.10 | < 2.2E-16 | < 2.2E-16 |
| *C9ORF72* | J1C | 54 (0.51) | 2 (0.057) | 8.50 | 2.90 - 41 | 5.8E-06 | 6.0E-04 |
| *C9ORF72* | LCTNC | 68 (0.65) | 8 (0.23) | 2.70 | 1.40 - 6.00 | 2.5E-03 | 0.20 |
| *AR* | LCTNC | 54 (0.73) | 38 (1.5) | 0.54 | 0.35 - 0.83 | 5.2E-03 | 0.32 |
| *NOP56* | Missingness | 7 (0.13) | 9 (0.52) | 0.25 | 0.090 - 0.67 | 6.5E-03 | 0.18 |
| *AR* | OffAllelicDepth | 11 (0.15) | 12 (0.46) | 0.37 | 0.16 - 0.86 | 0.022 | 0.68 |
| *CSTB* | FragmentLengthLimited | 15 (0.14) | 0 (0) | 9.80 | 1.30 - 1300 | 0.022 | 0.68 |
| *ATN1* | Flanking | 0 (0) | 2 (0.057) | 0.053 | 0.00038 - 0.68 | 0.023 | 0.68 |
| *DAB1* | LCTNC | 376 (3.6) | 104 (3.0) | 1.30 | 1.00 - 1.60 | 0.023 | 0.68 |
| *ATN1* | J1C | 0 (0) | 2 (0.057) | 0.057 | 0.00042 - 0.71 | 0.025 | 0.68 |
| *ZNF713* | LCTNC | 238 (2.3) | 94 (2.7) | 0.75 | 0.59 - 0.96 | 0.025 | 0.68 |
| *AFF2* | OffAllelicDepth | 188 (2.5) | 57 (2.2) | 1.40 | 1.00 - 1.90 | 0.026 | 0.68 |
| *ATXN10* | OffAllelicDepth | 18 (0.17) | 1 (0.028) | 4.60 | 1.10 - 42 | 0.030 | 0.71 |
| *C9ORF72* | RatioRepeatNrCI | 16 (0.15) | 1 (0.028) | 4.60 | 1.10 - 43 | 0.032 | 0.71 |
| *C9ORF72* | Flanking | 62 (0.59) | 11 (0.31) | 1.90 | 1.00 - 3.80 | 0.035 | 0.72 |
| *AFF2* | LCTNC | 1235 (17) | 456 (17) | 1.10 | 1.00 - 1.30 | 0.040 | 0.79 |
| *ATXN7* | LCTNC | 30 (0.28) | 18 (0.51) | 0.53 | 0.30 - 0.98 | 0.043 | 0.79 |
| *ATXN3* | OffAllelicDepth | 28 (0.27) | 3 (0.085) | 2.70 | 1.00 - 10 | 0.049 | 0.85 |

ALS association analysis of disease-associated STRs with binary repeat parameters. The binary repeat parameters involved are 1) alleles set to missing by means of the genotyping accuracy model (Missingness), 2) alleles called from flanking reads (Flanking), 3) both alleles called from in-repeat reads (OnlyIRR), 4) alleles that are potentially longer than the fragment length (FragmentLengthLimited), 5) alleles of which the allelic read depth is five times higher or lower than the average read depth (OffAllelicDepth), 6) alleles of which the repeat size confidence interval is larger than the repeat size (RatioRepeatNrCI), 7) alleles called from a single consistent read (J1C), and 8) alleles with less consistent than non-consistent reads (LCTNC). P is the uncorrected p-value and P_bon_ is the p-value Bonferroni corrected for the number of STRs tested and parameters tested per STR. ‘95% CI’ is the 95% confidence interval of the odds ratio (OR). Results with a nominally significant p-value are included.

**Supplementary Figure 7. REViewer example *C9orf72*.** Read-aligned plot of *C9orf72* from an individual with 24 and > 500 repeat units according to ExpansionHunter. The allele with 24 repeat units (allele 1) has 5 repeats based on the spanning reads in the black rectangle. Because there are much more (left) flanking reads than spanning reads, ExpansionHunter mistakenly uses flanking reads to determine the repeat size of this allele.

**
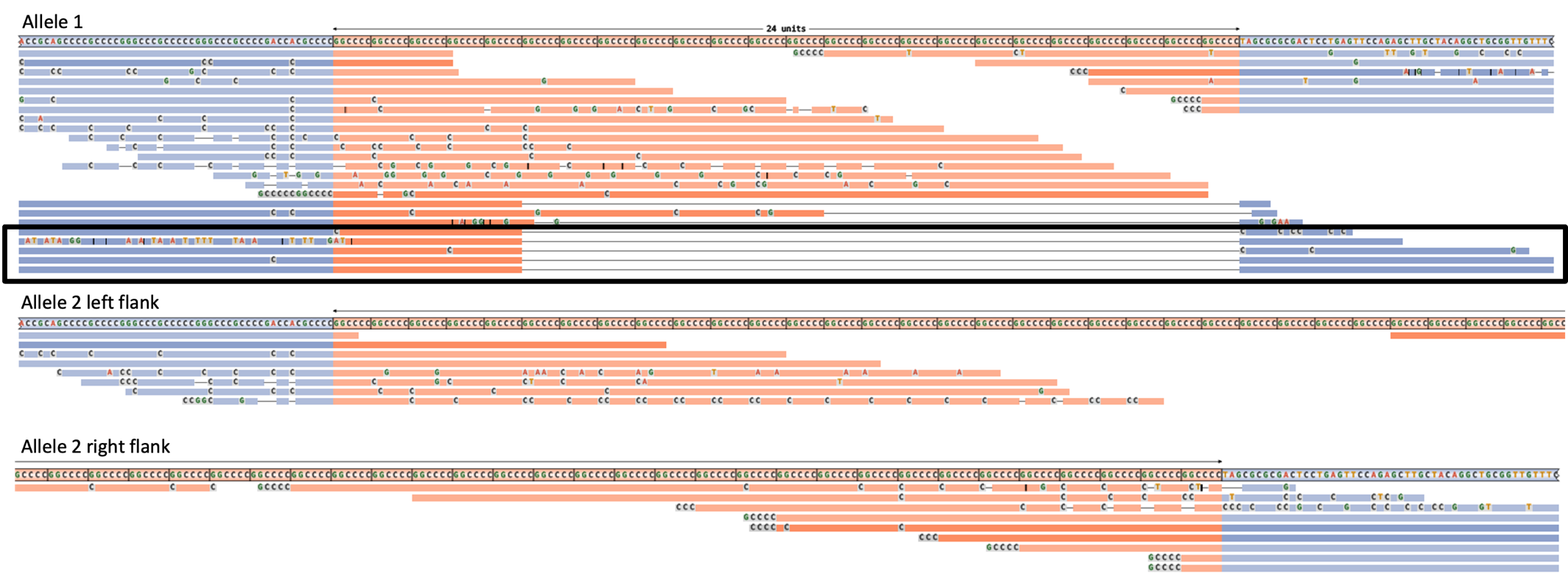
**

**Supplementary Table 10.** **ALS susceptibility association of *C9orf72* pathogenic and premutated individuals before and after genotyping correction.**

| **RepeatID** | **Type** | **Thresholds** | **Cases (%)** | **Controls (%)** | **P** | **OR** | **95% CI** |
| --- | --- | --- | --- | --- | --- | --- | --- |
| *C9ORF72* | pathogenic original | >=30 | 336 (6.4) | 8 (0.46) | < 2.2E-16 | 16 | 8.6 - 34 |
| *C9ORF72* | pathogenic corrected | >=30 | 333 (6.5) | 8 (0.47) | < 2.2E-16 | 16 | 8.5 - 34 |
| *C9ORF72* | premutation original | >=24-30 | 9 (0.17) | 2 (0.11) | 0.56 | 1.5 | 0.41 - 8.1 |
| *C9ORF72* | premutation corrected | >=24-30 | 6 (0.12) | 2 (0.12) | 0.82 | 1.2 | 0.29 - 6.7 |

Before genotyping correction 18 out of the 336 pathogenic ALS patients had an expanded and intermediate allele, whereas after correction no individuals had an expanded and intermediate allele. P is the uncorrected p-value. ‘95% CI’ is the 95% confidence interval of the odds ratio (OR).

**Supplementary Figure 8. Repeat size distributions before and after genotyping correction.** The number of repeat units per allele is compared to the square root of the frequency for each of the 39 disease-associated STRs in all Project MinE samples (n = 6,983). For an improved interpretability of the plot the frequency of alleles with more than 90 repeat units were summed.


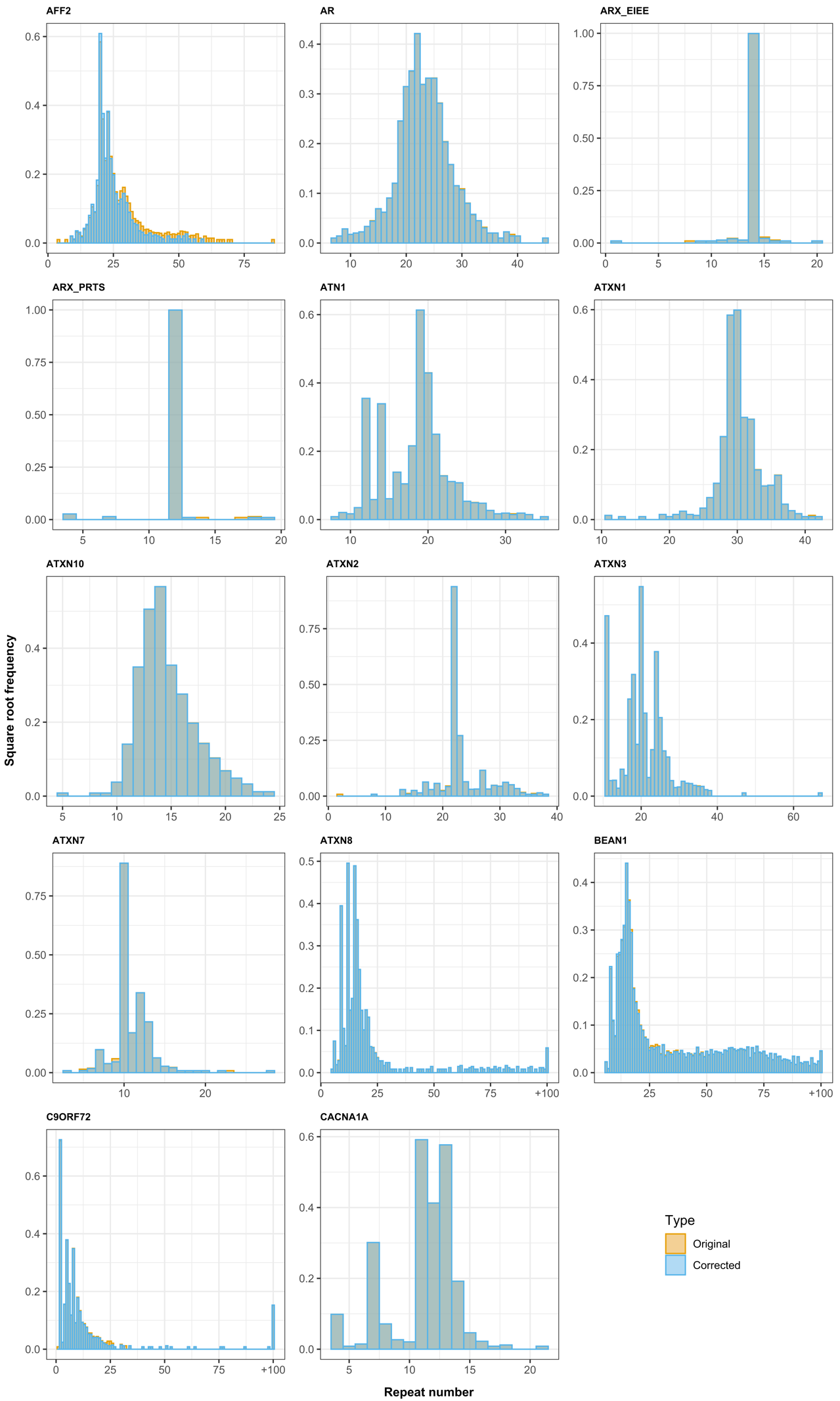

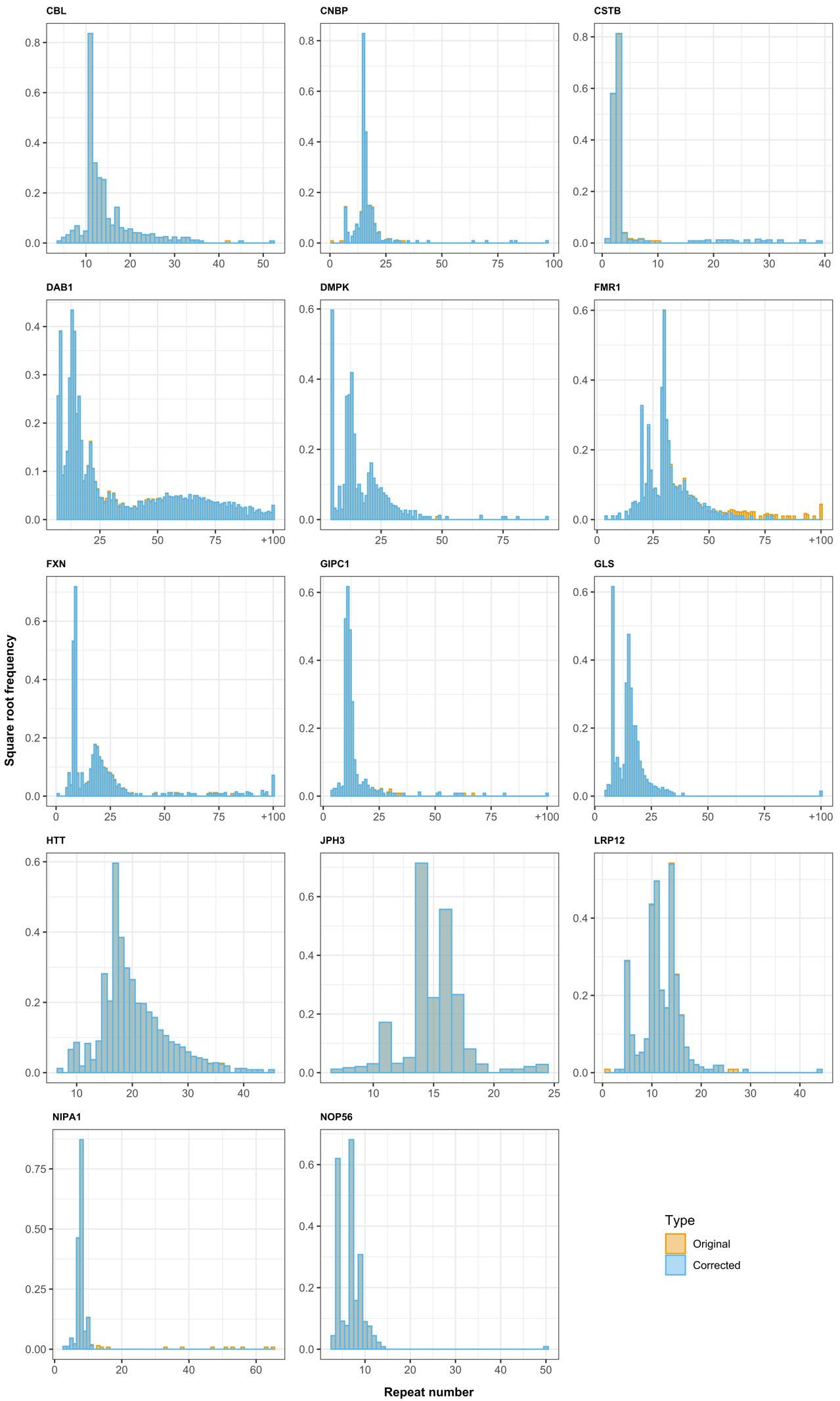

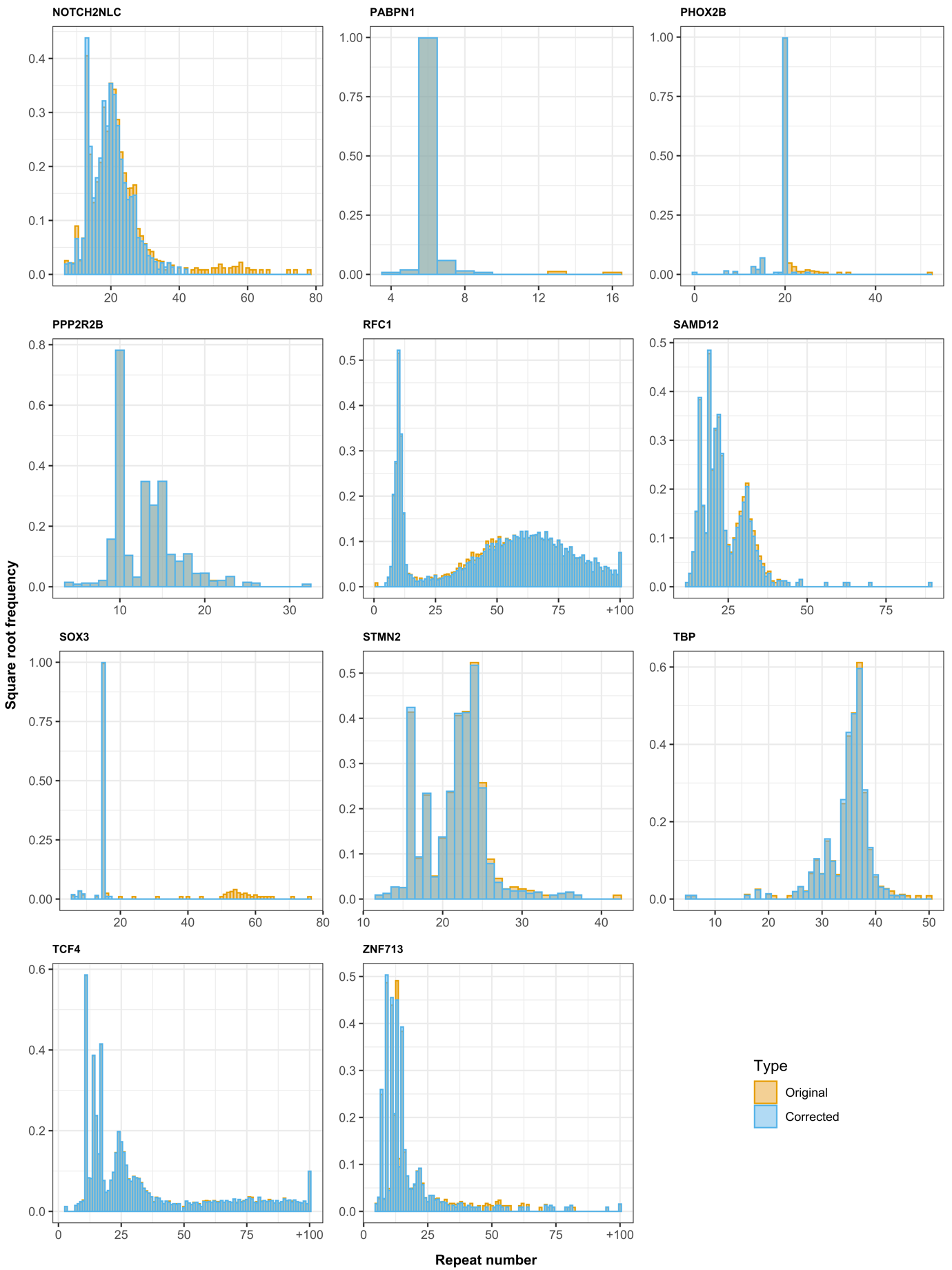


**Supplementary Figure 9. Repeat size distributions of patients and controls after genotyping correction.** The number of repeat units per allele after genotyping correction is compared to the square root of the frequency for each of the 28 disease-associated STRs with sufficient genotyping accuracy in the 5,237 cases and 1,746 controls of Project MinE. For an improved interpretability of the plot the frequency of alleles with more than 90 repeat units were summed.


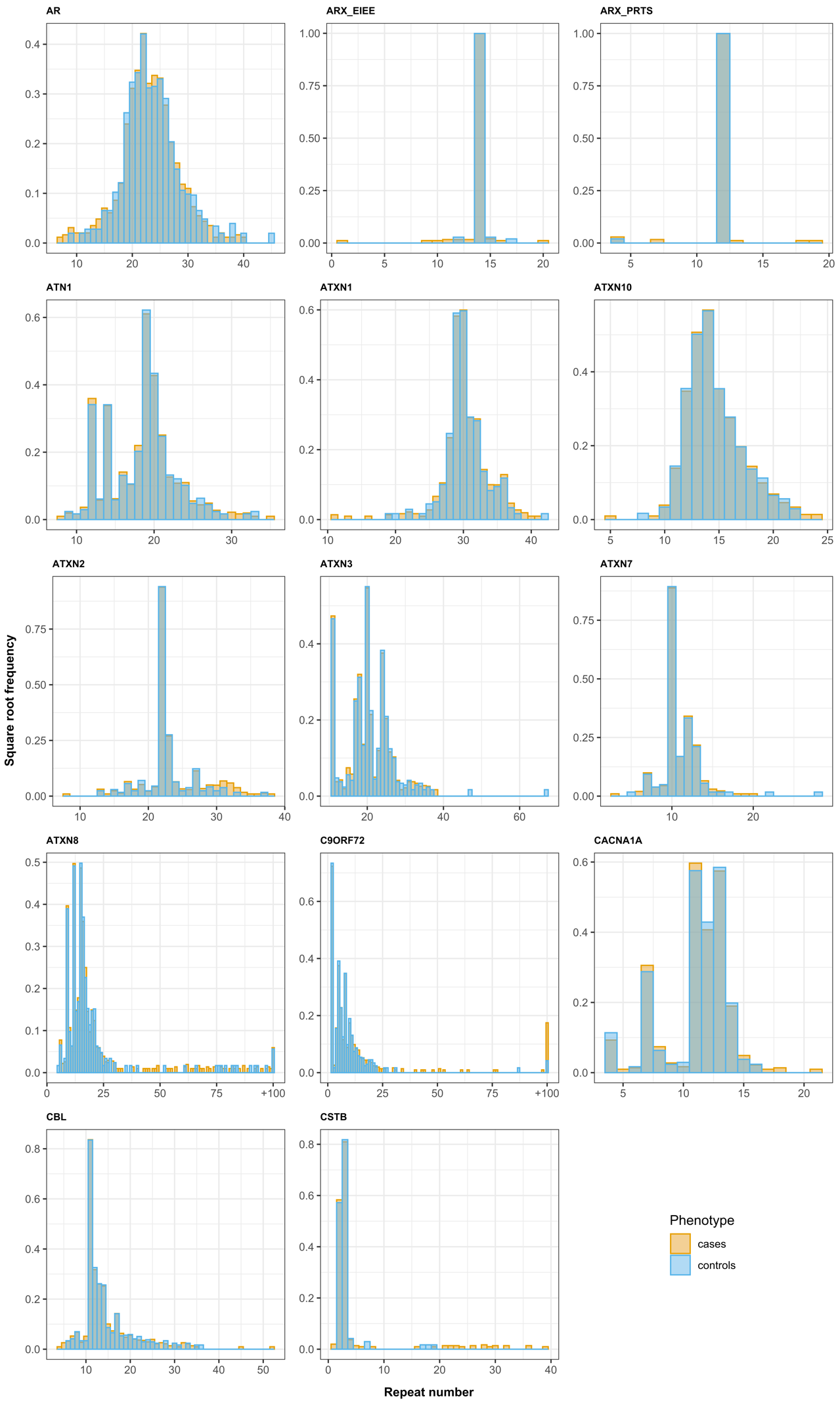

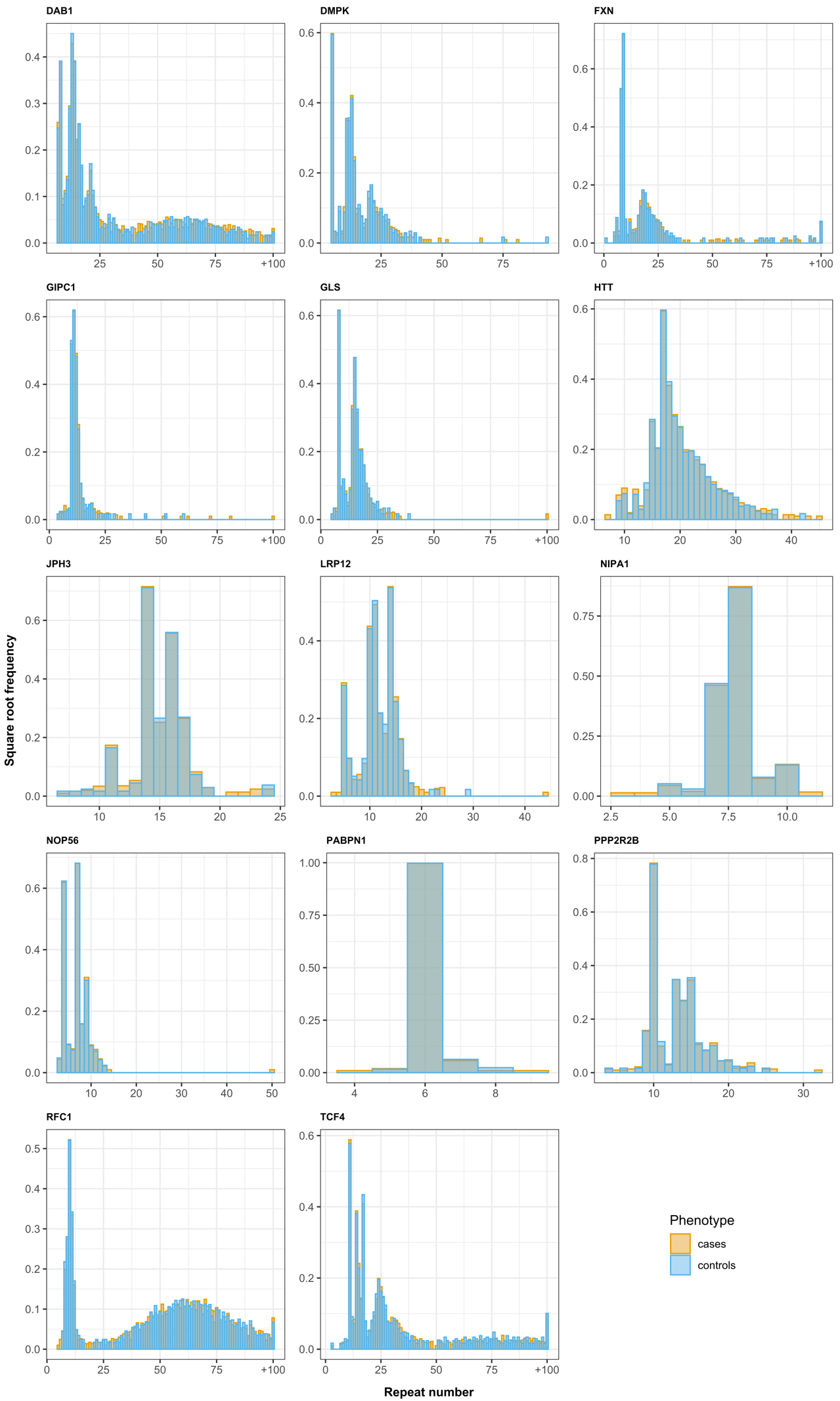


**Supplementary Table 11. Number of pathogenic and premutation carriers and significance with ALS susceptibility.**

| **RepeatID** | **Disease** | **Inher** | **Type** | **Threshold** | **Cases (%)** | **Controls (%)** | **P** | **Pbon** | **OR** | **95% CI** |
| --- | --- | --- | --- | --- | --- | --- | --- | --- | --- | --- |
| *AR* | SBMA | XR | pathogenic | 38 | 1 (0.019) | 1 (0.058) | 0.57 | 1.00 | 0.51 | 0.041 - 6.37 |
|  |  |  | premutation | 35 | 3 (0.058) | 1 (0.058) | 0.65 | 1.00 | 0.62 | 0.096 - 6.73 |
| *ATXN1* | SCA1 | AD | pathogenic | 39 | 6 (0.12) | 1 (0.058) | 0.64 | 1.00 | 1.52 | 0.30 - 15 |
|  |  |  | premutation | 33 | 605 (12) | 170 (9.8) | 6.9E-03 | 0.33 | 1.28 | 1.07 - 1.55 |
| *ATXN2* | SCA2 | AD | pathogenic | 33 | 23 (0.45) | 2 (0.12) | 0.046 | 1.00 | 3.21 | 1.02 - 16 |
|  |  |  | premutation | 29 | 129 (2.5) | 13 (0.76) | 1.4E-05 | 6.6E-04 | 3.02 | 1.77 - 5.60 |
| *ATXN3* | SCA3 | AD | pathogenic | 60 | 0 (0) | 1 (0.057) | 0.078 | 1.00 | 0.071 | 0.00048 - 1.36 |
|  |  |  | premutation | 45 | 0 (0) | 1 (0.057) | 0.42 | 1.00 | 0.29 | 0.0020 - 5.52 |
| *ATXN8* | SCA8 | AD | pathogenic | 80 | 55 (1.1) | 18 (1.0) | 0.84 | 1.00 | 0.95 | 0.56 - 1.67 |
|  |  |  | premutation | 51 | 28 (0.54) | 7 (0.40) | 0.70 | 1.00 | 1.17 | 0.54 - 2.87 |
| *C9ORF72* | ALS/FTD | AD | pathogenic | 30 | 333 (6.5) | 8 (0.47) | < 2.2E-16 | < 2.2E-16 | 16 | 8.51 - 34 |
|  |  |  | premutation | 24 | 6 (0.12) | 2 (0.12) | 0.82 | 1.00 | 1.19 | 0.29 - 6.66 |
| *CACNA1A* | SCA6 | AD | pathogenic | 20 | 1 (0.019) | 0 (0) | 0.60 | 1.00 | 0.39 | 0.020 - 57 |
|  |  |  | premutation | 19 | 0 (0) | 0 (0) | 1.00 | 1.00 | 1.00 | 1.00 - 1.00 |
| *CBL* | FRA11B | AD | pathogenic | 100 | 0 (0) | 0 (0) | 1.00 | 1.00 | 1.00 | 1.00 - 1.00 |
| *DMPK* | DM1 | AD | pathogenic | 50 | 5 (0.096) | 2 (0.11) | 0.58 | 1.00 | 0.64 | 0.14 - 3.69 |
|  |  |  | premutation | 35 | 29 (0.55) | 12 (0.69) | 0.61 | 1.00 | 0.83 | 0.43 - 1.71 |
| *GIPC1* | OPDM2 | AD | pathogenic | 73 | 2 (0.039) | 0 (0) | 0.84 | 1.00 | 1.36 | 0.11 - 188 |
|  |  |  | premutation | 32 | 7 (0.14) | 4 (0.23) | 0.33 | 1.00 | 0.53 | 0.16 - 1.97 |
| *HTT* | HD | AD | pathogenic | 40 | 5 (0.096) | 1 (0.058) | 0.86 | 1.00 | 1.17 | 0.22 - 12 |
|  |  |  | premutation | 27 | 316 (6.1) | 105 (6.1) | 0.95 | 1.00 | 1.01 | 0.80 - 1.28 |
| *NIPA1* | HSP6 | AD | pathogenic | 9 | 232 (4.6) | 78 (4.6) | 0.91 | 1.00 | 1.02 | 0.78 - 1.34 |
| *NOP56* | SCA36 | AD | pathogenic | 650 | 1 (0.019) | 0 (0) | 0.95 | 1.00 | 1.10 | 0.058 - 161 |
| *PABPN1* | OPMD | AD | pathogenic | 8 | 2 (0.038) | 2 (0.11) | 0.15 | 1.00 | 0.25 | 0.036 - 1.72 |
| *RFC1* | CANVAS | AR | pathogenic | 400 | 309 (6.0) | 96 (5.6) | 0.28 | 1.00 | 1.14 | 0.90 - 1.46 |
| *TCF4* | FECD3 | AD | pathogenic | 80 | 228 (4.4) | 77 (4.5) | 0.88 | 1.00 | 1.02 | 0.78 - 1.35 |
|  |  |  | premutation | 41 | 200 (3.9) | 79 (4.6) | 0.25 | 1.00 | 0.85 | 0.65 - 1.12 |

These numbers were based on literature threshold, disease-associated inheritance and repeat sizes after genotyping correction. Alleles limited by the fragment length were deemed pathogenic. Premutation p-values were based on the number of premutation carriers (not premutation and pathogenic carriers). “Inher” denotes disease-associated inheritance. XR: X-linked recessive, AR: Autosomal recessive, AD: autosomal dominant. P is the uncorrected p-value and P_bon_ is the p-value Bonferroni corrected for the number of STRs and thresholds tested per STR. ‘95% CI’ is the 95% confidence interval of the odds ratio (OR). STRs without carriers were not included.

**Supplementary Table 12. Number of pathogenic and premutation carriers without disease-related inheritance and significance with ALS susceptibility.**

| **RepeatID** | **Disease** | **Type** | **Threshold** | **Cases 2 (%)** | **Cases 1 (%)** | **Controls 2 (%)** | **Controls 1 (%)** | **P** | **Pbon** | **OR** | **95% CI** |
| --- | --- | --- | --- | --- | --- | --- | --- | --- | --- | --- | --- |
| *AR* | SBMA | pathogenic | 38 | 0 (0) | 4 (0.077) | 0 (0) | 6 (0.35) | 0.063 | 1.00 | 0.32 | 0.087 - 1.07 |
|  |  | premutation | 35 | 0 (0) | 5 (0.096) | 0 (0) | 4 (0.23) | 0.17 | 1.00 | 0.40 | 0.11 - 1.53 |
| *ARX_EIEE* | EIEE | pathogenic | 17 | 0 (0) | 1 (0.019) | 0 (0) | 1 (0.058) | 0.32 | 1.00 | 0.31 | 0.025 - 3.81 |
| *ATXN1* | SCA1 | pathogenic | 39 | 0 (0) | 5 (0.096) | 0 (0) | 1 (0.058) | 0.84 | 1.00 | 1.21 | 0.22 - 12 |
|  |  | premutation | 33 | 19 (0.36) | 586 (11) | 4 (0.25) | 166 (11) | 6.2E-03 | 0.30 | 1.28 | 1.07 - 1.53 |
| *ATXN2* | SCA2 | pathogenic | 33 | 0 (0) | 23 (0.45) | 0 (0) | 2 (0.12) | 0.047 | 1.00 | 3.20 | 1.01 - 16 |
|  |  | premutation | 29 | 4 (0.078) | 125 (2.4) | 0 (0) | 13 (0.76) | 1.1E-05 | 5.5E-04 | 2.98 | 1.76 - 5.49 |
| *ATXN3* | SCA3 | pathogenic | 60 | 0 (0) | 0 (0) | 0 (0) | 1 (0.057) | 0.078 | 1.00 | 0.071 | 0.00048 - 1.36 |
|  |  | premutation | 45 | 0 (0) | 0 (0) | 0 (0) | 1 (0.057) | 0.42 | 1.00 | 0.29 | 0.0020 - 5.52 |
| *ATXN8* | SCA8 | pathogenic | 80 | 0 (0) | 55 (1.1) | 0 (0) | 18 (1.0) | 0.84 | 1.00 | 0.94 | 0.56 - 1.67 |
|  |  | premutation | 51 | 0 (0) | 28 (0.54) | 0 (0) | 7 (0.40) | 0.70 | 1.00 | 1.17 | 0.54 - 2.87 |
| *C9ORF72* | ALS/FTD | pathogenic | 30 | 0 (0) | 333 (6.5) | 0 (0) | 8 (0.47) | < 2.2E-16 | < 2.2E-16 | 16 | 8.50 - 34 |
|  |  | premutation | 24 | 0 (0) | 6 (0.12) | 0 (0) | 2 (0.12) | 0.81 | 1.00 | 1.21 | 0.29 - 6.76 |
| *CACNA1A* | SCA6 | pathogenic | 20 | 0 (0) | 1 (0.019) | 0 (0) | 0 (0) | 0.60 | 1.00 | 0.39 | 0.020 - 58 |
|  |  | premutation | 19 | 0 (0) | 0 (0) | 0 (0) | 0 (0) | 1.00 | 1.00 | 1.00 | 1.00 - 1.00 |
| *CSTB* | ULD | pathogenic | 30 | 0 (0) | 15 (0.29) | 0 (0) | 0 (0) | 0.021 | 1.00 | 9.93 | 1.30 - 1274 |
|  |  | premutation | 4 | 0 (0) | 25 (0.49) | 0 (0) | 12 (0.71) | 0.25 | 1.00 | 0.66 | 0.34 - 1.36 |
| *DMPK* | DM1 | pathogenic | 50 | 0 (0) | 5 (0.096) | 0 (0) | 2 (0.11) | 0.58 | 1.00 | 0.64 | 0.14 - 3.69 |
|  |  | premutation | 35 | 0 (0) | 29 (0.55) | 0 (0) | 12 (0.69) | 0.61 | 1.00 | 0.84 | 0.43 - 1.71 |
| *FXN* | FRDA | pathogenic | 66 | 0 (0) | 71 (1.4) | 0 (0) | 30 (1.8) | 0.45 | 1.00 | 0.84 | 0.55 - 1.33 |
|  |  | premutation | 35 | 0 (0) | 16 (0.32) | 0 (0) | 4 (0.24) | 0.66 | 1.00 | 1.27 | 0.47 - 4.19 |
| *GIPC1* | OPDM2 | pathogenic | 73 | 0 (0) | 2 (0.039) | 0 (0) | 0 (0) | 0.84 | 1.00 | 1.35 | 0.11 - 187 |
|  |  | premutation | 32 | 0 (0) | 7 (0.14) | 0 (0) | 4 (0.23) | 0.33 | 1.00 | 0.53 | 0.16 - 1.97 |
| *GLS* | GD | pathogenic | 600 | 0 (0) | 0 (0) | 0 (0) | 0 (0) | 1.00 | 1.00 | 1.00 | 1.00 - 1.00 |
|  |  | premutation | 38 | 0 (0) | 3 (0.057) | 0 (0) | 1 (0.057) | 1.00 | 1.00 | 1.00 | 0.16 - 10 |
| *HTT* | HD | pathogenic | 40 | 0 (0) | 5 (0.096) | 0 (0) | 1 (0.058) | 0.86 | 1.00 | 1.18 | 0.22 - 12 |
|  |  | premutation | 27 | 4 (0.077) | 312 (6.0) | 2 (0.12) | 103 (6.3) | 0.99 | 1.00 | 1.00 | 0.80 - 1.26 |
| *NIPA1* | HSP6 | pathogenic | 9 | 5 (0.098) | 227 (4.4) | 0 (0) | 78 (4.8) | 0.85 | 1.00 | 1.03 | 0.79 - 1.35 |
| *NOP56* | SCA36 | pathogenic | 650 | 0 (0) | 1 (0.019) | 0 (0) | 0 (0) | 0.97 | 1.00 | 1.07 | 0.056 - 157 |
| *PABPN1* | OPMD | pathogenic | 8 | 0 (0) | 2 (0.038) | 0 (0) | 2 (0.12) | 0.15 | 1.00 | 0.25 | 0.036 - 1.73 |
| *RFC1* | CANVAS | pathogenic | 400 | 309 (6.2) | 1619 (33) | 96 (8.4) | 526 (46) | 0.080 | 1.00 | 1.09 | 0.99 - 1.20 |
| *TCF4* | FECD3 | pathogenic | 80 | 8 (0.15) | 220 (4.3) | 3 (0.18) | 74 (4.5) | 0.87 | 1.00 | 1.02 | 0.79 - 1.33 |
|  |  | premutation | 41 | 3 (0.058) | 197 (3.8) | 0 (0) | 79 (4.8) | 0.33 | 1.00 | 0.87 | 0.67 - 1.15 |

These numbers were based on disease-related literature threshold and repeat sizes after genotyping correction. The inheritance was not considered. Alleles limited by the fragment length were deemed pathogenic. ‘Cases 2’ or ‘controls 2’ means the number of cases or controls that had both alleles expanded, ‘cases 1’ or ‘controls 1’ that one allele was expanded in these individuals. Premutation p-values were based on the number of premutation carriers (not premutation and pathogenic carriers). P is the uncorrected p-value and P_bon_ is the p-value Bonferroni corrected for the number of STRs and thresholds tested per STR. ‘95% CI’ is the 95% confidence interval of the odds ratio (OR). STRs without carriers were not included.

**Supplementary Figure 10.** Repeat sizes of both alleles genotyped with ExpansionHunter were validated by PCR for the *HTT* (A), *DMPK* (B), and *CSTB* (C) STRs in 34, 33, and 6 samples, respectively. Each dot represents one allele. ‘Outlier’ denotes non-matching repeat sizes between ExpansionHunter and PCR. The dashed orange line indicates the intermediate literature threshold, while the dashed red line represents the pathogenic threshold.


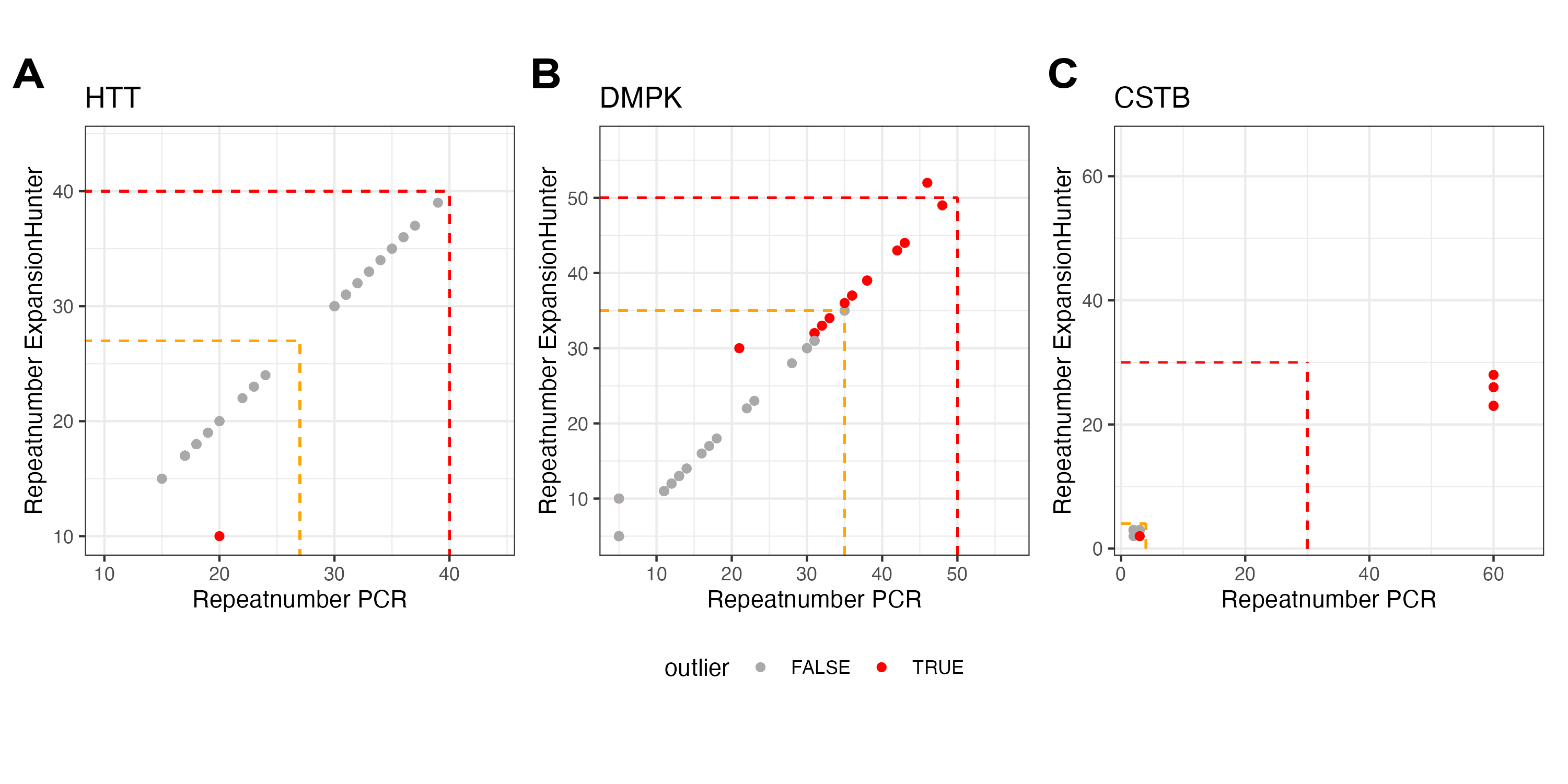


**Supplementary Table 13. Percentage pathogenic and premutated individuals without *C9orf72* expansions.**

| **RepeatID** | **Type** | **Threshold** | **Disease** | **Cases (%)** | **Controls (%)** |
| --- | --- | --- | --- | --- | --- |
| *AR* | pathogenic | 38 | SBMA | 0 (0) | 1 (0.058) |
|  | premutation | 35 | SBMA | 3 (0.062) | 1 (0.058) |
| *ATXN1* | pathogenic | 39 | SCA1 | 6 (0.12) | 1 (0.058) |
|  | premutation | 33 | SCA1 | 579 (12) | 170 (9.9) |
| *ATXN2* | pathogenic | 33 | SCA2 | 23 (0.48) | 2 (0.12) |
|  | premutation | 29 | SCA2 | 127 (2.6) | 13 (0.76) |
| *ATXN3* | pathogenic | 60 | SCA3 | 0 (0) | 1 (0.058) |
|  | premutation | 45 | SCA3 | 0 (0) | 1 (0.058) |
| *ATXN8* | pathogenic | 80 | SCA8 | 53 (1.1) | 18 (1) |
|  | premutation | 51 | SCA8 | 28 (0.57) | 7 (0.4) |
| *C9ORF72* | pathogenic | 30 | ALS/FTD | 0 (0) | 0 (0) |
|  | premutation | 24 | ALS/FTD | 6 (0.13) | 2 (0.12) |
| *CACNA1A* | pathogenic | 20 | SCA6 | 1 (0.021) | 0 (0) |
|  | premutation | 19 | SCA6 | 0 (0) | 0 (0) |
| *CBL* | pathogenic | 100 | FRA11B | 0 (0) | 0 (0) |
| *DMPK* | pathogenic | 50 | DM1 | 5 (0.1) | 2 (0.12) |
|  | premutation | 35 | DM1 | 28 (0.57) | 12 (0.69) |
| *GIPC1* | pathogenic | 73 | OPDM2 | 1 (0.021) | 0 (0) |
|  | premutation | 32 | OPDM2 | 7 (0.15) | 4 (0.23) |
| *HTT* | pathogenic | 40 | HD | 5 (0.1) | 1 (0.058) |
|  | premutation | 27 | HD | 301 (6.2) | 105 (6.1) |
| *NIPA1* | pathogenic | 10 | HSP6 | 171 (3.6) | 56 (3.3) |
| *NOP56* | pathogenic | 650 | SCA36 | 1 (0.02) | 0 (0) |
| *PABPN1* | pathogenic | 8 | OPMD | 2 (0.041) | 2 (0.12) |
| *RFC1* | pathogenic | 400 | CANVAS | 289 (6) | 96 (5.6) |
| *TCF4* | pathogenic | 80 | FECD3 | 211 (4.4) | 77 (4.5) |
|  | premutation | 41 | FECD3 | 192 (4) | 79 (4.6) |

These numbers were based on literature thresholds and disease-associated inheritance. Alleles limited by the fragment length limited were deemed pathogenic. STRs without pathogenic and premutation carriers were not included.

**Supplementary Table 14.** **Clinical re-evaluation of patients with a pathogenic STR expansion associated to another disease.**

| **Gene** | **Disease description** | **Pathogenic patients** | **Typical ALS** | **STR-associated disease** |
| --- | --- | --- | --- | --- |
| *AR* | Spinal and bulbar muscular atrophy, Kennedy disease | 2 | 1 | 1 |
| *ATXN1* | Spinocerebellar ataxia 1 | 6 | 2 |  |
| *ATXN2* | Spinocerebellar ataxia 2 | 23 | 11 |  |
| *ATXN8* | Spinocerebellar ataxia 8 | 55 | 28 |  |
| *CACNA1A* | Spinocerebellar ataxia 6 | 1 |  |  |
| *DMPK* | Myotonic dystrophy 1 | 5 | 1 |  |
| *FXN* | Friedreich's ataxia | 1 | 0 | 1 |
| *GIPC1* | Oculopharyngodistal myopathy type 2 | 3 | 2 | 1 |
| *HTT* | Huntington's disease | 5 | 1 |  |
| *NOP56* | Spinocerebellar ataxia 36 | 2 | 1* | 1 |
| *PABPN1* | Oculopharyngeal muscular dystrophy | 2 | 1 |  |

The genotyping of these pathogenic alleles was confirmed by inspecting the read-aligned plots with REViewer. The diagnosis of 52 out of 105 patients with a pathogenic STR expansion associated to another disease were re-evaluated by clinicians. Patients with a pathogenic expansion in *NIPA1*, *RFC1* and *TCF4* were not included in clinical re-evaluation. Four of the re-evaluated patients did not have ALS but the disease associated to the STR. *This patient had family members with ataxia.

**Supplementary Figure 11. Repeat size distribution differences *TCF4*.** *TCF4* repeat size distribution differences between Project MinE controls (n = 1,746) and gnomAD (n = 4,930) (A), and comparison of the mean ExpansionHunter repeat size and true repeat size between 360 bp and 450 bp DNA fragment lengths in *TCF4* (B). For each repeat size, reads were simulated and aligned to the repeat locus using simulate_str_expansions.py from <https://github.com/broadinstitute/str-analysis/tree/main/str_analysis>, followed by genotyping with ExpansionHunter. Each repeat size was simulated 50 times. The upper and lower boundaries with grey area depict the mean of the repeat size confidence interval. The dashed horizontal lines depict the 450 bp (orange) and 360 bp (blue) fragment length.


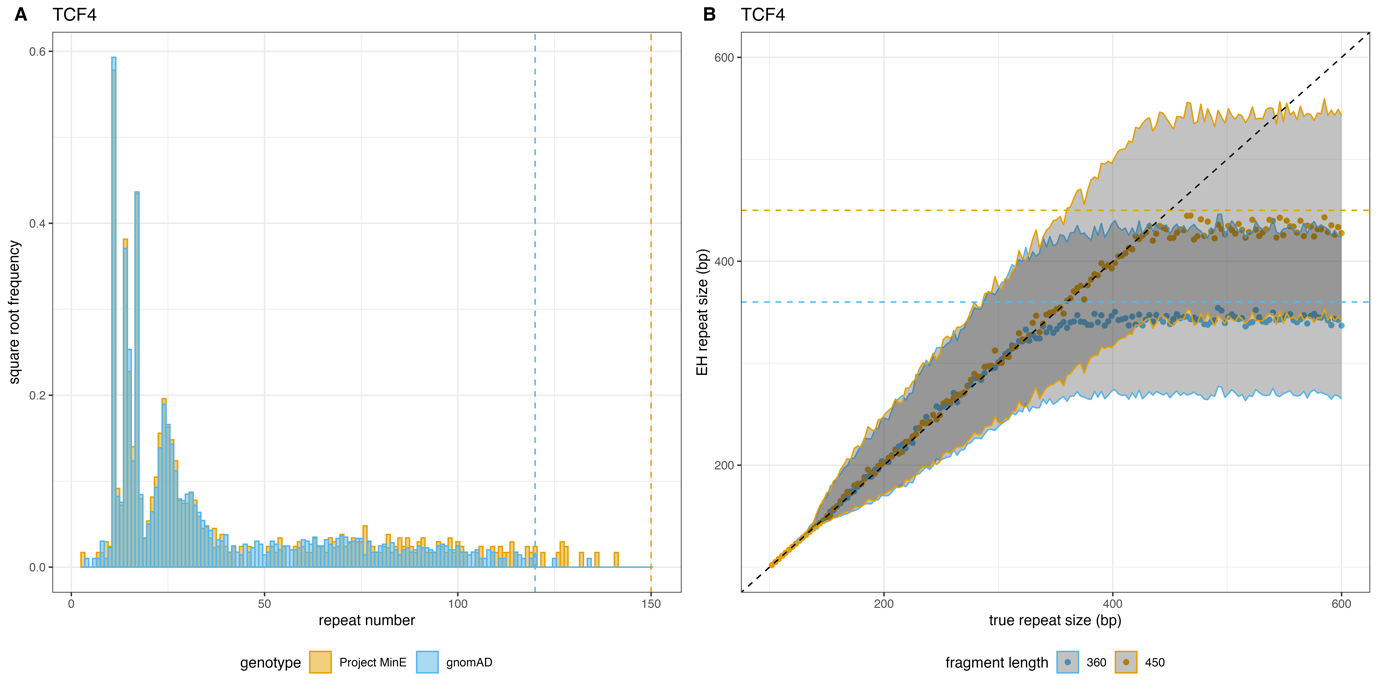


**Supplementary Figure 12. REViewer example *ATXN1*.** Read alignment created with REViewer of *ATXN1* of a gnomAD sample. According to ExpansionHunter this individual has one allele with 30 repeat units and one with 44 repeat units. Evidence for the longest allele is supported by a single flanking read exhibiting misalignment to the reference genome. This sample actually possesses two alleles, each containing 30 repeat units, as inferred from the alignment of motif interruptions.

**
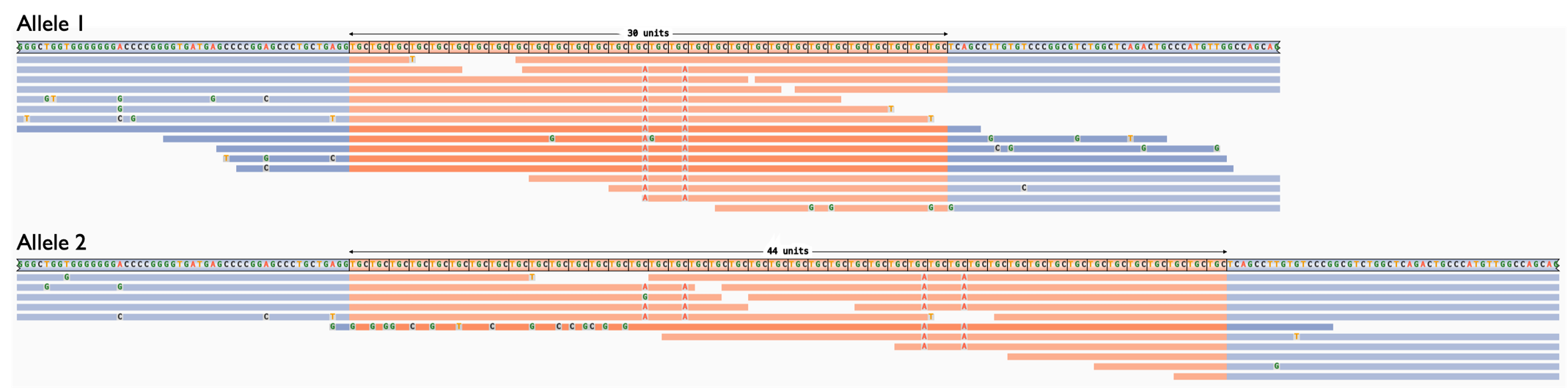
**

**Supplementary Table 15. ALS susceptibility association with best threshold analysis.**

| **RepeatID** | **Threshold** | **# Thresholds** | **Cases 1 (%)** | **Cases 2 (%)** | **Controls 1 (%)** | **Controls 2 (%)** | **Pfdr** | **Pfdrbon** | **OR** | **95% CI** |
| --- | --- | --- | --- | --- | --- | --- | --- | --- | --- | --- |
| *C9ORF72* | 32 | 301 | 329 (6.4) | 0 (0) | 7 (0.41) | 0 (0) | < 2.2E-16 | < 2.2E-16 | 18 | 9.24 - 41 |
| *ATXN2* | 30 | 27 | 121 (2.3) | 5 (0.097) | 9 (0.52) | 0 (0) | 5.7E-06 | 1.6E-04 | 4.17 | 2.27 - 8.67 |
| *ATXN1* | 34 | 27 | 400 (7.7) | 7 (0.13) | 107 (6.2) | 1 (0.058) | 0.051 | 1.00 | 1.40 | 1.13 - 1.75 |
| *CSTB* | 20 | 23 | 20 (0.39) | 0 (0) | 0 (0) | 0 (0) | 0.12 | 1.00 | 13 | 1.71 - 1611 |
| *HTT* | 14 | 36 | 240 (4.6) | 4967 (95) | 52 (3.0) | 1685 (97) | 0.15 | 1.00 | 0.66 | 0.48 - 0.89 |
| *NOP56* | 4 | 13 | 18 (0.34) | 5203 (100) | 9 (0.52) | 1728 (99) | 0.22 | 1.00 | 2.65 | 1.20 - 5.84 |
| *ARX_PRTS* | 8 | 6 | 3113 (61) | 2004 (39) | 881 (52) | 824 (48) | 0.24 | 1.00 | 0.23 | 0.025 - 0.95 |
| *RFC1* | 61 | 114 | 1249 (25) | 760 (15) | 387 (23) | 233 (14) | 0.24 | 1.00 | 1.11 | 1.03 - 1.21 |
| *ATXN3* | 20 | 30 | 2564 (49) | 1738 (33) | 813 (47) | 634 (36) | 0.29 | 1.00 | 0.91 | 0.84 - 0.98 |
| *DMPK* | 15 | 47 | 1209 (23) | 95 (1.8) | 449 (26) | 37 (2.1) | 0.31 | 1.00 | 0.85 | 0.76 - 0.96 |
| *AR* | 35 | 35 | 9 (0.17) | 0 (0) | 10 (0.58) | 0 (0) | 0.31 | 1.00 | 0.35 | 0.14 - 0.87 |
| *DAB1* | 79 | 100 | 130 (2.5) | 9 (0.18) | 30 (1.8) | 1 (0.059) | 0.32 | 1.00 | 1.57 | 1.09 - 2.33 |
| *CACNA1A* | 12 | 16 | 2543 (49) | 1514 (29) | 875 (50) | 549 (32) | 0.37 | 1.00 | 0.91 | 0.84 - 0.99 |
| *JPH3* | 14 | 17 | 368 (7.0) | 4855 (93) | 107 (6.1) | 1637 (94) | 0.40 | 1.00 | 0.82 | 0.66 - 1.03 |
| *GIPC1* | 8 | 37 | 46 (0.89) | 5121 (99) | 7 (0.41) | 1721 (100) | 0.58 | 1.00 | 0.41 | 0.17 - 0.86 |
| *ATXN7* | 21 | 19 | 0 (0) | 0 (0) | 2 (0.12) | 0 (0) | 0.61 | 1.00 | 0.12 | 0.00088 - 1.51 |
| *PABPN1* | 8 | 6 | 2 (0.038) | 0 (0) | 2 (0.11) | 0 (0) | 0.72 | 1.00 | 0.25 | 0.036 - 1.73 |
| *ATN1* | 19 | 27 | 2312 (44) | 2333 (45) | 723 (41) | 839 (48) | 0.76 | 1.00 | 0.93 | 0.86 - 1.02 |
| *PPP2R2B* | 11 | 23 | 2376 (45) | 701 (13) | 823 (47) | 232 (13) | 0.80 | 1.00 | 0.94 | 0.87 - 1.02 |
| *GLS* | 21 | 35 | 189 (3.6) | 0 (0) | 83 (4.8) | 0 (0) | 0.82 | 1.00 | 0.75 | 0.57 - 0.98 |
| *ATXN10* | 19 | 18 | 189 (3.6) | 1 (0.019) | 71 (4.1) | 1 (0.058) | 0.93 | 1.00 | 0.83 | 0.63 - 1.11 |
| *TCF4* | 17 | 131 | 2502 (48) | 864 (17) | 833 (48) | 329 (19) | 0.94 | 1.00 | 0.91 | 0.84 - 0.99 |
| *CBL* | 6 | 35 | 13 (0.25) | 5205 (100) | 0 (0) | 1740 (100) | 0.99 | 1.00 | 0.13 | 0.00097 - 0.99 |
| *ATXN8* | 18 | 106 | 859 (16) | 47 (0.90) | 320 (18) | 18 (1.0) | 1.00 | 1.00 | 0.89 | 0.79 - 1.02 |
| *ARX_EIEE* | 17 | 11 | 1 (0.019) | 0 (0) | 1 (0.058) | 0 (0) | 1.00 | 1.00 | 0.31 | 0.025 - 3.81 |
| *FXN* | 2 | 107 | 76 (1.5) | 4952 (98) | 17 (1.0) | 1665 (99) | 1.00 | 1.00 | 0.68 | 0.39 - 1.13 |
| *LRP12* | 30 | 24 | 1 (0.020) | 0 (0) | 0 (0) | 0 (0) | 1.00 | 1.00 | 0.013 | 0.00006 - 2.81 |
| *NIPA1* | 10 | 9 | 176 (3.4) | 3 (0.059) | 57 (3.4) | 0 (0) | 1.00 | 1.00 | 1.12 | 0.83 - 1.54 |

‘Cases 2’ or ‘controls 2’ means the number of cases or controls that had both alleles expanded, ‘cases 1’ or ‘controls 1’ that one allele was expanded in these individuals. P_fdr_ is the p-value corrected for the number of thresholds tested per STR with FDR correction. P_fdrbon_ is the P_fdr_ value Bonferroni corrected for the number of STRs tested. ‘95% CI’ is the 95% confidence interval of the odds ratio (OR).

**Supplementary Figure 13. Best threshold analysis of *C9orf72* to ALS susceptibility.** A) Negative base-10 logarithm of the p-value (-10logP, blue) and odds ratio (OR, orange) upon Firth’s Bias-Reduced Logistic Regression analysis on each existing repeat size up to 90 repeat units in 5,237 cases and 1,746 controls of Project MinE. Vertical line is the best threshold at 32 repeat units. Horizontal line is the significant p-value after correction (P_fdrbon_). B) OR and 95% confidence interval (grey) for each existing repeat size up to 90 repeat units. Vertical line is the best threshold at 32 repeat units. Horizontal line is OR = 1. The best threshold was identified as the repeat size of which the OR had the highest distance from the line drawn between the first and last OR of the significant repeat sizes (diagonal red line).


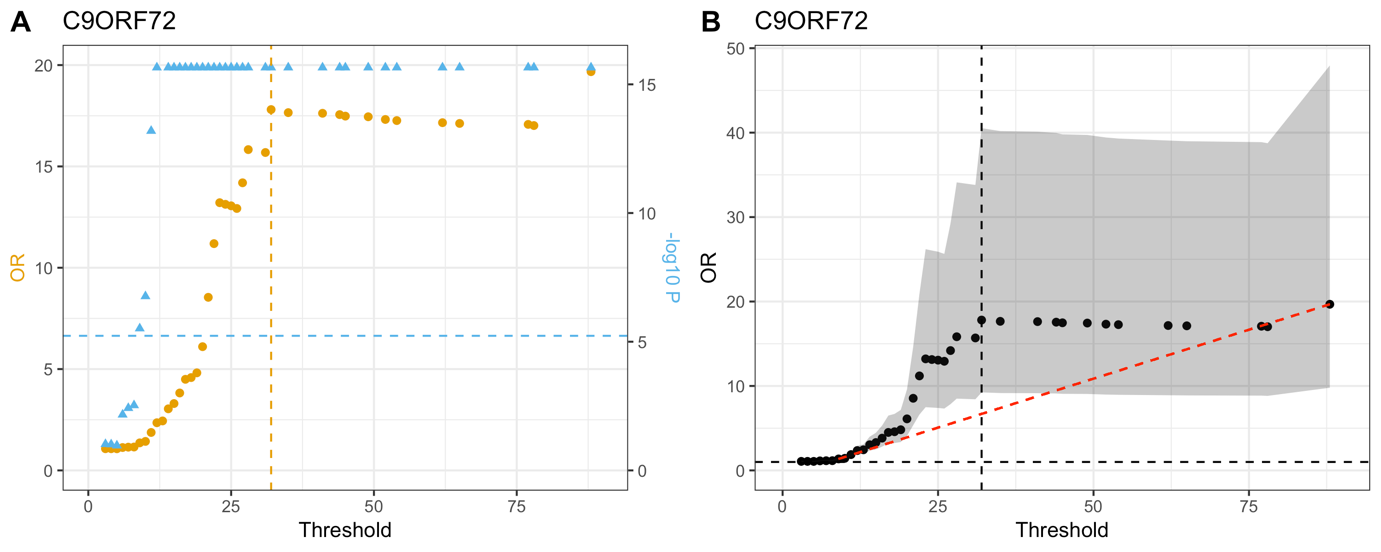


**Supplementary Figure 14. Best threshold analysis of *ATXN2* to ALS susceptibility.** A) Negative base-10 logarithm of the p-value (-10logP, blue) and odds ratio (OR, orange) upon Firth’s Bias-Reduced Logistic Regression analysis on each existing repeat size in 5,237 cases and 1,746 controls of Project MinE. Horizontal line is the significant p-value after correction (P_fdrbon_). B) OR and 95% confidence interval (grey) for each existing repeat size. Horizontal line is OR = 1.


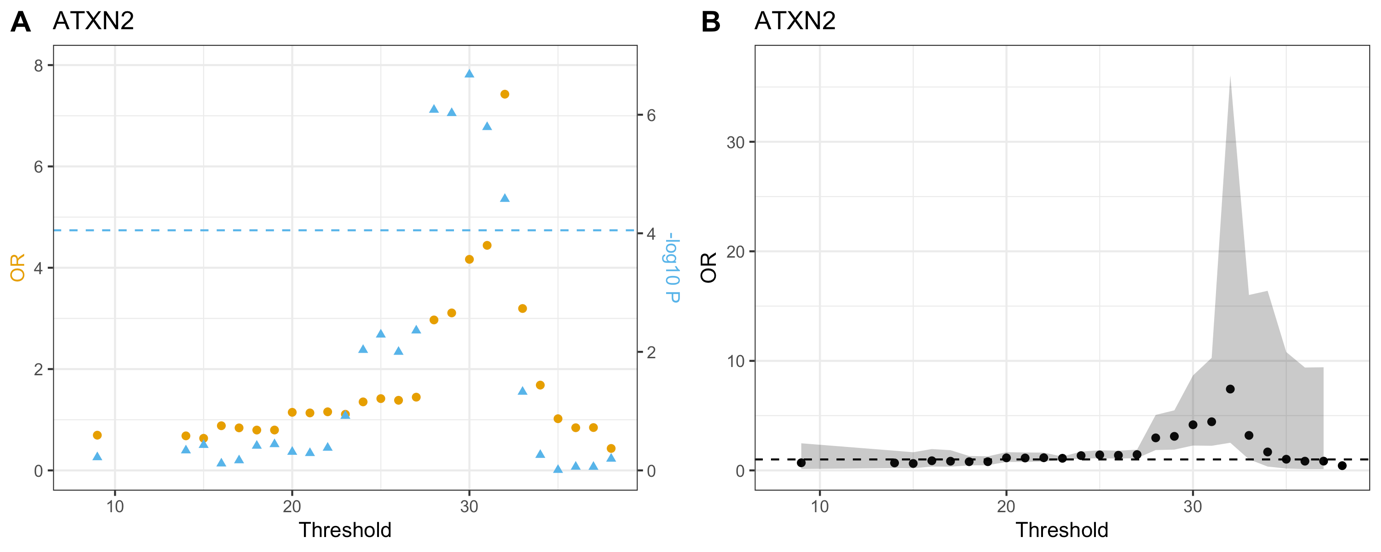


**Supplementary Figure 15. Best threshold analysis of *ATXN1* to ALS susceptibility.** A) Negative base-10 logarithm of the p-value (-10logP, blue) and odds ratio (OR, orange) upon Firth’s Bias-Reduced Logistic Regression analysis on each existing repeat size in 5,237 cases and 1,746 controls of Project MinE. B) OR and 95% confidence interval (grey) for each existing repeat size. Horizontal line is OR = 1.


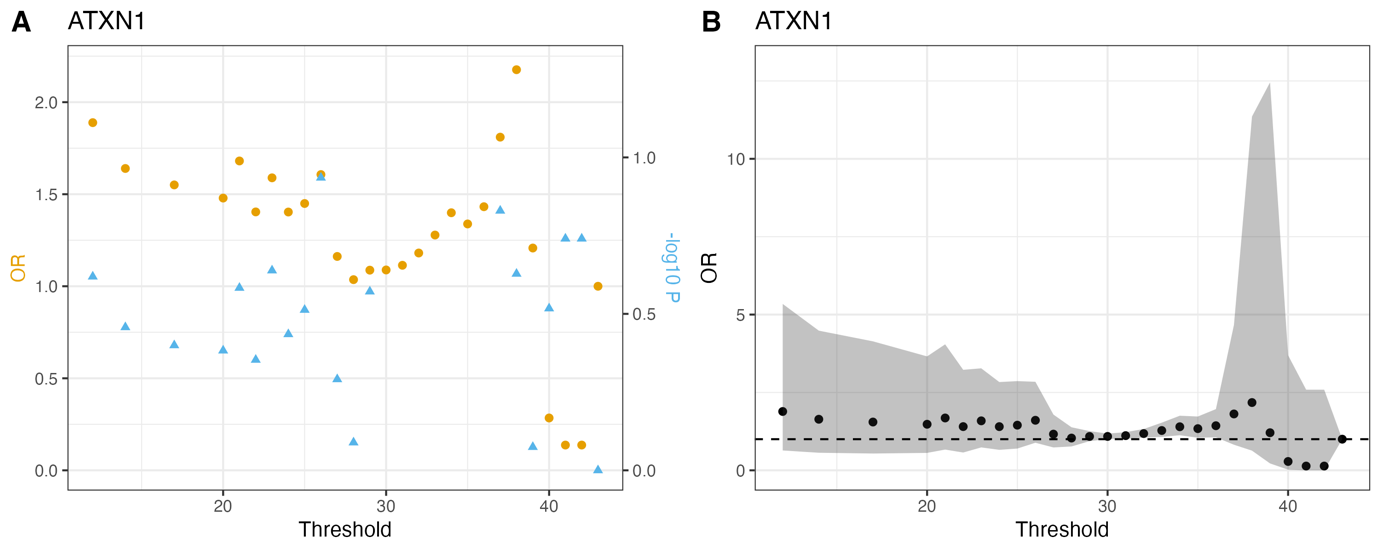


**Supplementary Figure 16.** Age distribution of Project MinE (n = 20) and gnomAD (n = 18) individuals with an expanded *CSTB* allele (20 or more repeat units).

**
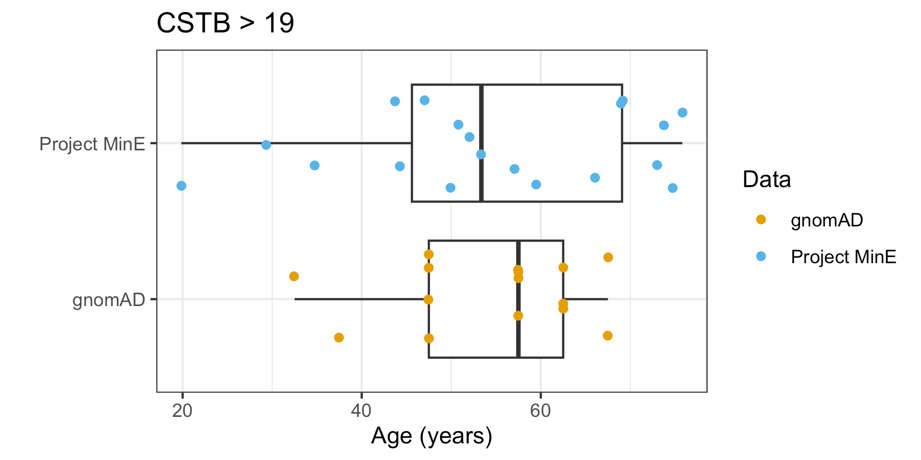
**

**Supplementary Table 16. Motif changes in disease-associated STRs.**

| **RepeatID** | **Motif** | **Motif EHdn** | **Cases (%)** | **Controls (%)** | **OR** | **95% CI** | **P** | **Pbon** |
| --- | --- | --- | --- | --- | --- | --- | --- | --- |
| *ATXN8* | CTA/CTG | AGCGGC | 1 (0.018) | 0 (0) | 1.09 | 0.72 - 2.21 | 0.68 | 1.00 |
| *BEAN1* | TAAAA | AAAATAAAATAT | 0 (0) | 1 (0.055) | 0.83 | 0.58 - 1.03 | 0.092 | 1.00 |
| *BEAN1* | TAAAA | AATAG | 5 (0.090) | 3 (0.17) | 0.93 | 0.83 - 1.05 | 0.20 | 1.00 |
| *BEAN1* | TAAAA | AACAT | 33 (0.60) | 13 (0.72) | 0.93 | 0.83 - 1.06 | 0.27 | 1.00 |
| *BEAN1* | TAAAA | AAAATAACAT | 10 (0.18) | 4 (0.22) | 0.92 | 0.76 - 1.15 | 0.41 | 1.00 |
| *BEAN1* | TAAAA | AAACT | 11 (0.20) | 6 (0.33) | 0.96 | 0.83 - 1.12 | 0.58 | 1.00 |
| *BEAN1* | TAAAA | AAAATAAACT | 24 (0.43) | 9 (0.50) | 0.98 | 0.91 - 1.07 | 0.62 | 1.00 |
| *BEAN1* | TAAAA | AAAATAAACTAAACT | 1 (0.018) | 0 (0) | 0.95 | 0.78 - 1.32 | 0.66 | 1.00 |
| *BEAN1* | TAAAA | AAAAC | 2 (0.036) | 0 (0) | 1.06 | 0.82 - 1.67 | 0.67 | 1.00 |
| *BEAN1* | TAAAA | AATAC | 82 (1.5) | 23 (1.3) | 1.01 | 0.97 - 1.04 | 0.72 | 1.00 |
| *BEAN1* | TAAAA | AAATG | 4 (0.072) | 1 (0.055) | 1.02 | 0.87 - 1.40 | 0.80 | 1.00 |
| *BEAN1* | TAAAA | AAAATAGAAT | 1 (0.018) | 0 (0) | 0.98 | 0.77 - 1.47 | 0.87 | 1.00 |
| *BEAN1* | TAAAA | AAAGT | 1 (0.018) | 0 (0) | 0.99 | 0.66 - 1.98 | 0.98 | 1.00 |
| *C9ORF72* | GGCCCC | CCCCCGCCCCGGCCCCGG | 9 (0.16) | 0 (0) | 3.01 | 0.76 - 652 | 0.15 | 1.00 |
| *DAB1* | AAAAT/AAAT | AATAC | 1 (0.018) | 0 (0) | 0.86 | 0.53 - 1.93 | 0.60 | 1.00 |
| *DAB1* | AAAAT/AAAT | AAAAAAAAATAAAAT | 4 (0.072) | 2 (0.11) | 0.43 | 0.067 - 3.08 | 0.36 | 1.00 |
| *FXN* | A/GAA | AAGAGG | 0 (0) | 1 (0.055) | 0.72 | 0.31 - 1.18 | 0.19 | 1.00 |
| *FXN* | A/GAA | AAGGAG | 11 (0.20) | 2 (0.11) | 1.03 | 0.94 - 1.18 | 0.55 | 1.00 |
| *FXN* | A/GAA | AAGAAGAAGAGG | 1 (0.018) | 0 (0) | 0.88 | 0.43 - 2.97 | 0.75 | 1.00 |
| *FXN* | A/GAA | AAGAAGAAGAAGAAGAGG | 2 (0.036) | 0 (0) | 1.14 | 0.39 - 11 | 0.83 | 1.00 |
| *RFC1* | AARRG | AAGGT | 1 (0.018) | 1 (0.055) | 0.71 | 0.25 - 1.04 | 0.077 | 1.00 |
| *RFC1* | AARRG | AAGAG | 204 (3.7) | 71 (3.9) | 0.97 | 0.94 - 1.01 | 0.20 | 1.00 |
| *RFC1* | AARRG | AAGGG* | 326 (5.9) | 83 (4.6) | 1.03 | 0.99 - 1.07 | 0.20 | 1.00 |
| *RFC1* | AARRG | AGGGC* | 2 (0.036) | 2 (0.11) | 0.86 | 0.64 - 1.11 | 0.23 | 1.00 |
| *RFC1* | AARRG | AAAAGAAAGG | 7 (0.13) | 5 (0.28) | 0.94 | 0.86 - 1.04 | 0.24 | 1.00 |
| *RFC1* | AARRG | AAAAGG | 6 (0.11) | 2 (0.11) | 0.94 | 0.75 - 1.21 | 0.60 | 1.00 |
| *RFC1* | AARRG | AAAAAGGAAAGG | 1 (0.018) | 1 (0.055) | 0.97 | 0.86 - 1.15 | 0.63 | 1.00 |
| *RFC1* | AARRG | ACAGG* | 1 (0.018) | 0 (0) | 0.91 | 0.65 - 1.62 | 0.65 | 1.00 |
| *RFC1* | AARRG | AAGGC | 2 (0.036) | 0 (0) | 1.07 | 0.82 - 1.82 | 0.65 | 1.00 |
| *RFC1* | AARRG | AACGG | 2 (0.036) | 0 (0) | 1.10 | 0.73 - 2.60 | 0.70 | 1.00 |
| *RFC1* | AARRG | AAGAC | 36 (0.65) | 13 (0.72) | 1.00 | 0.97 - 1.05 | 0.82 | 1.00 |
| *RFC1* | AARRG | AACAG | 1 (0.018) | 1 (0.055) | 0.98 | 0.79 - 1.27 | 0.88 | 1.00 |
| *RFC1* | AARRG | AAAGGG | 328 (5.9) | 116 (6.4) | 1.00 | 0.96 - 1.05 | 0.88 | 1.00 |
| *RFC1* | AARRG | AAAAGGAAAGG | 2 (0.036) | 0 (0) | 0.96 | 0.60 - 2.12 | 0.88 | 1.00 |
| *SAMD12* | TAAAA | AAAATAACATAACATAACAT | 1 (0.018) | 0 (0) | 0.90 | 0.22 - 10 | 0.90 | 1.00 |
| *SAMD12* | TAAAA | AACAT | 1 (0.018) | 0 (0) | 0.99 | 0.70 - 1.75 | 0.95 | 1.00 |

Motif changes and the number of individuals per motif change were identified with ExpansionHunter Denovo (EHdn) and validated with visual inspection of the read-aligned plots. STRs with motif changes based on less than five in-repeat reads were not considered, nor motif changes with a single bp. No ALS-associated motif changes were observed, not with individual motifs (this table) nor with all motifs added up per STR. ‘95% CI’ is the 95% confidence interval of the odds ratio (OR). P is the uncorrected p-value. P_bon_ is the p-value Bonferroni corrected for the number of STRs and motifs per STRs tested. ‘*’ denotes disease-associated motifs based on literature.

**Supplementary Table 17. STR motifs in potential *RFC1* expansions.**

| **RepeatID** | **Motif** | **Cases (%)** | **Controls (%)** | **OR** | **95% CI** | **P** | **Pbon** |
| --- | --- | --- | --- | --- | --- | --- | --- |
| *RFC1* | AAAAG | 187 (3.6) | 59 (3.4) | 1.03 | 0.76 - 1.41 | 0.87 | 1.00 |
| *RFC1* | AAAAG AAGGG | 48 (0.92) | 13 (0.74) | 1.25 | 0.42 - 3.57 | 0.68 | 1.00 |
| *RFC1* | AAAAG AAAGG | 39 (0.74) | 13 (0.74) | 1.20 | 0.55 - 2.73 | 0.65 | 1.00 |
| *RFC1* | AAGGG | 8 (0.15) | 1 (0.057) | 1.51 | 0.29 - 16 | 0.66 | 1.00 |
| *RFC1* | AAAAG AAAGGG | 6 (0.11) | 3 (0.17) | 1.18 | 0.28 - 5.76 | 0.83 | 1.00 |
| *RFC1* | AAAGG AAGGG | 4 (0.076) | 3 (0.17) | 0.11 | 0.00007 - 88 | 0.50 | 1.00 |
| *RFC1* | AAAGG | 4 (0.076) | 0 (0) | 3.34 | 0.29 - 462 | 0.38 | 1.00 |
| *RFC1* | AAAGGG AAGGG | 4 (0.076) | 0 (0) | 3.66 | 0.15 - 4471813646440029 | 0.45 | 1.00 |
| *RFC1* | AAAAG AAGAG | 2 (0.038) | 1 (0.057) | 0.44 | 0.025 - 8.83 | 0.56 | 1.00 |
| *RFC1* | AAAGG AAAGGG | 2 (0.038) | 1 (0.057) | 0.46 | 0.00018 - 97 | 0.72 | 1.00 |

STR motifs in *RFC1* and the number of individuals with a potential expansion were identified with ExpansionHunter Denovo and validated with visual inspection of the read-aligned plots. STRs with motifs based on less than five in-repeat reads were not considered, nor motifs with a single bp. Individuals were considered pathogenic expanded when both *RFC1* alleles were restricted by the fragment length. No association of the *RFC1* motifs with ALS was detected. ‘95% CI’ is the 95% confidence interval of the odds ratio (OR). P is the uncorrected p-value. P_bon_ is the p-value Bonferroni corrected for the number of motifs tested.

**Supplementary Table 18. ALS susceptibility association with repeat size distribution analysis.**

| **RepeatID** | **Analysis** | **OR** | **95% CI** | **P** | **Pbon** |
| --- | --- | --- | --- | --- | --- |
| *AR* | Max | 1.00 | 0.98 - 1.02 | 0.68 | 1.00 |
| *ARX_EIEE* | Max | 0.96 | 0.58 - 1.59 | 0.88 | 1.00 |
| *ARX_PRTS* | Max | 1.19 | 0.86 - 1.65 | 0.30 | 1.00 |
| *ATN1* | Max | 0.99 | 0.97 - 1.02 | 0.59 | 1.00 |
| *ATXN10* | Max | 0.99 | 0.96 - 1.02 | 0.56 | 1.00 |
| *ATXN1* | Max | 1.06 | 1.03 - 1.10 | 2.8E-04 | 0.016 |
| *ATXN2* | Max | 1.07 | 1.03 - 1.12 | 4.2E-04 | 0.023 |
| *ATXN3* | Max | 0.99 | 0.98 - 1.01 | 0.24 | 1.00 |
| *ATXN7* | Max | 1.03 | 0.98 - 1.09 | 0.18 | 1.00 |
| *ATXN8* | Max | 1.00 | 0.99 - 1.00 | 0.88 | 1.00 |
| *C9ORF72* | Max | 1.01 | 1.00 - 1.01 | 1.8E-11 | 1.0E-09 |
| *CACNA1A* | Max | 0.96 | 0.91 - 1.01 | 0.094 | 1.00 |
| *CBL* | Max | 1.00 | 0.98 - 1.02 | 0.99 | 1.00 |
| *CSTB* | Max | 1.04 | 0.99 - 1.10 | 0.12 | 1.00 |
| *DAB1* | Max | 1.00 | 1.00 - 1.01 | 0.11 | 1.00 |
| *DMPK* | Max | 0.99 | 0.99 - 1.00 | 0.25 | 1.00 |
| *FXN* | Max | 1.00 | 1.00 - 1.00 | 0.97 | 1.00 |
| *GIPC1* | Max | 1.00 | 0.98 - 1.02 | 0.98 | 1.00 |
| *GLS* | Max | 1.00 | 0.98 - 1.01 | 0.69 | 1.00 |
| *HTT* | Max | 1.00 | 0.99 - 1.02 | 0.55 | 1.00 |
| *JPH3* | Max | 0.97 | 0.93 - 1.02 | 0.26 | 1.00 |
| *LRP12* | Max | 0.99 | 0.97 - 1.02 | 0.72 | 1.00 |
| *NIPA1* | Max | 1.05 | 0.92 - 1.19 | 0.47 | 1.00 |
| *NOP56* | Max | 1.01 | 0.98 - 1.05 | 0.49 | 1.00 |
| *PABPN1* | Max | 0.71 | 0.42 - 1.20 | 0.19 | 1.00 |
| *PPP2R2B* | Max | 0.99 | 0.97 - 1.01 | 0.32 | 1.00 |
| *RFC1* | Max | 1.00 | 1.00 - 1.00 | 0.042 | 1.00 |
| *TCF4* | Max | 1.00 | 1.00 - 1.00 | 0.66 | 1.00 |
| *AR* | Sum | 1.01 | 1.00 - 1.02 | 0.24 | 1.00 |
| *ARX_EIEE* | Sum | 1.01 | 0.91 - 1.12 | 0.83 | 1.00 |
| *ARX_PRTS* | Sum | 0.92 | 0.82 - 1.04 | 0.17 | 1.00 |
| *ATN1* | Sum | 0.99 | 0.98 - 1.00 | 0.20 | 1.00 |
| *ATXN10* | Sum | 1.00 | 0.98 - 1.02 | 0.86 | 1.00 |
| *ATXN1* | Sum | 1.02 | 1.01 - 1.04 | 0.010 | 0.57 |
| *ATXN2* | Sum | 1.03 | 1.01 - 1.05 | 2.6E-03 | 0.15 |
| *ATXN3* | Sum | 0.99 | 0.98 - 1.00 | 0.067 | 1.00 |
| *ATXN7* | Sum | 1.02 | 0.99 - 1.04 | 0.27 | 1.00 |
| *ATXN8* | Sum | 1.00 | 0.99 - 1.00 | 0.75 | 1.00 |
| *C9ORF72* | Sum | 1.01 | 1.00 - 1.01 | 1.5E-11 | 8.5E-10 |
| *CACNA1A* | Sum | 0.98 | 0.96 - 1.00 | 0.099 | 1.00 |
| *CBL* | Sum | 1.00 | 0.98 - 1.02 | 0.80 | 1.00 |
| *CSTB* | Sum | 1.03 | 0.99 - 1.07 | 0.21 | 1.00 |
| *DAB1* | Sum | 1.00 | 1.00 - 1.00 | 0.18 | 1.00 |
| *DMPK* | Sum | 1.00 | 0.99 - 1.00 | 0.41 | 1.00 |
| *FXN* | Sum | 1.00 | 1.00 - 1.00 | 0.96 | 1.00 |
| *GIPC1* | Sum | 0.99 | 0.98 - 1.01 | 0.56 | 1.00 |
| *GLS* | Sum | 1.00 | 0.99 - 1.01 | 0.87 | 1.00 |
| *HTT* | Sum | 1.00 | 0.99 - 1.01 | 0.80 | 1.00 |
| *JPH3* | Sum | 0.98 | 0.95 - 1.01 | 0.14 | 1.00 |
| *LRP12* | Sum | 1.00 | 0.99 - 1.01 | 0.97 | 1.00 |
| *NIPA1* | Sum | 1.01 | 0.96 - 1.06 | 0.65 | 1.00 |
| *NOP56* | Sum | 1.01 | 0.99 - 1.04 | 0.22 | 1.00 |
| *PABPN1* | Sum | 0.92 | 0.63 - 1.34 | 0.65 | 1.00 |
| *PPP2R2B* | Sum | 0.99 | 0.97 - 1.01 | 0.32 | 1.00 |
| *RFC1* | Sum | 1.00 | 1.00 - 1.00 | 0.094 | 1.00 |
| *TCF4* | Sum | 1.00 | 1.00 - 1.00 | 0.41 | 1.00 |

The p-value was corrected for the number of analysis types tested and the number of STRs tested (P_bon_). ‘95% CI’ is the 95% confidence interval of the odds ratio (OR).

**Supplementary Table 19. ALS susceptibility association with log transformed repeat size distribution analysis.**

| **RepeatID** | **Analysis** | **OR** | **95% CI** | **P** | **Pbon** |
| --- | --- | --- | --- | --- | --- |
| *AR* | Log Max | 1.16 | 0.73 - 1.84 | 0.54 | 1.00 |
| *ARX_EIEE* | Log Max | 0.48 | 0.00029 - 768 | 0.84 | 1.00 |
| *ARX_PRTS* | Log Max | 3.36 | 0.27 - 41 | 0.34 | 1.00 |
| *ATN1* | Log Max | 0.88 | 0.56 - 1.40 | 0.60 | 1.00 |
| *ATXN1* | Log Max | 7.48 | 2.54 - 22 | 2.6E-04 | 0.015 |
| *ATXN10* | Log Max | 0.87 | 0.51 - 1.47 | 0.59 | 1.00 |
| *ATXN2* | Log Max | 5.73 | 2.10 - 16 | 6.7E-04 | 0.037 |
| *ATXN3* | Log Max | 0.87 | 0.66 - 1.15 | 0.34 | 1.00 |
| *ATXN7* | Log Max | 1.55 | 0.88 - 2.73 | 0.13 | 1.00 |
| *ATXN8* | Log Max | 0.93 | 0.77 - 1.13 | 0.47 | 1.00 |
| *C9ORF72* | Log Max | 1.28 | 1.21 - 1.36 | 1.9E-17 | 1.1E-15 |
| *CACNA1A* | Log Max | 0.61 | 0.34 - 1.10 | 0.099 | 1.00 |
| *CBL* | Log Max | 1.00 | 0.72 - 1.39 | 0.99 | 1.00 |
| *CSTB* | Log Max | 1.15 | 0.84 - 1.59 | 0.38 | 1.00 |
| *DAB1* | Log Max | 1.07 | 0.97 - 1.18 | 0.18 | 1.00 |
| *DMPK* | Log Max | 0.94 | 0.83 - 1.06 | 0.30 | 1.00 |
| *FXN* | Log Max | 1.04 | 0.92 - 1.17 | 0.52 | 1.00 |
| *GIPC1* | Log Max | 1.02 | 0.65 - 1.61 | 0.94 | 1.00 |
| *GLS* | Log Max | 0.92 | 0.76 - 1.13 | 0.43 | 1.00 |
| *HTT* | Log Max | 1.11 | 0.78 - 1.57 | 0.56 | 1.00 |
| *JPH3* | Log Max | 0.64 | 0.31 - 1.34 | 0.24 | 1.00 |
| *LRP12* | Log Max | 0.91 | 0.65 - 1.29 | 0.60 | 1.00 |
| *NIPA1* | Log Max | 1.48 | 0.51 - 4.32 | 0.47 | 1.00 |
| *NOP56* | Log Max | 1.05 | 0.85 - 1.31 | 0.63 | 1.00 |
| *PABPN1* | Log Max | 0.094 | 0.0028 - 3.20 | 0.19 | 1.00 |
| *PPP2R2B* | Log Max | 0.84 | 0.63 - 1.12 | 0.24 | 1.00 |
| *RFC1* | Log Max | 1.07 | 1.00 - 1.15 | 0.054 | 1.00 |
| *TCF4* | Log Max | 0.98 | 0.88 - 1.09 | 0.67 | 1.00 |
| *AR* | Log Sum | 1.34 | 0.85 - 2.10 | 0.21 | 1.00 |
| *ARX_EIEE* | Log Sum | 1.29 | 0.16 - 10 | 0.81 | 1.00 |
| *ARX_PRTS* | Log Sum | 0.23 | 0.024 - 2.24 | 0.21 | 1.00 |
| *ATN1* | Log Sum | 0.77 | 0.51 - 1.17 | 0.22 | 1.00 |
| *ATXN1* | Log Sum | 2.50 | 1.11 - 5.65 | 0.027 | 1.00 |
| *ATXN10* | Log Sum | 1.00 | 0.54 - 1.87 | 0.99 | 1.00 |
| *ATXN2* | Log Sum | 2.54 | 1.23 - 5.21 | 0.011 | 0.63 |
| *ATXN3* | Log Sum | 0.79 | 0.59 - 1.04 | 0.093 | 1.00 |
| *ATXN7* | Log Sum | 1.27 | 0.81 - 1.99 | 0.30 | 1.00 |
| *ATXN8* | Log Sum | 0.93 | 0.74 - 1.16 | 0.51 | 1.00 |
| *C9ORF72* | Log Sum | 1.37 | 1.28 - 1.47 | 3.3E-19 | 1.9E-17 |
| *CACNA1A* | Log Sum | 0.70 | 0.46 - 1.05 | 0.086 | 1.00 |
| *CBL* | Log Sum | 0.91 | 0.56 - 1.49 | 0.72 | 1.00 |
| *CSTB* | Log Sum | 1.07 | 0.78 - 1.47 | 0.67 | 1.00 |
| *DAB1* | Log Sum | 1.07 | 0.95 - 1.20 | 0.30 | 1.00 |
| *DMPK* | Log Sum | 0.94 | 0.81 - 1.09 | 0.40 | 1.00 |
| *FXN* | Log Sum | 1.01 | 0.87 - 1.18 | 0.89 | 1.00 |
| *GIPC1* | Log Sum | 0.81 | 0.50 - 1.30 | 0.38 | 1.00 |
| *GLS* | Log Sum | 0.95 | 0.76 - 1.20 | 0.69 | 1.00 |
| *HTT* | Log Sum | 0.94 | 0.62 - 1.41 | 0.76 | 1.00 |
| *JPH3* | Log Sum | 0.52 | 0.22 - 1.23 | 0.14 | 1.00 |
| *LRP12* | Log Sum | 1.01 | 0.78 - 1.31 | 0.93 | 1.00 |
| *NIPA1* | Log Sum | 1.10 | 0.60 - 2.04 | 0.75 | 1.00 |
| *NOP56* | Log Sum | 1.17 | 0.91 - 1.50 | 0.22 | 1.00 |
| *PABPN1* | Log Sum | 0.67 | 0.018 - 25 | 0.83 | 1.00 |
| *PPP2R2B* | Log Sum | 0.79 | 0.52 - 1.19 | 0.26 | 1.00 |
| *RFC1* | Log Sum | 1.07 | 0.99 - 1.15 | 0.095 | 1.00 |
| *TCF4* | Log Sum | 0.93 | 0.81 - 1.06 | 0.28 | 1.00 |

The p-value was corrected for the number of analysis types tested and the number of STRs tested (P_bon_). ‘95% CI’ is the 95% confidence interval of the odds ratio (OR).


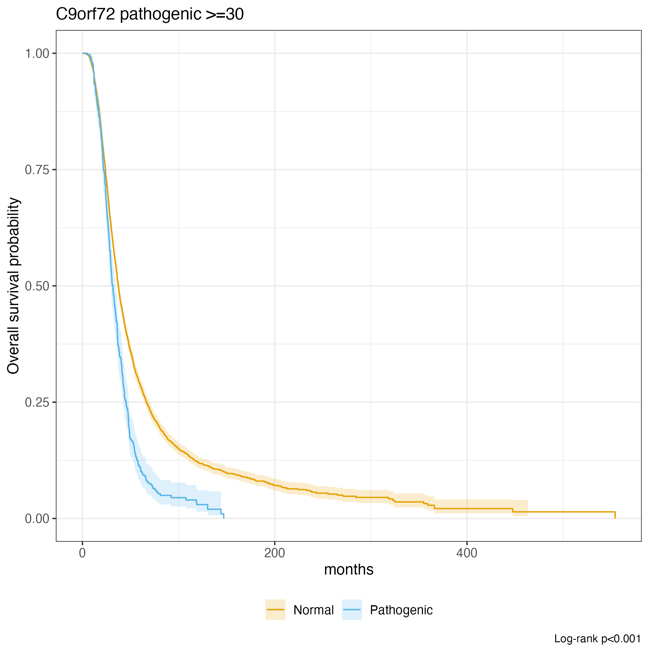
**Supplementary Figure 17. ALS survival analysis of pathogenic *C9orf72* with Kaplan–Meier univariate analysis.** Blue line represents the survival curve of *C9orf72* pathogenic expansion carriers (n = 319), while the orange line represents non-expanded patients (n = 3,970) after genotype correction.
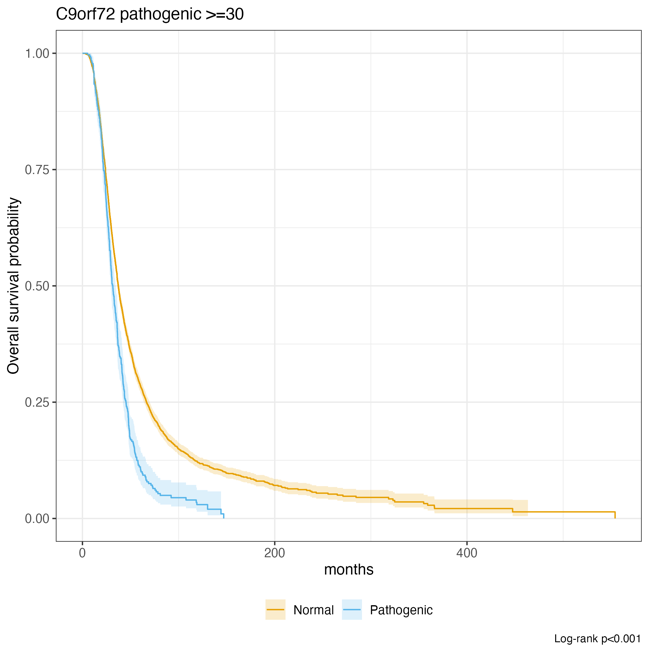


**Supplementary Table 20. ALS survival association with literature threshold analysis.**

**A. Pathogenic threshold**

| **RepeatID** | **Threshold** | **HR** | **SE** | **95% CI** | **P** | **Pbon** |
| --- | --- | --- | --- | --- | --- | --- |
| *C9ORF72* | 30 | 1.51 | 0.062 | 1.34 – 1.71 | 2.37E-11 | 3.79E-10 |
| *NIPA1* | 9 | 1.25 | 0.079 | 1.07 – 1.46 | 5.04E-03 | 0.081 |
| *ATXN1* | 39 | 0.46 | 0.71 | 0.11 – 1.84 | 0.27 | 1.00 |
| *ATXN2* | 33 | 1.13 | 0.24 | 0.70 – 1.83 | 0.61 | 1.00 |
| *ATXN8* | 80 | 0.94 | 0.17 | 0.68 – 1.31 | 0.72 | 1.00 |
| *DMPK* | 50 | 1.33 | 0.50 | 0.50 – 3.55 | 0.57 | 1.00 |
| *HTT* | 40 | 1.40 | 0.58 | 0.45 – 4.34 | 0.56 | 1.00 |
| *TCF4* | 80 | 1.12 | 0.079 | 0.96 – 1.30 | 0.16 | 1.00 |

**B Intermediate threshold**

| **RepeatID** | **Threshold** | **HR** | **SE** | **95% CI** | **P** | **Pbon** |
| --- | --- | --- | --- | --- | --- | --- |
| *ATXN1* | 33 | 1.03 | 0.052 | 0.93 – 1.14 | 0.56 | 1.00 |
| *ATXN2* | 29 | 1.20 | 0.11 | 0.97 – 1.48 | 0.093 | 1.00 |
| *ATXN8* | 51 | 0.83 | 0.27 | 0.49 – 1.41 | 0.49 | 1.00 |
| *C9ORF72* | 24 | 1.58 | 0.45 | 0.65 – 3.81 | 0.31 | 1.00 |
| *DMPK* | 35 | 0.93 | 0.21 | 0.62 – 1.40 | 0.74 | 1.00 |
| *GIPC1* | 32 | 0.68 | 0.45 | 0.28 – 1.64 | 0.39 | 1.00 |
| *HTT* | 27 | 0.99 | 0.069 | 0.87 – 1.14 | 0.90 | 1.00 |
| *TCF4* | 41 | 1.01 | 0.087 | 0.85 – 1.19 | 0.94 | 1.00 |

ALS survival association indicated by the hazard ratio (HR), standard error (SE) of the HR, 95% confidence interval (CI) of the HR, and p-value (P). A) Cox PH results for the pathogenic threshold. B) Cox PH results for the intermediate threshold. The p-value was corrected for the number of analysis types tested and the number of STRs tested (P_bon_).

**Supplementary Table 21. Sample counts and measures of central tendency for the progression analyses.**

**A.**

| **RepeatID** | **Threshold** | **N normal \| expanded** | **Mean (SD) normal \| expanded** | **Median [Min, Max] normal \| expanded** |
| --- | --- | --- | --- | --- |
| *AR* | Best threshold: 28 | 3985 \| 380 | 47.1 (± 43.1) \| 40.3 (± 39.2) | 34.3 [1.3, 554.0] \| 30.2 [5.0, 463.4] |
| *C9ORF72* | Pathogenic threshold: 30 | 3968 \| 319 | 47.3 (± 44.0) \| 35.8 (± 22.8) | 34.1 [1.3, 554.0] \| 30.3 [3.7, 146.9] |
|  | Premutation threshold: 24 | 4282 \| 5 | 46.4 (± 42.9) \| 34.7 (± 20.4) | 33.9 [1.3, 554.0] \| 32.6 [12.8, 58.8] |
|  | Best threshold: 24 | 3963 \| 324 | 47.3 (± 44.0) \| 35.7 (± 22.7) | 34.1 [1.3, 554.0] \| 30.4 [3.7, 146.9] |
| *NIPA1* | Pathogenic threshold: 9 | 4062 \| 200 | 46.8 (± 43.5) \| 39.7 (± 27.4) | 34.0 [1.3, 554.0] \| 30.8 [3.7, 151.0] |
|  | Best threshold: 10 | 4107 \| 155 | 46.8 (± 43.4) \| 38.4 (± 28.3) | 34.1 [1.3, 554.0] \| 29.3 [3.7, 151.0] |

**B.**

| **VariantID** | **Threshold** | **N normal \| expanded** | **Mean (SD) normal \| expanded** | **Median [Min, Max] normal \| expanded** |
| --- | --- | --- | --- | --- |
| *C9ORF72* | Pathogenic threshold: 30 | 3968 \| 319 | 61.0 (± 12.3) \| 58.6 (± 9.2) | 62.5 [18.0, 98.8] \| 59.4 [28.0, 86.9] |
|  | Premutation threshold: 24 | 4282 \| 5 | 60.8 (± 12.2) \| 60.6 (± 11.0) | 62.1 [18.0, 98.8] \| 57.7 [49.6, 73.3] |
|  | Best threshold: 24 | 3963 \| 324 | 61.0 (± 12.3) \| 58.6 (± 9.2) | 62.5 [18.0, 98.8] \| 59.4 [28.0, 86.9] |
| *TCF4* | Pathogenic threshold: 80 | 4104 \| 206 | 60.8 (± 12.1) \| 61.2 (± 12.3) | 62.1 [18.0, 98.8] \| 62.5 [21.6, 87.0] |
|  | Premutation threshold: 41 | 4134 \| 176 | 60.9 (± 12.1) \| 60.1 (± 12.3) | 62.2 [18.0, 98.8] \| 61.0 [23.6, 88.7] |
|  | Best threshold: 116 | 4287 \| 23 | 60.8 (± 12.1) \| 69.2 (± 11.9) | 62.0 [18.0, 98.8] \| 71.6 [34.1, 83.8] |

Overview of the number of samples analyzed in each comparison group from Figure 3B and C, along with their respective mean and median survival expressed in months (A) and age at onset expressed in years (B).

**Supplementary Table 22. ALS survival association with more *HTT* literature thresholds.**

| **RepeatID** | **Type** | **Threshold** | **HR** | **SE** | **95% CI** | **P** |
| --- | --- | --- | --- | --- | --- | --- |
| *HTT* | premutation | >= 27 & < 35 | 1.01 | 0.071 | 0.88 – 1.17 | 0.85 |
| *HTT* | premutation | >= 27 & < 36 | 1.00 | 0.070 | 0.87 – 1.15 | 0.99 |
| *HTT* | premutation | >= 36 & 40 | 0.78 | 0.38 | 0.37 – 1.64 | 0.51 |
| *HTT* | pathogenic | >= 37 | 0.90 | 0.32 | 0.48 – 1.67 | 0.74 |

Survival association of additional *HTT* pathogenic and premutation thresholds from literature indicated by the hazard ratio (HR), standard error (SE) of the HR, 95% confidence interval (CI) of the HR, and p-value (P).

**Supplementary Table 23. ALS age at onset association with literature threshold analysis.**

**A. Pathogenic threshold**

| **RepeatID** | **Threshold** | **Effect** | **SE** | **95% CI** | **P** | **Pbon** |
| --- | --- | --- | --- | --- | --- | --- |
| *C9ORF72* | 30 | -2.75 | 0.70 | -4.11 – -1.38 | 7.96E-05 | 1.43E-03 |
| *ATXN1* | 39 | 0.18 | 5.98 | -11.54 – 11.90 | 0.98 | 1.00 |
| *ATXN2* | 33 | -1.90 | 2.75 | -7.29 – 3.48 | 0.49 | 1.00 |
| *ATXN8* | 80 | 1.16 | 1.77 | -2.31 – 4.64 | 0.51 | 1.00 |
| *DMPK* | 50 | -0.26 | 5.35 | -10.74 – 10.23 | 0.96 | 1.00 |
| *HTT* | 40 | -1.01 | 5.97 | -12.70 – 10.69 | 0.87 | 1.00 |
| *NIPA1* | 9 | 0.17 | 0.87 | -1.53 – 1.87 | 0.84 | 1.00 |
| *RFC1* | 400 | -1.00 | 0.76 | -2.49 – 0.49 | 0.19 | 1.00 |
| *TCF4* | 80 | 0.87 | 0.85 | -0.81 – 2.54 | 0.31 | 1.00 |

**B. Intermediate threshold**

| **RepeatID** | **Threshold** | **Effect** | **SE** | **95% CI** | **P** | **Pbon** |
| --- | --- | --- | --- | --- | --- | --- |
| *ATXN1* | 33 | 0.23 | 0.56 | -0.87 – 1.32 | 0.68 | 1.00 |
| *ATXN2* | 29 | 1.10 | 1.19 | -1.23 – 3.42 | 0.35 | 1.00 |
| *ATXN8* | 51 | 0.87 | 2.55 | -4.14 – 5.87 | 0.73 | 1.00 |
| *C9ORF72* | 24 | -2.21 | 5.37 | -12.73 – 8.32 | 0.68 | 1.00 |
| *DMPK* | 35 | -0.55 | 2.27 | -4.99 – 3.90 | 0.81 | 1.00 |
| *GIPC1* | 32 | 7.49 | 4.52 | -1.38 – 16.36 | 0.098 | 1.00 |
| *HTT* | 27 | 1.38 | 0.74 | -0.069 – 2.84 | 0.062 | 1.00 |
| *TCF4* | 41 | -0.50 | 0.92 | -2.30 – 1.31 | 0.59 | 1.00 |

ALS age at onset association indicated by the effect size (Effect, log-odds ratio), standard error (SE) of the effect size, 95% confidence interval (CI) of the effect size and p-value (P). A) Linear regression results for the pathogenic threshold. B) Linear regression results for the intermediate threshold. The P_bon_ column represents the Bonferroni corrected p-value for the number of STRs tested.

**Supplementary Figure 18. ALS survival association of *C9orf72* with best threshold analysis.** A) Negative base-10 logarithm of the p-value (-10logP, blue) and hazard ratio (HR, orange) upon Cox proportional hazard analysis for each existing repeat size up to 90 repeat units in 4,368 Project MinE cases. The horizontal blue line represents the FDR corrected significant p-value threshold. B) HR and 95% confidence interval (grey) for each existing repeat size up to 90 repeat units. Horizontal line represents a HR of 1. The best threshold was identified as the repeat size of which the HR had the highest distance from the line drawn between the first and last HR of the significant repeat sizes (diagonal line). Vertical dashed line represents the best threshold at 24 repeat units in both plots.


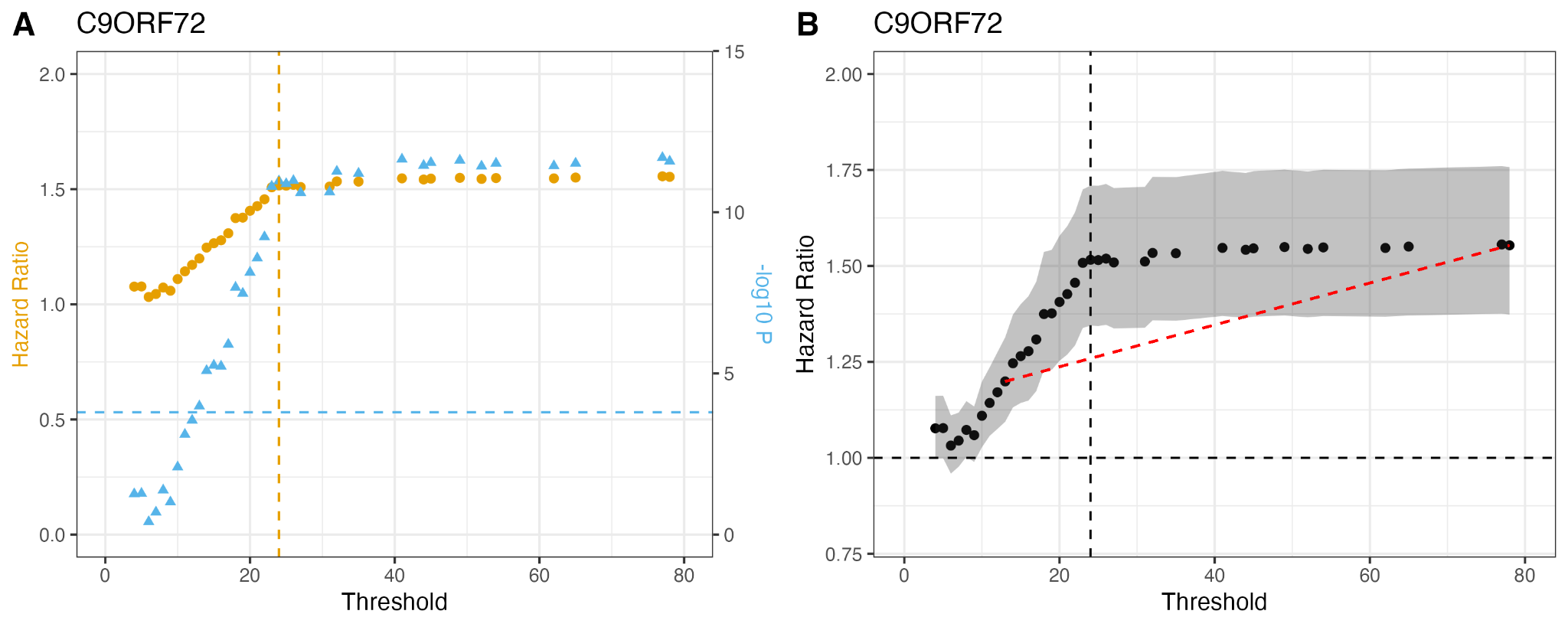


**Supplementary Table 24. ALS survival association with best threshold analysis.**

| **RepeatID** | **HR** | **SE** | **95% CI** | **Pfdr** | **# Thresholds** | **Pfdrbon** | **Threshold** |
| --- | --- | --- | --- | --- | --- | --- | --- |
| *C9ORF72* | 1.52 | 0.061 | 1.34 – 1.71 | 6.65E-11 | 287 | 1.66E-09 | 24 |
| *NIPA1* | 1.31 | 0.088 | 1.10 – 1.55 | 7.35E-03 | 5 | 0.18 | 10 |
| *AR* | 1.14 | 0.038 | 1.06 – 1.23 | 8.41E-03 | 23 | 0.21 | 28 |
| *ATN1* | 0.75 | 0.087 | 0.64 – 0.89 | 0.022 | 21 | 0.55 | 24 |
| *CSTB* | 1.20 | 0.058 | 1.07 – 1.35 | 0.026 | 18 | 0.64 | 3 |
| *HTT* | 0.90 | 0.035 | 0.84 – 0.96 | 0.027 | 29 | 0.67 | 19 |
| *JPH3* | 3.17 | 0.41 | 1.41 – 7.10 | 0.057 | 11 | 1.00 | 24 |
| *ATXN2* | 1.51 | 0.15 | 1.13 – 2.02 | 0.059 | 17 | 1.00 | 32 |
| *ATXN10* | 1.27 | 0.13 | 0.99 – 1.63 | 0.34 | 12 | 1.00 | 20 |
| *DMPK* | 1.10 | 0.039 | 1.02 – 1.19 | 0.37 | 43 | 1.00 | 15 |
| *RFC1* | 1.50 | 0.15 | 1.11 – 2.02 | 0.40 | 102 | 1.00 | 99 |
| *PPP2R2B* | 0.65 | 0.27 | 0.38 – 1.09 | 0.45 | 16 | 1.00 | 21 |
| *ATXN3* | 0.88 | 0.073 | 0.77 – 1.02 | 0.46 | 27 | 1.00 | 16 |
| *FXN* | 0.33 | 0.50 | 0.13 – 0.89 | 0.49 | 80 | 1.00 | 141 |
| *ATXN7* | 1.57 | 0.26 | 0.94 – 2.61 | 0.49 | 9 | 1.00 | 15 |
| *GLS* | 1.10 | 0.049 | 1.00 – 1.21 | 0.57 | 29 | 1.00 | 9 |
| *TCF4* | 1.18 | 0.085 | 1.00 – 1.39 | 0.71 | 118 | 1.00 | 85 |
| *ATXN1* | 0.70 | 0.24 | 0.44 – 1.11 | 0.72 | 13 | 1.00 | 37 |
| *GIPC1* | 0.83 | 0.14 | 0.63 – 1.10 | 0.79 | 26 | 1.00 | 16 |
| *NOP56* | 0.96 | 0.046 | 0.88 – 1.05 | 0.87 | 12 | 1.00 | 7 |
| *LRP12* | 0.88 | 0.078 | 0.75 – 1.02 | 0.92 | 19 | 1.00 | 16 |
| *DAB1* | 1.06 | 0.044 | 0.98 – 1.16 | 0.96 | 97 | 1.00 | 26 |
| *ATXN8* | 1.05 | 0.050 | 0.95 – 1.16 | 0.97 | 80 | 1.00 | 19 |
| *CBL* | 1.28 | 0.30 | 0.71 – 2.33 | 0.97 | 26 | 1.00 | 31 |
| *CACNA1A* | 0.75 | 0.23 | 0.48 – 1.18 | 0.99 | 12 | 1.00 | 15 |

Best threshold analysis of ALS survival was performed using Cox proportional hazards models in 4,368 Project MinE cases, reporting hazard ratios (HRs), their standard errors (SEs), and 95% confidence intervals (CIs). P_fdr_ represents the p-value FDR corrected for the number of thresholds tested. P_fdrbon_ represents P_fdr_ Bonferroni corrected for the number of STRs tested. ‘# Thresholds’ represents the number of thresholds tested per STR, and ‘Threshold’ represents the best threshold.

**Supplementary Table 25. ALS age at onset association with best threshold analysis.**

| **RepeatID** | **Effect** | **SE** | **95% CI** | **Pfdr** | **# Thresholds** | **Pfdrbon** | **Threshold** |
| --- | --- | --- | --- | --- | --- | --- | --- |
| *C9ORF72* | -2.75 | 0.69 | -4.10 – -1.39 | 5.26E-03 | 287 | 0.13 | 24 |
| *TCF4* | 8.98 | 2.50 | 4.09 – 13.87 | 7.79E-03 | 118 | 0.19 | 116 |
| *FXN* | 1.67 | 0.49 | 0.71 – 2.63 | 0.012 | 80 | 0.31 | 20 |
| *GLS* | -4.33 | 1.32 | -6.92 – -1.73 | 0.017 | 29 | 0.43 | 23 |
| *DMPK* | 1.63 | 0.52 | 0.60 – 2.65 | 0.025 | 43 | 0.63 | 10 |
| *ATXN10* | -1.37 | 0.49 | -2.33 – -0.42 | 0.038 | 12 | 0.96 | 14 |
| *CBL* | -5.18 | 2.30 | -9.70 – -0.66 | 0.13 | 26 | 1.00 | 28 |
| *CSTB* | -7.05 | 3.21 | -13.34 – -0.76 | 0.14 | 18 | 1.00 | 23 |
| *ATXN1* | 1.63 | 0.76 | 0.15 – 3.11 | 0.15 | 13 | 1.00 | 35 |
| *RFC1* | -0.75 | 0.37 | -1.48 – -0.025 | 0.19 | 102 | 1.00 | 58 |
| *ATN1* | -1.72 | 0.87 | -3.42 – -0.017 | 0.21 | 21 | 1.00 | 24 |
| *HTT* | 0.97 | 0.49 | 0.0027 – 1.93 | 0.21 | 29 | 1.00 | 24 |
| *DAB1* | 5.45 | 2.82 | -0.083 – 10.99 | 0.22 | 97 | 1.00 | 8 |
| *AR* | 0.55 | 0.29 | -0.024 – 1.12 | 0.23 | 23 | 1.00 | 21 |
| *ATXN7* | -7.84 | 4.27 | -16.21 – 0.53 | 0.25 | 9 | 1.00 | 16 |
| *LRP12* | -8.74 | 4.88 | -18.31 – 0.82 | 0.26 | 19 | 1.00 | 23 |
| *NOP56* | -8.15 | 4.89 | -17.73 – 1.44 | 0.30 | 12 | 1.00 | 13 |
| *ATXN3* | 7.62 | 4.87 | -1.93 – 17.18 | 0.33 | 27 | 1.00 | 37 |
| *JPH3* | -0.68 | 0.49 | -1.64 – 0.29 | 0.40 | 11 | 1.00 | 17 |
| *ATXN8* | 3.86 | 2.82 | -1.68 – 9.40 | 0.40 | 80 | 1.00 | 128 |
| *GIPC1* | 0.92 | 0.68 | -0.41 – 2.25 | 0.40 | 26 | 1.00 | 11 |
| *CACNA1A* | 0.40 | 0.37 | -0.33 – 1.12 | 0.55 | 12 | 1.00 | 13 |
| *PPP2R2B* | 2.82 | 2.61 | -2.30 – 7.95 | 0.55 | 16 | 1.00 | 21 |
| *ATXN2* | 0.84 | 1.05 | -1.22 – 2.90 | 0.71 | 17 | 1.00 | 28 |
| *NIPA1* | -0.45 | 0.87 | -2.16 – 1.26 | 0.82 | 5 | 1.00 | 8 |

Best threshold analysis of ALS age at onset was performed using linear regressions in 4,368 Project MinE cases, reporting the effect size (Effect, log-odds ratio), their standard errors (SEs), and 95% confidence intervals (CIs). P_fdr_ represents the p-value FDR corrected for the number of thresholds tested per STR. P_fdrbon_ represents the P_fdr_ Bonferroni corrected for the number of STRs tested. ‘# Thresholds’ represents the number of thresholds tested per STR, and ‘Threshold’ represents the best threshold.

**Supplementary Figure 19. ALS age at onset association of *C9orf72* with best threshold analysis.** A) Negative base-10 logarithm of the p-value (-10logP, blue) and effect size (log-odds ratio, orange) upon linear regression analysis for each existing repeat size up to 90 repeats units in 4,368 Project MinE cases. The horizontal blue line represents the FDR corrected significant p-value. B) OR and 95% confidence interval for each existing repeat size up to 90 repeat units. Horizontal line represents a HR of 1. The best threshold was identified as the repeat size of which the odds ratio had the highest distance from the line drawn between the first and last odds ratio of the significant repeat sizes (diagonal line). Vertical dashed line represents the best threshold at 24 repeat units in both plots.

**
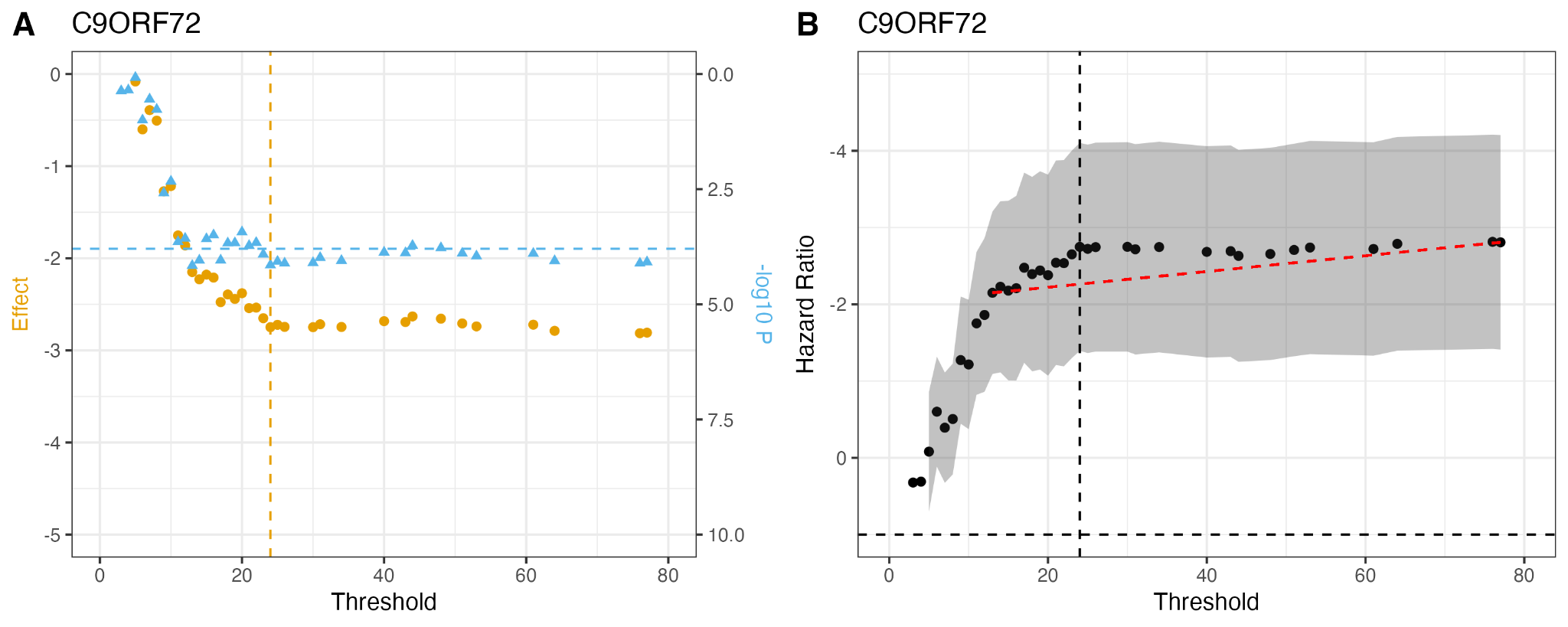
**

**Supplementary Table 26. ALS survival association with repeat size distribution analysis.**

**A. Maximum allele**

| **RepeatID** | **HR** | **SE** | **95% CI** | **P** | **Pbon** |
| --- | --- | --- | --- | --- | --- |
| *C9ORF72* | 1.00 | 0.000088 | 1.00 – 1.00 | 1.47E-08 | 7.48E-07 |
| *AR* | 1.02 | 0.0059 | 1.01 – 1.03 | 5.89E-04 | 0.030 |
| *ATN1* | 0.98 | 0.0076 | 0.97 – 1.00 | 0.036 | 1.00 |
| *NIPA1* | 1.08 | 0.039 | 1.00 – 1.17 | 0.040 | 1.00 |
| *ATXN2* | 1.02 | 0.0095 | 1.00 – 1.04 | 0.054 | 1.00 |
| *HTT* | 0.99 | 0.0048 | 0.98 – 1.00 | 0.055 | 1.00 |
| *ATXN3* | 0.99 | 0.0045 | 0.98 – 1.00 | 0.087 | 1.00 |
| *GLS* | 1.01 | 0.0040 | 1.00 – 1.01 | 0.098 | 1.00 |
| *TCF4* | 1.00 | 0.00084 | 1.00 – 1.00 | 0.23 | 1.00 |
| *DMPK* | 1.00 | 0.0027 | 1.00 – 1.01 | 0.23 | 1.00 |
| *NOP56* | 0.99 | 0.010 | 0.97 – 1.01 | 0.24 | 1.00 |
| *RFC1* | 1.00 | 0.00065 | 1.00 – 1.00 | 0.27 | 1.00 |
| *FXN* | 1.00 | 0.0013 | 1.00 – 1.00 | 0.32 | 1.00 |
| *ATXN7* | 1.01 | 0.015 | 0.98 – 1.04 | 0.50 | 1.00 |
| *PPP2R2B* | 1.00 | 0.0066 | 0.99 – 1.02 | 0.59 | 1.00 |
| *GIPC1* | 1.00 | 0.0063 | 0.98 – 1.01 | 0.62 | 1.00 |
| *LRP12* | 1.00 | 0.0087 | 0.99 – 1.02 | 0.64 | 1.00 |
| *CSTB* | 1.00 | 0.0095 | 0.99 – 1.02 | 0.67 | 1.00 |
| *CBL* | 1.00 | 0.0064 | 0.98 – 1.01 | 0.68 | 1.00 |
| *ATXN10* | 1.00 | 0.010 | 0.98 – 1.02 | 0.71 | 1.00 |
| *ATXN8* | 1.00 | 0.0015 | 1.00 – 1.00 | 0.74 | 1.00 |
| *JPH3* | 1.00 | 0.014 | 0.97 – 1.02 | 0.80 | 1.00 |
| *CACNA1A* | 1.00 | 0.015 | 0.97 – 1.03 | 0.82 | 1.00 |
| *ATXN1* | 1.00 | 0.0098 | 0.98 – 1.02 | 0.83 | 1.00 |
| *DAB1* | 1.00 | 0.00089 | 1.00 – 1.00 | 0.89 | 1.00 |

**B. Sum alleles**

| **RepeatID** | **HR** | **SE** | **95% CI** | **P** | **Pbon** |
| --- | --- | --- | --- | --- | --- |
| *C9ORF72* | 1.00 | 0.000087 | 1.00 – 1.00 | 1.12E-08 | 5.70E-07 |
| *AR* | 1.01 | 0.0047 | 1.01 – 1.02 | 1.56E-03 | 0.080 |
| *TCF4* | 1.00 | 0.00076 | 1.00 – 1.00 | 0.18 | 1.00 |
| *ATXN10* | 1.01 | 0.0066 | 0.99 – 1.02 | 0.24 | 1.00 |
| *ATXN3* | 1.00 | 0.0025 | 0.99 – 1.00 | 0.26 | 1.00 |
| *ATXN2* | 1.00 | 0.0034 | 1.00 – 1.01 | 0.28 | 1.00 |
| *DMPK* | 1.00 | 0.0020 | 1.00 – 1.01 | 0.29 | 1.00 |
| *LRP12* | 1.00 | 0.0035 | 1.00 – 1.01 | 0.31 | 1.00 |
| *NOP56* | 0.99 | 0.0065 | 0.98 – 1.01 | 0.36 | 1.00 |
| *FXN* | 1.00 | 0.0012 | 1.00 – 1.00 | 0.38 | 1.00 |
| *ARX_EIEE* | 0.99 | 0.0066 | 0.98 – 1.01 | 0.38 | 1.00 |
| *GLS* | 1.00 | 0.0027 | 1.00 – 1.01 | 0.39 | 1.00 |
| *ATN1* | 1.00 | 0.0037 | 0.99 – 1.00 | 0.40 | 1.00 |
| *ATXN7* | 1.00 | 0.0042 | 0.99 – 1.01 | 0.45 | 1.00 |
| *HTT* | 1.00 | 0.0032 | 0.99 – 1.00 | 0.48 | 1.00 |
| *CBL* | 1.00 | 0.0055 | 0.99 – 1.01 | 0.57 | 1.00 |
| *CACNA1A* | 1.00 | 0.0056 | 0.99 – 1.01 | 0.61 | 1.00 |
| *PPP2R2B* | 1.00 | 0.0052 | 0.99 – 1.01 | 0.76 | 1.00 |
| *ATXN1* | 1.00 | 0.0043 | 0.99 – 1.01 | 0.79 | 1.00 |
| *NIPA1* | 1.00 | 0.0072 | 0.98 – 1.01 | 0.80 | 1.00 |
| *JPH3* | 1.00 | 0.0087 | 0.98 – 1.02 | 0.83 | 1.00 |
| *CSTB* | 1.00 | 0.0084 | 0.99 – 1.02 | 0.83 | 1.00 |
| *ATXN8* | 1.00 | 0.0014 | 1.00 – 1.00 | 0.90 | 1.00 |
| *DAB1* | 1.00 | 0.00079 | 1.00 – 1.00 | 0.90 | 1.00 |
| *GIPC1* | 1.00 | 0.0045 | 0.99 – 1.01 | 0.90 | 1.00 |
| *RFC1* | 1.00 | 0.00038 | 1.00 – 1.00 | 0.93 | 1.00 |

Repeat size distribution analysis of ALS survival was performed using Cox proportional hazard analysis with the maximum allele size (A) and the sum of both alleles (B) as independent variables in 4,368 Project MinE cases, reporting the hazard ratio (HR), its standard error (SE), 95% confidence interval (CI) and p-value (P). The p-value was corrected for the number of analysis types tested and the number of STRs tested (P_bon_).

**Supplementary Table 27. ALS survival association of *AR* in the Norwegian cohort.**

| **RepeatID** | **Analysis** | **HR** | **SE** | **95% CI** | **P** |
| --- | --- | --- | --- | --- | --- |
| *AR* | Size distribution | 0.98 | 0.019 | 0.94 – 1.01 | 0.22 |
| *AR* | Best threshold: 28 | 0.84 | 0.20 | 0.57 – 1.24 | 0.39 |

Analysis type ‘size distribution’ is a cox PH association of survival using the maximum repeat size, represented by hazard ratio (HR), standard error (SE) of the HR, 95% confidence interval (CI) of the HR and p-value (P). Analysis type ‘best threshold’ is a Cox PH association of survival using the best threshold of 28 repeat units from the analysis in Project MinE. Schoenfeld residuals are added to check the proportional hazards assumption of equal hazards.

**Supplementary Table 28. ALS age at onset association with repeat size distribution analysis.**

**A. Maximum allele**

| **RepeatID** | **Effect** | **SE** | **95% CI** | **P** | **Pbon** |
| --- | --- | --- | --- | --- | --- |
| *C9ORF72* | -0.0035 | 0.0011 | -0.0056 – -0.0014 | 1.13E-03 | 0.057 |
| *FXN* | 0.039 | 0.013 | 0.013 – 0.065 | 3.29E-03 | 0.17 |
| *ATXN10* | -0.26 | 0.11 | -0.47 – -0.043 | 0.019 | 0.95 |
| *AR* | 0.14 | 0.063 | 0.013 – 0.26 | 0.031 | 1.00 |
| *DMPK* | 0.062 | 0.029 | 0.0051 – 0.12 | 0.033 | 1.00 |
| *CBL* | -0.13 | 0.065 | -0.25 – 0.00013 | 0.050 | 1.00 |
| *GLS* | -0.068 | 0.041 | -0.15 – 0.011 | 0.092 | 1.00 |
| *HTT* | 0.076 | 0.051 | -0.023 – 0.18 | 0.13 | 1.00 |
| *TCF4* | 0.012 | 0.0090 | -0.0062 – 0.029 | 0.20 | 1.00 |
| *LRP12* | -0.11 | 0.091 | -0.29 – 0.066 | 0.22 | 1.00 |
| *CSTB* | -0.12 | 0.11 | -0.33 – 0.095 | 0.28 | 1.00 |
| *NOP56* | -0.10 | 0.10 | -0.31 – 0.10 | 0.32 | 1.00 |
| *ATXN3* | -0.035 | 0.047 | -0.13 – 0.058 | 0.46 | 1.00 |
| *ATXN8* | 0.011 | 0.015 | -0.019 – 0.042 | 0.47 | 1.00 |
| *ATXN1* | -0.076 | 0.10 | -0.28 – 0.13 | 0.47 | 1.00 |
| *RFC1* | -0.0049 | 0.0070 | -0.019 – 0.0087 | 0.48 | 1.00 |
| *CACNA1A* | 0.098 | 0.17 | -0.23 – 0.42 | 0.55 | 1.00 |
| *DAB1* | 0.0051 | 0.0095 | -0.014 – 0.024 | 0.59 | 1.00 |
| *ATXN2* | 0.042 | 0.10 | -0.16 – 0.24 | 0.69 | 1.00 |
| *ATXN7* | -0.061 | 0.16 | -0.37 – 0.25 | 0.70 | 1.00 |
| *JPH3* | 0.054 | 0.15 | -0.24 – 0.35 | 0.72 | 1.00 |
| *GIPC1* | 0.013 | 0.062 | -0.11 – 0.13 | 0.84 | 1.00 |
| *ATN1* | -0.013 | 0.080 | -0.17 – 0.14 | 0.88 | 1.00 |
| *PPP2R2B* | -0.0018 | 0.072 | -0.14 – 0.14 | 0.98 | 1.00 |
| *NIPA1* | 0.0074 | 0.40 | -0.78 – 0.80 | 0.99 | 1.00 |

**B. Sum alleles**

| **RepeatID** | **Effect** | **SE** | **95% CI** | **P** | **Pbon** |
| --- | --- | --- | --- | --- | --- |
| *C9ORF72* | -0.0034 | 0.0011 | -0.0055 – -0.0013 | 1.32E-03 | 0.067 |
| *FXN* | 0.038 | 0.012 | 0.013 – 0.062 | 2.39E-03 | 0.12 |
| *CBL* | -0.14 | 0.056 | -0.25 – -0.034 | 0.010 | 0.51 |
| *ATXN10* | -0.16 | 0.070 | -0.30 – -0.028 | 0.018 | 0.92 |
| *ATXN7* | -0.090 | 0.045 | -0.18 – -0.00086 | 0.048 | 1.00 |
| *ATXN2* | 0.066 | 0.036 | -0.0034 – 0.14 | 0.062 | 1.00 |
| *DMPK* | 0.039 | 0.021 | -0.0027 – 0.081 | 0.067 | 1.00 |
| *AR* | 0.083 | 0.049 | -0.013 – 0.18 | 0.090 | 1.00 |
| *HTT* | 0.058 | 0.035 | -0.0100 – 0.13 | 0.094 | 1.00 |
| *NOP56* | -0.11 | 0.068 | -0.24 – 0.025 | 0.11 | 1.00 |
| *ARX_EIEE* | 0.11 | 0.073 | -0.029 – 0.26 | 0.12 | 1.00 |
| *TCF4* | 0.012 | 0.0082 | -0.0044 – 0.028 | 0.16 | 1.00 |
| *RFC1* | -0.0053 | 0.0041 | -0.013 – 0.0027 | 0.19 | 1.00 |
| *GLS* | -0.035 | 0.028 | -0.090 – 0.020 | 0.22 | 1.00 |
| *NIPA1* | 0.092 | 0.077 | -0.059 – 0.24 | 0.23 | 1.00 |
| *CSTB* | -0.11 | 0.091 | -0.28 – 0.072 | 0.24 | 1.00 |
| *GIPC1* | 0.047 | 0.049 | -0.048 – 0.14 | 0.33 | 1.00 |
| *JPH3* | 0.056 | 0.093 | -0.13 – 0.24 | 0.55 | 1.00 |
| *DAB1* | 0.0045 | 0.0083 | -0.012 – 0.021 | 0.59 | 1.00 |
| *ATXN1* | -0.022 | 0.045 | -0.11 – 0.066 | 0.63 | 1.00 |
| *ATXN8* | 0.0069 | 0.015 | -0.022 – 0.035 | 0.64 | 1.00 |
| *CACNA1A* | 0.018 | 0.061 | -0.10 – 0.14 | 0.77 | 1.00 |
| *ATXN3* | -0.0071 | 0.026 | -0.058 – 0.044 | 0.78 | 1.00 |
| *ATN1* | -0.0093 | 0.039 | -0.086 – 0.067 | 0.81 | 1.00 |
| *LRP12* | 0.0070 | 0.037 | -0.066 – 0.080 | 0.85 | 1.00 |
| *PPP2R2B* | 0.0097 | 0.056 | -0.099 – 0.12 | 0.86 | 1.00 |

Linear regression association statistics using the maximum allele size (A) and the sum of both alleles (B) as independent variables in 4,368 Project MinE cases, reporting the effect size (log-odds ratio), its standard error (SE), 95% confidence interval (CI) and p-value (P). The p-value was corrected for the number of analysis (n=2) and the number of STRs tested (P_bon_).

**Supplementary Table 29. ALS progression sensitivity analyses**

**A. ALS survival association with pathogenic literature threshold analysis with the Royston-Parmar model.**

| **RepeatID** | **Threshold** | **HR** | **95% CI** | **P** | **Pbon** |
| --- | --- | --- | --- | --- | --- |
| *C9ORF72* | 30 | 1.52 | 1.35 – 0.95 | 1.36E-11 | 2.58E-10 |
| *ATXN2* | 33 | 1.15 | 0.71 – 0.95 | 0.56 | 1.00 |
| *ATXN8* | 80 | 0.95 | 0.69 – 0.95 | 0.76 | 1.00 |
| *CSTB* | 30 | 0.74 | 0.31 – 0.95 | 0.50 | 1.00 |
| *DMPK* | 50 | 1.32 | 0.49 – 0.94 | 0.58 | 1.00 |
| *FXN* | 66 | 0.97 | 0.73 – 0.94 | 0.84 | 1.00 |
| *TCF4* | 80 | 1.12 | 0.97 – 0.95 | 0.12 | 1.00 |

**B. ALS survival association with intermediate literature threshold analysis with the Royston-Parmar model.**

| **RepeatID** | **Threshold** | **HR** | **95% CI** | **P** | **Pbon** |
| --- | --- | --- | --- | --- | --- |
| *AR* | 35 | 1.28 | 0.31 – 1.02 | 0.73 | 1.00 |
| *ATXN1* | 33 | 1.03 | 0.93 – 0.95 | 0.58 | 1.00 |
| *ATXN2* | 29 | 1.22 | 0.99 – 0.95 | 0.062 | 1.00 |
| *ATXN8* | 51 | 0.85 | 0.50 – 0.95 | 0.54 | 1.00 |
| *C9ORF72* | 24 | 1.59 | 0.66 – 0.95 | 0.30 | 1.00 |
| *CSTB* | 4 | 1.39 | 0.91 – 0.95 | 0.12 | 1.00 |
| *DMPK* | 35 | 0.92 | 0.61 – 0.94 | 0.70 | 1.00 |
| *FXN* | 35 | 1.17 | 0.68 – 0.94 | 0.58 | 1.00 |
| *GIPC1* | 32 | 0.66 | 0.27 – 0.95 | 0.35 | 1.00 |
| *HTT* | 27 | 0.99 | 0.87 – 0.94 | 0.90 | 1.00 |
| *TCF4* | 41 | 1.01 | 0.85 – 0.95 | 0.94 | 1.00 |

**C. ALS survival association with pathogenic threshold analysis without mode of inheritance.**

| **RepeatID** | **Threshold** | **HR** | **SE** | **95% CI** | **P** | **Pbon** |
| --- | --- | --- | --- | --- | --- | --- |
| *C9ORF72* | 30 | 1.51 | 0.062 | 1.34 – 1.71 | 2.37E-11 | 4.50E-10 |
| *NIPA1* | 9 | 1.25 | 0.079 | 1.07 – 1.46 | 4.07E-03 | 0.077 |
| *TCF4* | 80 | 1.13 | 0.075 | 0.97 – 1.31 | 0.11 | 1.00 |
| *ARX_EIEE* | 17 | 3.19 | 1.00 | 0.45 – 22.76 | 0.25 | 1.00 |
| *ATXN1* | 39 | 0.46 | 0.71 | 0.11 – 1.84 | 0.27 | 1.00 |
| *CSTB* | 30 | 0.75 | 0.45 | 0.31 – 1.80 | 0.52 | 1.00 |
| *HTT* | 40 | 1.40 | 0.58 | 0.45 – 4.34 | 0.56 | 1.00 |
| *DMPK* | 50 | 1.33 | 0.50 | 0.50 – 3.55 | 0.57 | 1.00 |
| *ATXN2* | 33 | 1.13 | 0.24 | 0.70 – 1.83 | 0.61 | 1.00 |
| *ATXN8* | 80 | 0.94 | 0.17 | 0.68 – 1.31 | 0.72 | 1.00 |
| *FXN* | 66 | 0.96 | 0.15 | 0.72 – 1.29 | 0.80 | 1.00 |

**D. ALS survival association with intermediate threshold analysis without mode of inheritance.**

| **RepeatID** | **Threshold** | **HR** | **SE** | **95% CI** | **P** | **Pbon** |
| --- | --- | --- | --- | --- | --- | --- |
| *ATXN1* | 33 | 1.03 | 0.052 | 0.93 – 1.14 | 0.56 | 1.00 |
| *ATXN2* | 29 | 1.20 | 0.11 | 0.97 – 1.48 | 0.093 | 1.00 |
| *ATXN8* | 51 | 0.83 | 0.27 | 0.49 – 1.41 | 0.49 | 1.00 |
| *C9ORF72* | 24 | 1.58 | 0.45 | 0.65 – 3.81 | 0.31 | 1.00 |
| *CSTB* | 4 | 1.39 | 0.22 | 0.91 – 2.12 | 0.13 | 1.00 |
| *DMPK* | 35 | 0.93 | 0.21 | 0.62 – 1.40 | 0.74 | 1.00 |
| *FXN* | 35 | 1.18 | 0.28 | 0.68 – 2.03 | 0.56 | 1.00 |
| *GIPC1* | 32 | 0.68 | 0.45 | 0.28 – 1.64 | 0.39 | 1.00 |
| *HTT* | 27 | 0.99 | 0.069 | 0.87 – 1.14 | 0.90 | 1.00 |
| *TCF4* | 41 | 1.01 | 0.087 | 0.85 – 1.19 | 0.94 | 1.00 |

**E. ALS age at onset association with pathogenic threshold analysis without mode of inheritance.**

| **RepeatID** | **Threshold** | **Effect** | **SE** | **95% CI** | **P** | **Pbon** |
| --- | --- | --- | --- | --- | --- | --- |
| *C9ORF72* | 30 | -2.75 | 0.70 | -4.11 – -1.38 | 7.96E-05 | 1.51E-03 |
| *FXN* | 66 | 3.56 | 1.52 | 0.57 – 6.54 | 0.020 | 0.37 |
| *ARX_EIEE* | 17 | -10.32 | 11.91 | -33.66 – 13.02 | 0.39 | 1.00 |
| *ATXN1* | 39 | 0.18 | 5.98 | -11.54 – 11.90 | 0.98 | 1.00 |
| *ATXN2* | 33 | -1.90 | 2.75 | -7.29 – 3.48 | 0.49 | 1.00 |
| *ATXN8* | 80 | 1.16 | 1.77 | -2.31 – 4.64 | 0.51 | 1.00 |
| *CSTB* | 30 | -1.05 | 4.53 | -9.93 – 7.83 | 0.82 | 1.00 |
| *DMPK* | 50 | -0.26 | 5.35 | -10.74 – 10.23 | 0.96 | 1.00 |
| *HTT* | 40 | -1.01 | 5.97 | -12.70 – 10.69 | 0.87 | 1.00 |
| *NIPA1* | 9 | 0.15 | 0.86 | -1.53 – 1.83 | 0.86 | 1.00 |
| *TCF4* | 80 | 0.90 | 0.81 | -0.69 – 2.49 | 0.27 | 1.00 |

**F. ALS age at onset association with intermediate threshold analysis without mode of inheritance.**

| **RepeatID** | **Threshold** | **Effect** | **SE** | **95% CI** | **P** | **Pbon** |
| --- | --- | --- | --- | --- | --- | --- |
| *ATXN1* | 33 | 0.22 | 0.56 | -0.87 – 1.32 | 0.69 | 1.00 |
| *ATXN2* | 29 | 1.10 | 1.19 | -1.23 – 3.42 | 0.35 | 1.00 |
| *ATXN8* | 51 | 0.87 | 2.55 | -4.14 – 5.87 | 0.73 | 1.00 |
| *C9ORF72* | 24 | -2.21 | 5.37 | -12.74 – 8.32 | 0.68 | 1.00 |
| *CSTB* | 4 | -1.39 | 2.31 | -5.92 – 3.14 | 0.55 | 1.00 |
| *DMPK* | 35 | -0.55 | 2.27 | -4.99 – 3.89 | 0.81 | 1.00 |
| *FXN* | 35 | 5.32 | 3.01 | -0.58 – 11.22 | 0.077 | 1.00 |
| *GIPC1* | 32 | 7.49 | 4.52 | -1.38 – 16.36 | 0.098 | 1.00 |
| *HTT* | 27 | 1.38 | 0.74 | -0.071 – 2.84 | 0.062 | 1.00 |
| *TCF4* | 41 | -0.66 | 0.92 | -2.46 – 1.15 | 0.47 | 1.00 |

**G. ALS survival association with log transformed repeat size distribution analysis.**

| **RepeatID** | **HR** | **SE** | **95% CI** | **P** | **Pbon** |
| --- | --- | --- | --- | --- | --- |
| *C9ORF72* | 1.08 | 0.012 | 1.05 – 1.10 | 1.55E-09 | 7.89E-08 |
| *AR* | 1.57 | 0.14 | 1.19 – 2.06 | 1.31E-03 | 0.067 |
| *CSTB* | 1.20 | 0.079 | 1.03 – 1.41 | 0.020 | 1.00 |
| *ATN1* | 0.75 | 0.14 | 0.57 – 0.99 | 0.040 | 1.00 |
| *HTT* | 0.81 | 0.11 | 0.66 – 1.00 | 0.048 | 1.00 |
| *ATXN2* | 1.61 | 0.25 | 0.99 – 2.61 | 0.056 | 1.00 |
| *NIPA1* | 1.86 | 0.33 | 0.97 – 3.59 | 0.063 | 1.00 |
| *ATXN3* | 0.86 | 0.085 | 0.73 – 1.02 | 0.087 | 1.00 |
| *GLS* | 1.09 | 0.060 | 0.97 – 1.23 | 0.13 | 1.00 |
| *RFC1* | 1.03 | 0.021 | 0.99 – 1.07 | 0.17 | 1.00 |
| *DMPK* | 1.05 | 0.038 | 0.97 – 1.13 | 0.23 | 1.00 |
| *TCF4* | 1.04 | 0.033 | 0.97 – 1.11 | 0.27 | 1.00 |
| *FXN* | 0.96 | 0.037 | 0.89 – 1.03 | 0.27 | 1.00 |
| *NOP56* | 0.93 | 0.066 | 0.82 – 1.06 | 0.30 | 1.00 |
| *PABPN1* | 3.70 | 1.32 | 0.28 – 49.15 | 0.32 | 1.00 |
| *PPP2R2B* | 1.07 | 0.086 | 0.91 – 1.27 | 0.41 | 1.00 |
| *GIPC1* | 0.91 | 0.14 | 0.70 – 1.19 | 0.48 | 1.00 |
| *ATXN7* | 1.12 | 0.17 | 0.80 – 1.56 | 0.52 | 1.00 |
| *LRP12* | 1.05 | 0.10 | 0.86 – 1.29 | 0.61 | 1.00 |
| *ATXN10* | 1.06 | 0.16 | 0.77 – 1.45 | 0.73 | 1.00 |
| *CBL* | 0.97 | 0.10 | 0.79 – 1.18 | 0.75 | 1.00 |
| *DAB1* | 1.01 | 0.029 | 0.95 – 1.07 | 0.75 | 1.00 |
| *JPH3* | 0.93 | 0.22 | 0.60 – 1.44 | 0.76 | 1.00 |
| *ARX_EIEE* | 2.67 | 3.70 | 0.0019 – 3781.28 | 0.79 | 1.00 |
| *ATXN1* | 0.93 | 0.31 | 0.50 – 1.72 | 0.82 | 1.00 |
| *CACNA1A* | 0.97 | 0.17 | 0.69 – 1.36 | 0.85 | 1.00 |
| *ATXN8* | 1.01 | 0.056 | 0.90 – 1.12 | 0.92 | 1.00 |

**H. ALS age at onset association with log transformed repeat size distribution analysis.**

| **RepeatID** | **Effect** | **SE** | **95% CI** | **P** | **Pbon** |
| --- | --- | --- | --- | --- | --- |
| *C9ORF72* | -0.43 | 0.13 | -0.68 – -0.17 | 1.28E-03 | 0.065 |
| *FXN* | 1.07 | 0.38 | 0.32 – 1.82 | 5.37E-03 | 0.27 |
| *DMPK* | 1.09 | 0.40 | 0.31 – 1.87 | 6.15E-03 | 0.31 |
| *ATXN10* | -4.28 | 1.70 | -7.62 – -0.94 | 0.012 | 0.61 |
| *PABPN1* | 32.62 | 13.04 | 7.05 – 58.19 | 0.012 | 0.63 |
| *AR* | 3.00 | 1.46 | 0.14 – 5.85 | 0.040 | 1.00 |
| *CBL* | -1.90 | 1.05 | -3.96 – 0.15 | 0.070 | 1.00 |
| *HTT* | 1.61 | 1.12 | -0.58 – 3.80 | 0.15 | 1.00 |
| *TCF4* | 0.42 | 0.35 | -0.26 – 1.09 | 0.23 | 1.00 |
| *GLS* | -0.76 | 0.64 | -2.02 – 0.49 | 0.23 | 1.00 |
| *LRP12* | -1.01 | 1.10 | -3.18 – 1.15 | 0.36 | 1.00 |
| *NOP56* | -0.59 | 0.71 | -1.98 – 0.79 | 0.40 | 1.00 |
| *ARX_EIEE* | -15.61 | 18.66 | -52.19 – 20.97 | 0.40 | 1.00 |
| *ATXN3* | -0.73 | 0.90 | -2.49 – 1.03 | 0.42 | 1.00 |
| *ATXN1* | -2.71 | 3.33 | -9.24 – 3.83 | 0.42 | 1.00 |
| *DAB1* | 0.24 | 0.31 | -0.37 – 0.85 | 0.45 | 1.00 |
| *ATXN8* | 0.36 | 0.60 | -0.82 – 1.55 | 0.55 | 1.00 |
| *CACNA1A* | 1.01 | 1.87 | -2.64 – 4.67 | 0.59 | 1.00 |
| *JPH3* | 1.02 | 2.34 | -3.57 – 5.62 | 0.66 | 1.00 |
| *ATXN2* | 1.01 | 2.67 | -4.23 – 6.24 | 0.71 | 1.00 |
| *ATXN7* | -0.54 | 1.81 | -4.09 – 3.01 | 0.77 | 1.00 |
| *RFC1* | -0.050 | 0.23 | -0.49 – 0.39 | 0.83 | 1.00 |
| *GIPC1* | 0.27 | 1.43 | -2.53 – 3.07 | 0.85 | 1.00 |
| *CSTB* | -0.13 | 0.93 | -1.96 – 1.70 | 0.89 | 1.00 |
| *PPP2R2B* | 0.069 | 0.92 | -1.74 – 1.88 | 0.94 | 1.00 |
| *ATN1* | 0.10 | 1.49 | -2.81 – 3.01 | 0.95 | 1.00 |
| *NIPA1* | -0.038 | 3.43 | -6.76 – 6.69 | 0.99 | 1.00 |

Several sensitivity analyses for ALS progression in 4,368 Project MinE cases represented by the effect size (Effect), standard error (SE) of the effect size, 95% confidence interval (CI) of the effect size, and p-value (P). A, B) Roston-Parmar survival association statistics. C, D, E, F) Progression association statistics with allelic dosages based on literature thresholds. G, H) Progression association statistics using the log transformed maximum allele size as independent variable. The p-value was corrected for the number of analysis types tested and the number of STRs tested (P_bon_).

**Supplementary Note on genotyping assessment**

Visual inspection of the aligned reads to the reference with REViewer revealed different types of inaccurate genotyping (Supplementary Table 1, column M).^1^ Reads with a poor alignment to the reference were observed in many of these STRs, i.e. *AFF2*, *FMR1*, *NOTCH2NLC*, *PHOX2B*, *SAMD12*, *SOX*, *STMN2* and *ZNF713,* and resulted in an overestimation of the repeat size (Supplementary Fig. 20 and 21). Other types of inaccurate genotyping identified were indels and motif interruptions, which often resulted in alleles with a low(er) number of consistent reads or high(er) number of non-consistent reads. *TBP*, for example, had a high number of alleles categorised as LCTNC because of motif interruptions that resembled the flanking sequence (Supplementary Fig. 22). Though motif interruptions occurred in many disease-associated STRs, only those in *TBP* and *CNBP* (Supplementary Fig. 23) induced failed genotyping. Failed genotyping in *CNBP* was also caused by an indel. Evidence for this was seen in nearly all inspected alleles but not always resulted in failed genotyping. In *NOTCH2NLC*, and to a lesser extent in *ATXN8*, four alleles per individual were observed instead of the expected two (Supplementary Fig. 24). This is likely of biological origin, such as mosaicism or the presence of gene paralogs.^2–4^ When only a few reads with more than two distinct repeat sizes were observed, as in *STMN2*, this could have either a biological origin or a technical origin, such as sequencing slippage. Since ExpansionHunter is limited to generating only two alleles, this resulted in incomplete or failed genotyping. Motif changes were a source of failed genotyping in *BEAN1* (Supplementary Fig. 25). ExpansionHunter mistakenly considered the change in motif as the start of the flanking sequence. Visual inspection of alleles longer than the read length also revealed motif changes in other repeats. ExpansionHunter DeNovo was used to automatically identify motif changes, and this revealed a substantial number of individuals with motif changes in *BEAN1* and *RFC1*, and also a number of motif changes in *ATXN8*, *C9orf72*, *DAB1*, *FXN* and *SAMD12* (Supplementary Table 10).

Although structural variants can lead to genotyping failures, this effect was not consistent within a given repeat or across different repeats. For example, motif interruptions in *TBP* were present in all alleles, whereas only a subset of alleles was reported to have less consistent than non-consistent reads (LCTNC) with genotyping failures observed in only a portion of the alleles identified as LCTNC. Also, genotyping failures could not be attributed solely to a single repeat parameter or combination of parameters. This makes automated identification of STR genotyping failures and their underlying causes highly challenging. Individual inspection of STR genotyping is therefore strongly recommended prior to drawing conclusions based on repeat size in short-read sequencing data. This can be achieved through visual inspection of allele read alignment using the seven binary repeat parameters, along with a representative sample of repeat size. This approach allows for identification of genotyping failure types and enables estimation of the extent of genotyping errors.

**Supplementary Figure 20.** **REViewer example *SAMD12*.** Read-aligned plot of the *SAMD12* STR locus from an individual exhibiting alleles containing 16 and 29 repeat units, as determined by ExpansionHunter. ​​The two reads in the black rectangles are considered as evidence of the allele with 29 repeat units, but the reads have a poor alignment to the reference. The reads in the red rectangles actually have 19 repeat units, because more units are hidden in the black line between the repeat and the right flank. The true genotype of this individual is 16 and 19 repeat units.

**
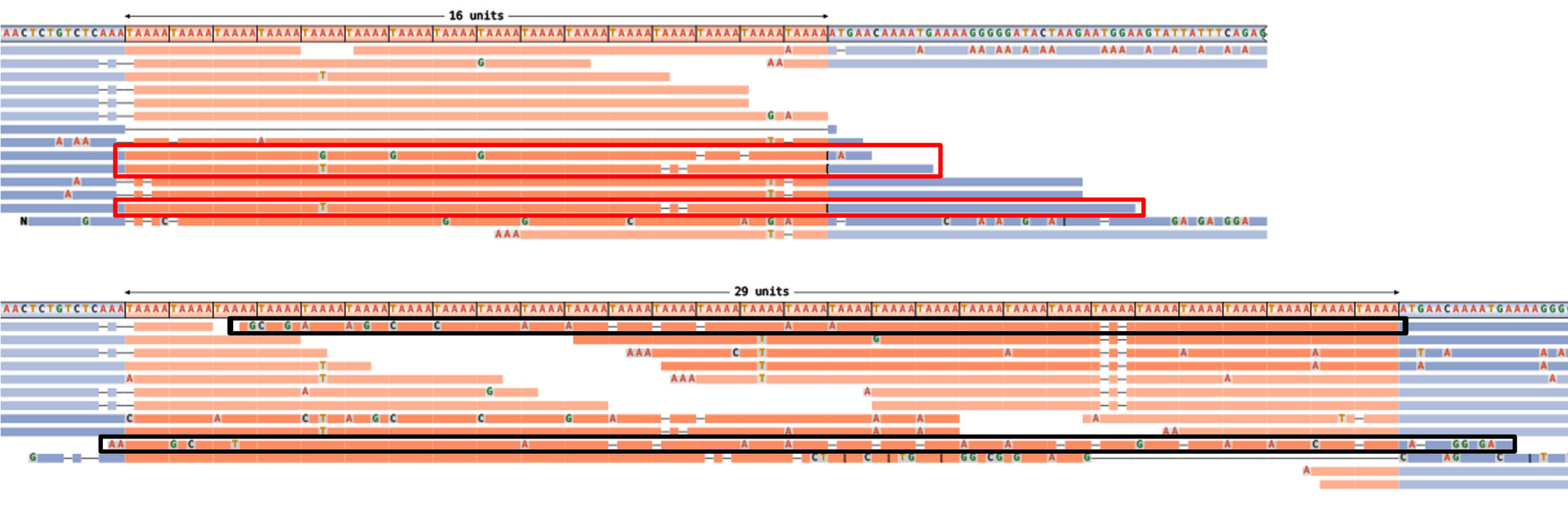
**

**Supplementary Figure 21. REViewer example *STMN2*.** Read-aligned plot of the *STMN2* STR locus from an individual exhibiting alleles containing 24 and 31 repeat units, as determined by ExpansionHunter. The reads considered as evidence of the allele with 29 repeat units (black rectangle) have a poor alignment to the reference. In general, there are many mutations in the *STMN2* reads, especially T to C conversions, both in the flanks as well as the STR. Often a few reads with a different number of repeats than reported by the bi-allelic call from ExpansionHunter are seen (red arrows).

**
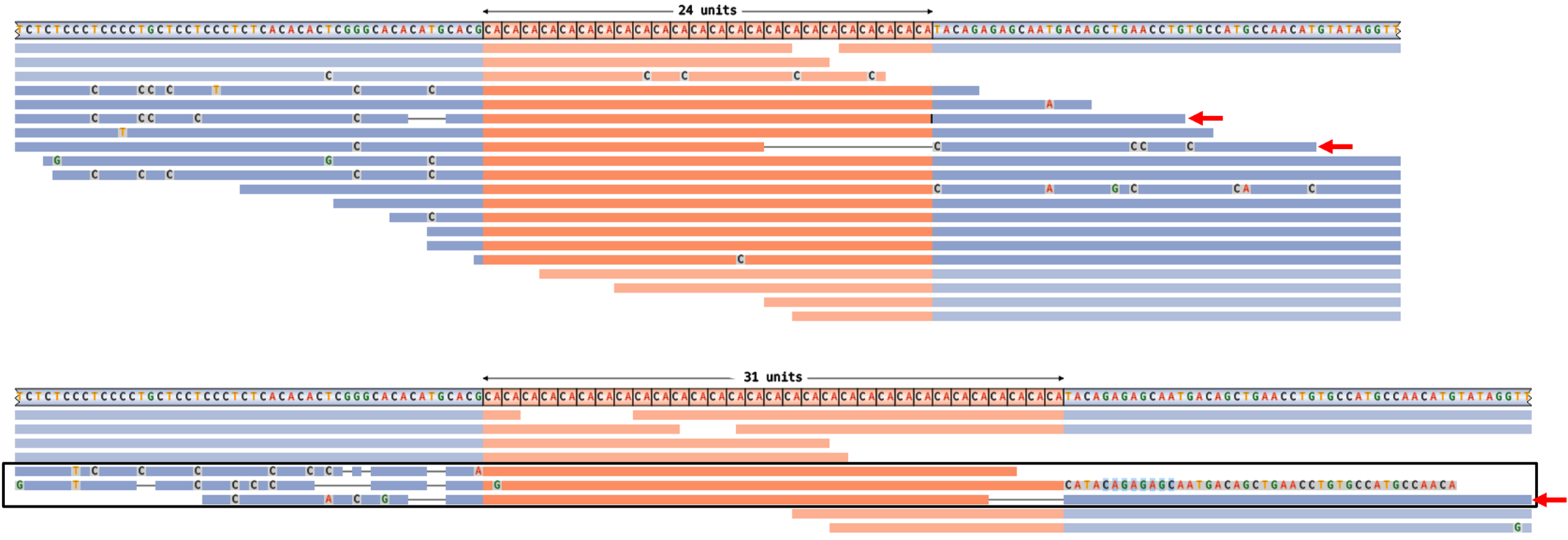
**

**Supplementary Figure 22. REViewer example *TBP*.** Read-aligned plot of the *TBP* STR locus from an individual exhibiting alleles containing 34 and 36 repeat units, as determined by ExpansionHunter. The reads in the rectangles are misaligned. This is caused by the motif interruption which is mistakenly seen as flanking region. The misaligned reads were spanning reads with less repeats than called by ExpansionHunter, so as ‘less consistent than non-consistent’ (LCTNC). Despite the misaligned reads, ExpansionHunter genotyped both alleles correctly.

**
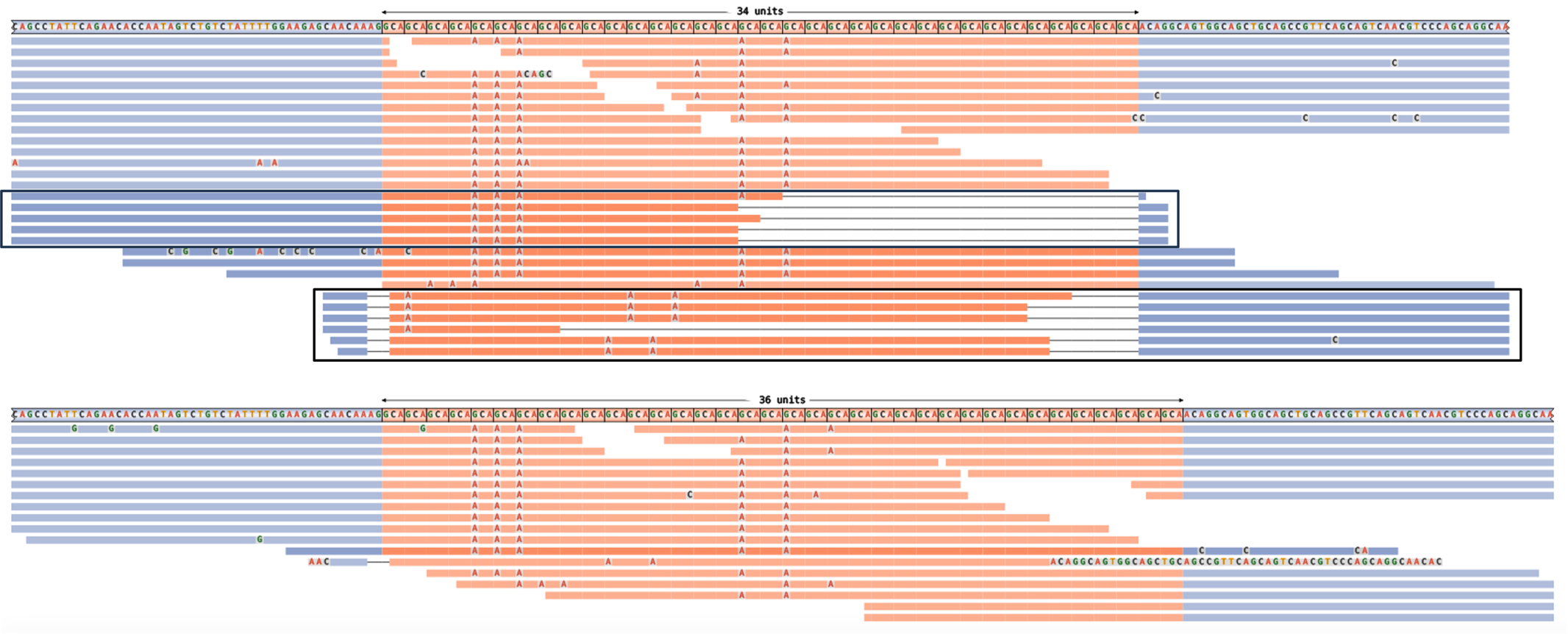
**

**Supplementary Figure 23. REViewer example *CNBP*.** Read-aligned plot of the *CNBP* STR locus from an individual exhibiting alleles containing 7 and 15 repeat units, as determined by ExpansionHunter. The reads in the black rectangle are considered as evidence for 7 CAGG repeats, but the motif interruption in the CAGG repeat is in these reads mistakenly considered to be the CAGA repeat following the CAGG repeat. The true CAGG genotype of this individual is homozygous 15 CAGG repeats units. The reads in the red rectangle do not align to the *CNBP* repeat and point to an indel nearby. These reads occasionally lead to an incorrectly inferred genotype with 12 CAGG repeat units.

**
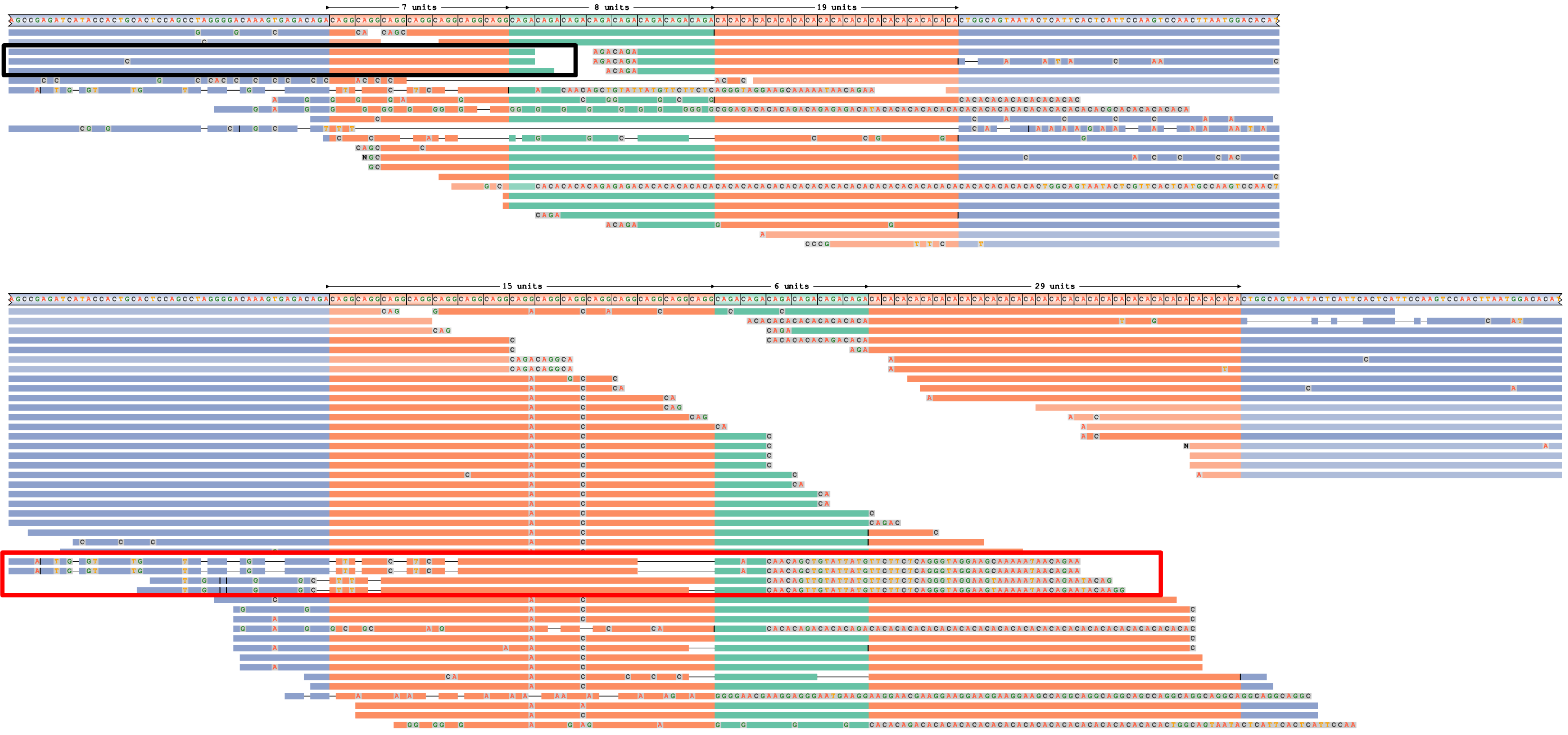
**

**Supplementary Figure 24. REViewer example *NOTCH2NLC*.** Read-aligned plot of the *NOTCH2NLC* STR locus from an individual exhibiting alleles containing 10 and 22 repeat units, as determined by ExpansionHunter. The reads in the black and red rectangle exemplify that also an allele with 7 and 22 repeat units exist, respectively.

**
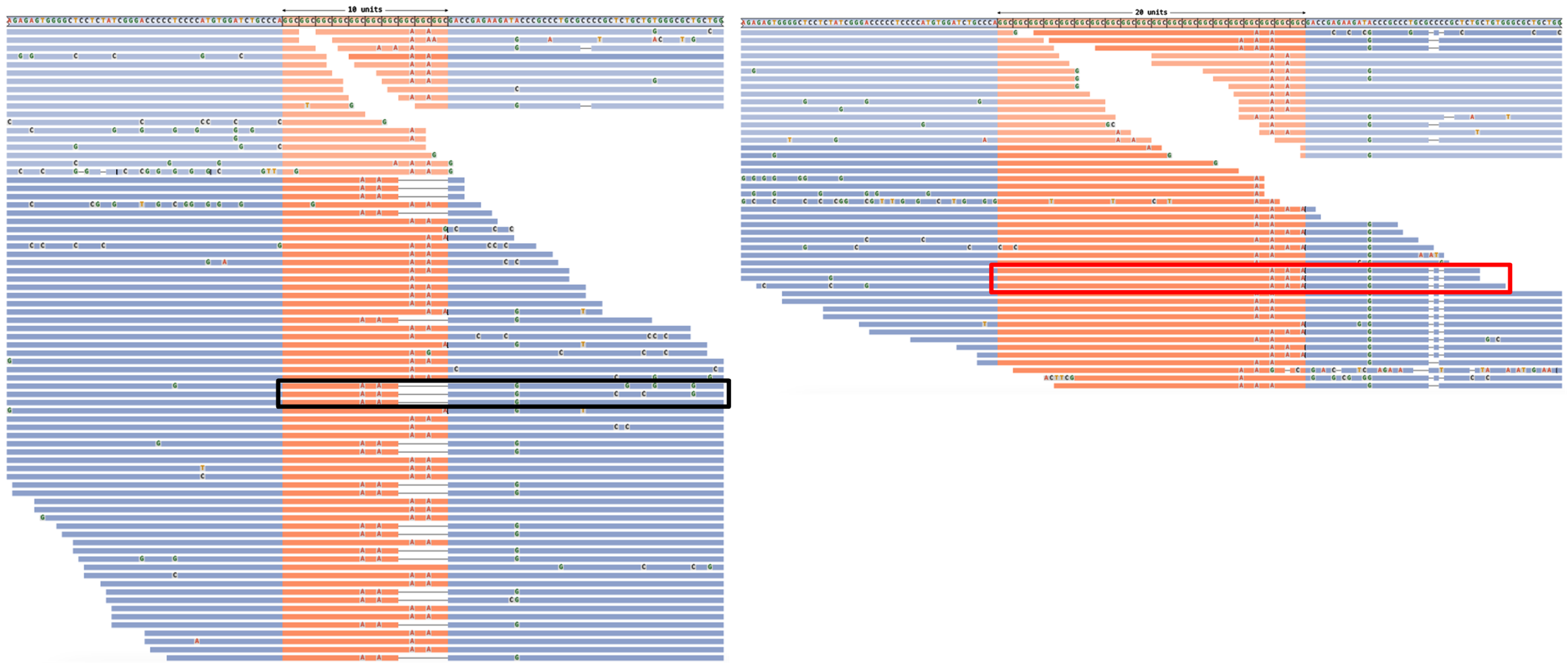
**

**Supplementary Figure 25. REViewer example *BEAN1*.** Read-aligned plot of the *BEAN1* STR locus from an individual exhibiting alleles containing 11 and 52 repeat units, as determined by ExpansionHunter. A single bp deletion and the subsequent motif change to TGAAA in one allele (black rectangle in bottom allele) causes ExpansionHunter to incorrectly genotype one allele with 11 repeat units (top allele). Indels at the junction between the repeat region and the right flank are observed in many individuals. Due to the absence of spanning reads, this individual most likely carries two alleles that exceed the read length, each exhibiting a distinct motif change pattern.


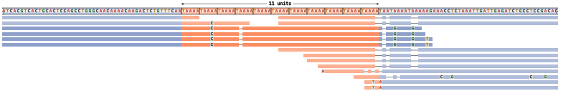


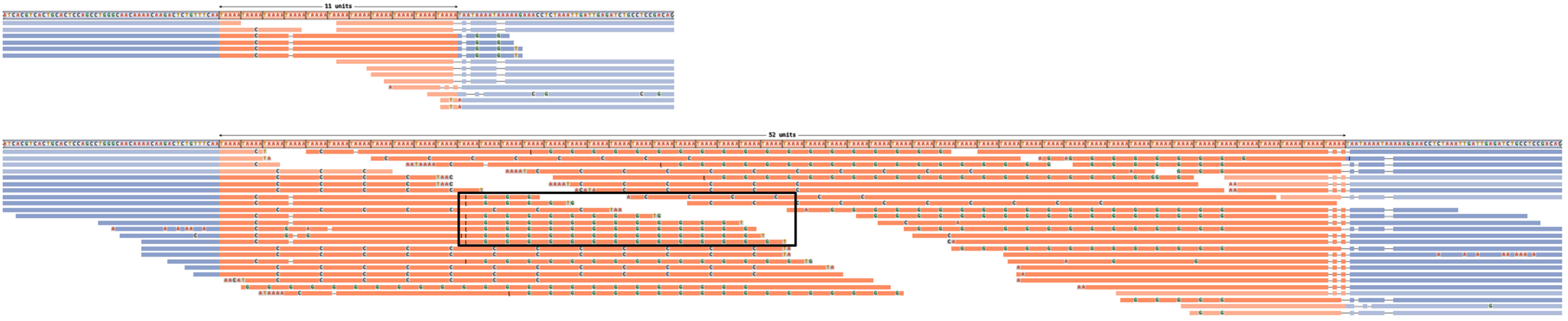


**References Supplementary Note**

1. Dolzhenko E, Weisburd B, Ibañez K, et al. REViewer: haplotype-resolved visualization of read alignments in and around tandem repeats. *Genome Med*. 2022;14(1):84. doi:10.1186/s13073-022-01085-z

2. Martins S, Seixas AI, Magalhães P, Coutinho P, Sequeiros J, Silveira I. Haplotype diversity and somatic instability in normal and expanded SCA8 alleles. *Am J Med Genet B Neuropsychiatr Genet*. 2005;139B(1):109-114. doi:10.1002/ajmg.b.30235

3. Fiddes IT, Lodewijk GA, Mooring M, et al. Human-Specific NOTCH2NL Genes Affect Notch Signaling and Cortical Neurogenesis. *Cell*. 2018;173(6):1356-1369.e22. doi:10.1016/j.cell.2018.03.051

4. Suzuki IK, Gacquer D, Van Heurck R, et al. Human-Specific NOTCH2NL Genes Expand Cortical Neurogenesis through Delta/Notch Regulation. *Cell*. 2018;173(6):1370-1384.e16. doi:10.1016/j.cell.2018.03.067
